# Supplementary material for: Genome-wide identification, characterization and gene expression of BES1 transcription factor family in grapevine (Vitis vinifera L.)
Source: Sci Rep. 2023 Jan 5;13:240. doi: 10.1038/s41598-022-24407-y (PMC9816167; doi:10.1038/s41598-022-24407-y)
Supplement: Supplementary file 3 — Supplementary Information. [file 41598_2022_24407_MOESM3_ESM.zip › Vvi_Vvi/Vitis_vinifera.PN40024.v4.dna_sm.toplevel.fa.vs.Vitis_vinifera.PN40024.v4.dna_sm.toplevel.fa.html/6.html]

|  |  |  |  |  |  |  |  |  |  |  |  |  |  |
| --- | --- | --- | --- | --- | --- | --- | --- | --- | --- | --- | --- | --- | --- |
| Duplication depth | Reference chromosome | Collinear blocks | | | | | | | | | | | |
| 0 | Vitvi06g04002\_t001 |  |  |  |  |  |  |  |  |  |  |  |  |
| 0 | Vitvi06g04003\_t001 |  |  |  |  |  |  |  |  |  |  |  |  |
| 0 | Vitvi06g04004\_t001 |  |  |  |  |  |  |  |  |  |  |  |  |
| 0 | Vitvi06g00007\_t001 |  |  |  |  |  |  |  |  |  |  |  |  |
| 0 | Vitvi06g00008\_t001 |  |  |  |  |  |  |  |  |  |  |  |  |
| 0 | Vitvi06g00009\_t001 |  |  |  |  |  |  |  |  |  |  |  |  |
| 0 | Vitvi06g00011\_t001 |  |  |  |  |  |  |  |  |  |  |  |  |
| 0 | Vitvi06g00012\_t001 |  |  |  |  |  |  |  |  |  |  |  |  |
| 0 | Vitvi06g00013\_t001 |  |  |  |  |  |  |  |  |  |  |  |  |
| 0 | Vitvi06g00015\_t001 |  |  |  |  |  |  |  |  |  |  |  |  |
| 0 | Vitvi06g00016\_t001 |  |  |  |  |  |  |  |  |  |  |  |  |
| 0 | Vitvi06g00017\_t001 |  |  |  |  |  |  |  |  |  |  |  |  |
| 0 | Vitvi06g00018\_t002 |  |  |  |  |  |  |  |  |  |  |  |  |
| 0 | Vitvi06g00019\_t001 |  |  |  |  |  |  |  |  |  |  |  |  |
| 0 | Vitvi06g04005\_t001 |  |  |  |  |  |  |  |  |  |  |  |  |
| 0 | Vitvi06g04006\_t001 |  |  |  |  |  |  |  |  |  |  |  |  |
| 0 | Vitvi06g00020\_t001 |  |  |  |  |  |  |  |  |  |  |  |  |
| 1 | Vitvi06g00021\_t001 |  | Vitvi08g01836\_t001 |  |  |  |  |  |  |  |  |  |  |
| 1 | Vitvi06g00022\_t001 |  | \| \| |  |  |  |  |  |  |  |  |  |  |
| 1 | Vitvi06g00024\_t001 |  | Vitvi08g01834\_t001 |  |  |  |  |  |  |  |  |  |  |
| 1 | Vitvi06g00026\_t001 |  | \| \| |  |  |  |  |  |  |  |  |  |  |
| 1 | Vitvi06g00027\_t001 |  | \| \| |  |  |  |  |  |  |  |  |  |  |
| 1 | Vitvi06g01573\_t001 |  | \| \| |  |  |  |  |  |  |  |  |  |  |
| 1 | Vitvi06g00028\_t001 |  | \| \| |  |  |  |  |  |  |  |  |  |  |
| 1 | Vitvi06g00029\_t001 |  | Vitvi08g01817\_t003 |  |  |  |  |  |  |  |  |  |  |
| 1 | Vitvi06g00030\_t001 |  | \| \| |  |  |  |  |  |  |  |  |  |  |
| 1 | Vitvi06g00031\_t001 |  | \| \| |  |  |  |  |  |  |  |  |  |  |
| 1 | Vitvi06g01574\_t001 |  | \| \| |  |  |  |  |  |  |  |  |  |  |
| 1 | Vitvi06g00032\_t001 |  | \| \| |  |  |  |  |  |  |  |  |  |  |
| 1 | Vitvi06g01575\_t001 |  | Vitvi08g01816\_t001 |  |  |  |  |  |  |  |  |  |  |
| 1 | Vitvi06g04007\_t001 |  | \| \| |  |  |  |  |  |  |  |  |  |  |
| 1 | Vitvi06g04008\_t001 |  | \| \| |  |  |  |  |  |  |  |  |  |  |
| 1 | Vitvi06g00034\_t001 |  | \| \| |  |  |  |  |  |  |  |  |  |  |
| 1 | Vitvi06g00035\_t001 |  | \| \| |  |  |  |  |  |  |  |  |  |  |
| 1 | Vitvi06g00036\_t001 |  | \| \| |  |  |  |  |  |  |  |  |  |  |
| 1 | Vitvi06g04009\_t001 |  | \| \| |  |  |  |  |  |  |  |  |  |  |
| 1 | Vitvi06g00037\_t001 |  | \| \| |  |  |  |  |  |  |  |  |  |  |
| 1 | Vitvi06g04010\_t001 |  | \| \| |  |  |  |  |  |  |  |  |  |  |
| 1 | Vitvi06g04011\_t001 |  | \| \| |  |  |  |  |  |  |  |  |  |  |
| 1 | Vitvi06g04012\_t001 |  | \| \| |  |  |  |  |  |  |  |  |  |  |
| 1 | Vitvi06g00039\_t001 |  | \| \| |  |  |  |  |  |  |  |  |  |  |
| 1 | Vitvi06g00040\_t001 |  | \| \| |  |  |  |  |  |  |  |  |  |  |
| 1 | Vitvi06g00041\_t001 |  | \| \| |  |  |  |  |  |  |  |  |  |  |
| 1 | Vitvi06g00042\_t001 |  | \| \| |  |  |  |  |  |  |  |  |  |  |
| 1 | Vitvi06g01580\_t001 |  | \| \| |  |  |  |  |  |  |  |  |  |  |
| 1 | Vitvi06g01581\_t001 |  | \| \| |  |  |  |  |  |  |  |  |  |  |
| 1 | Vitvi06g00043\_t001 |  | \| \| |  |  |  |  |  |  |  |  |  |  |
| 1 | Vitvi06g04013\_t001 |  | \| \| |  |  |  |  |  |  |  |  |  |  |
| 1 | Vitvi06g01582\_t001 |  | Vitvi08g02369\_t001 |  |  |  |  |  |  |  |  |  |  |
| 1 | Vitvi06g04014\_t001 |  | \| \| |  |  |  |  |  |  |  |  |  |  |
| 1 | Vitvi06g00044\_t001 |  | \| \| |  |  |  |  |  |  |  |  |  |  |
| 1 | Vitvi06g00045\_t001 |  | \| \| |  |  |  |  |  |  |  |  |  |  |
| 1 | Vitvi06g00046\_t002 |  | Vitvi08g01807\_t001 |  |  |  |  |  |  |  |  |  |  |
| 1 | Vitvi06g04015\_t001 |  | \| \| |  |  |  |  |  |  |  |  |  |  |
| 1 | Vitvi06g00048\_t001 |  | \| \| |  |  |  |  |  |  |  |  |  |  |
| 1 | Vitvi06g00049\_t001 |  | \| \| |  |  |  |  |  |  |  |  |  |  |
| 1 | Vitvi06g00050\_t001 |  | \| \| |  |  |  |  |  |  |  |  |  |  |
| 1 | Vitvi06g00051\_t002 |  | Vitvi08g01800\_t001 |  |  |  |  |  |  |  |  |  |  |
| 1 | Vitvi06g00052\_t001 |  | Vitvi08g01965\_t001 |  |  |  |  |  |  |  |  |  |  |
| 1 | Vitvi06g00053\_t001 |  | Vitvi08g01798\_t001 |  |  |  |  |  |  |  |  |  |  |
| 1 | Vitvi06g01583\_t001 |  | \| \| |  |  |  |  |  |  |  |  |  |  |
| 1 | Vitvi06g00054\_t001 |  | \| \| |  |  |  |  |  |  |  |  |  |  |
| 1 | Vitvi06g00056\_t001 |  | \| \| |  |  |  |  |  |  |  |  |  |  |
| 1 | Vitvi06g00058\_t001 |  | \| \| |  |  |  |  |  |  |  |  |  |  |
| 1 | Vitvi06g00059\_t001 |  | Vitvi08g01797\_t001 |  |  |  |  |  |  |  |  |  |  |
| 1 | Vitvi06g00060\_t001 |  | \| \| |  |  |  |  |  |  |  |  |  |  |
| 1 | Vitvi06g00061\_t001 |  | \| \| |  |  |  |  |  |  |  |  |  |  |
| 1 | Vitvi06g00062\_t001 |  | \| \| |  |  |  |  |  |  |  |  |  |  |
| 1 | Vitvi06g00064\_t002 |  | \| \| |  |  |  |  |  |  |  |  |  |  |
| 1 | Vitvi06g04016\_t001 |  | \| \| |  |  |  |  |  |  |  |  |  |  |
| 1 | Vitvi06g04017\_t001 |  | \| \| |  |  |  |  |  |  |  |  |  |  |
| 1 | Vitvi06g04018\_t001 |  | \| \| |  |  |  |  |  |  |  |  |  |  |
| 1 | Vitvi06g04019\_t001 |  | \| \| |  |  |  |  |  |  |  |  |  |  |
| 1 | Vitvi06g04020\_t001 |  | \| \| |  |  |  |  |  |  |  |  |  |  |
| 1 | Vitvi06g00067\_t001 |  | \| \| |  |  |  |  |  |  |  |  |  |  |
| 1 | Vitvi06g04021\_t001 |  | \| \| |  |  |  |  |  |  |  |  |  |  |
| 1 | Vitvi06g04022\_t001 |  | \| \| |  |  |  |  |  |  |  |  |  |  |
| 1 | Vitvi06g00069\_t001 |  | \| \| |  |  |  |  |  |  |  |  |  |  |
| 1 | Vitvi06g04023\_t001 |  | \| \| |  |  |  |  |  |  |  |  |  |  |
| 1 | Vitvi06g00070\_t001 |  | \| \| |  |  |  |  |  |  |  |  |  |  |
| 1 | Vitvi06g00071\_t001 |  | Vitvi08g01794\_t001 |  |  |  |  |  |  |  |  |  |  |
| 1 | Vitvi06g00072\_t001 |  | \| \| |  |  |  |  |  |  |  |  |  |  |
| 2 | Vitvi06g00073\_t001 |  | \| \| |  | Vitvi13g04758\_t002 |  |  |  |  |  |  |  |  |
| 2 | Vitvi06g00074\_t001 |  | \| \| |  | \| \| |  |  |  |  |  |  |  |  |
| 2 | Vitvi06g00075\_t001 |  | \| \| |  | \| \| |  |  |  |  |  |  |  |  |
| 2 | Vitvi06g04024\_t001 |  | \| \| |  | \| \| |  |  |  |  |  |  |  |  |
| 2 | Vitvi06g01588\_t001 |  | \| \| |  | \| \| |  |  |  |  |  |  |  |  |
| 2 | Vitvi06g00076\_t001 |  | \| \| |  | \| \| |  |  |  |  |  |  |  |  |
| 2 | Vitvi06g04025\_t001 |  | \| \| |  | \| \| |  |  |  |  |  |  |  |  |
| 2 | Vitvi06g04026\_t001 |  | \| \| |  | \| \| |  |  |  |  |  |  |  |  |
| 2 | Vitvi06g00077\_t001 |  | \| \| |  | \| \| |  |  |  |  |  |  |  |  |
| 2 | Vitvi06g00078\_t001 |  | \| \| |  | \| \| |  |  |  |  |  |  |  |  |
| 2 | Vitvi06g00079\_t001 |  | \| \| |  | \| \| |  |  |  |  |  |  |  |  |
| 2 | Vitvi06g01590\_t001 |  | \| \| |  | \| \| |  |  |  |  |  |  |  |  |
| 2 | Vitvi06g00080\_t001 |  | \| \| |  | \| \| |  |  |  |  |  |  |  |  |
| 2 | Vitvi06g00083\_t001 |  | \| \| |  | \| \| |  |  |  |  |  |  |  |  |
| 2 | Vitvi06g00084\_t001 |  | \| \| |  | \| \| |  |  |  |  |  |  |  |  |
| 2 | Vitvi06g00085\_t001 |  | \| \| |  | Vitvi13g01842\_t001 |  |  |  |  |  |  |  |  |
| 2 | Vitvi06g00086\_t003 |  | Vitvi08g01782\_t001 |  | Vitvi13g01841\_t001 |  |  |  |  |  |  |  |  |
| 2 | Vitvi06g00087\_t001 |  | \| \| |  | \| \| |  |  |  |  |  |  |  |  |
| 2 | Vitvi06g04027\_t001 |  | \| \| |  | \| \| |  |  |  |  |  |  |  |  |
| 2 | Vitvi06g04028\_t001 |  | Vitvi08g01780\_t001 |  | \| \| |  |  |  |  |  |  |  |  |
| 1 | Vitvi06g00088\_t001 |  |  |  | \| \| |  |  |  |  |  |  |  |  |
| 1 | Vitvi06g00089\_t001 |  |  |  | Vitvi13g01839\_t001 |  |  |  |  |  |  |  |  |
| 1 | Vitvi06g00090\_t001 |  |  |  | \| \| |  |  |  |  |  |  |  |  |
| 1 | Vitvi06g00091\_t003 |  |  |  | \| \| |  |  |  |  |  |  |  |  |
| 1 | Vitvi06g01592\_t001 |  |  |  | \| \| |  |  |  |  |  |  |  |  |
| 1 | Vitvi06g00092\_t001 |  |  |  | \| \| |  |  |  |  |  |  |  |  |
| 1 | Vitvi06g00093\_t001 |  |  |  | \| \| |  |  |  |  |  |  |  |  |
| 1 | Vitvi06g00094\_t001 |  |  |  | \| \| |  |  |  |  |  |  |  |  |
| 1 | Vitvi06g00095\_t001 |  |  |  | \| \| |  |  |  |  |  |  |  |  |
| 1 | Vitvi06g00096\_t001 |  |  |  | \| \| |  |  |  |  |  |  |  |  |
| 1 | Vitvi06g00097\_t001 |  |  |  | \| \| |  |  |  |  |  |  |  |  |
| 1 | Vitvi06g00098\_t001 |  |  |  | \| \| |  |  |  |  |  |  |  |  |
| 1 | Vitvi06g01593\_t001 |  |  |  | \| \| |  |  |  |  |  |  |  |  |
| 1 | Vitvi06g01594\_t001 |  |  |  | \| \| |  |  |  |  |  |  |  |  |
| 1 | Vitvi06g04029\_t001 |  |  |  | \| \| |  |  |  |  |  |  |  |  |
| 1 | Vitvi06g01596\_t001 |  |  |  | \| \| |  |  |  |  |  |  |  |  |
| 1 | Vitvi06g01597\_t001 |  |  |  | \| \| |  |  |  |  |  |  |  |  |
| 1 | Vitvi06g01598\_t001 |  |  |  | \| \| |  |  |  |  |  |  |  |  |
| 1 | Vitvi06g01599\_t001 |  |  |  | \| \| |  |  |  |  |  |  |  |  |
| 1 | Vitvi06g01600\_t001 |  |  |  | \| \| |  |  |  |  |  |  |  |  |
| 1 | Vitvi06g01601\_t001 |  |  |  | \| \| |  |  |  |  |  |  |  |  |
| 1 | Vitvi06g01602\_t001 |  |  |  | \| \| |  |  |  |  |  |  |  |  |
| 1 | Vitvi06g01603\_t001 |  |  |  | \| \| |  |  |  |  |  |  |  |  |
| 1 | Vitvi06g04030\_t001 |  |  |  | \| \| |  |  |  |  |  |  |  |  |
| 1 | Vitvi06g01605\_t001 |  |  |  | \| \| |  |  |  |  |  |  |  |  |
| 1 | Vitvi06g01606\_t001 |  |  |  | Vitvi13g02576\_t001 |  |  |  |  |  |  |  |  |
| 1 | Vitvi06g01607\_t001 |  |  |  | \| \| |  |  |  |  |  |  |  |  |
| 1 | Vitvi06g04031\_t001 |  |  |  | \| \| |  |  |  |  |  |  |  |  |
| 1 | Vitvi06g00100\_t001 |  |  |  | \| \| |  |  |  |  |  |  |  |  |
| 2 | Vitvi06g00102\_t001 |  | Vitvi08g01767\_t001 |  | \| \| |  |  |  |  |  |  |  |  |
| 2 | Vitvi06g00104\_t001 |  | \| \| |  | \| \| |  |  |  |  |  |  |  |  |
| 2 | Vitvi06g00105\_t001 |  | \| \| |  | \| \| |  |  |  |  |  |  |  |  |
| 2 | Vitvi06g00106\_t001 |  | Vitvi08g01762\_t001 |  | \| \| |  |  |  |  |  |  |  |  |
| 2 | Vitvi06g00107\_t001 |  | \| \| |  | \| \| |  |  |  |  |  |  |  |  |
| 2 | Vitvi06g00108\_t002 |  | Vitvi08g01760\_t001 |  | \| \| |  |  |  |  |  |  |  |  |
| 2 | Vitvi06g00109\_t001 |  | \| \| |  | Vitvi13g02574\_t001 |  |  |  |  |  |  |  |  |
| 2 | Vitvi06g01608\_t001 |  | \| \| |  | \| \| |  |  |  |  |  |  |  |  |
| 2 | Vitvi06g04032\_t001 |  | \| \| |  | \| \| |  |  |  |  |  |  |  |  |
| 2 | Vitvi06g00110\_t001 |  | \| \| |  | \| \| |  |  |  |  |  |  |  |  |
| 2 | Vitvi06g00111\_t001 |  | Vitvi08g01750\_t001 |  | \| \| |  |  |  |  |  |  |  |  |
| 2 | Vitvi06g00112\_t001 |  | \| \| |  | \| \| |  |  |  |  |  |  |  |  |
| 2 | Vitvi06g00113\_t001 |  | \| \| |  | Vitvi13g01828\_t001 |  |  |  |  |  |  |  |  |
| 2 | Vitvi06g00114\_t001.3.6037826e |  | \| \| |  | Vitvi13g01827\_t001 |  |  |  |  |  |  |  |  |
| 2 | Vitvi06g00115\_t001 |  | \| \| |  | \| \| |  |  |  |  |  |  |  |  |
| 2 | Vitvi06g00116\_t001 |  | Vitvi08g02349\_t001 |  | Vitvi13g01825\_t001 |  |  |  |  |  |  |  |  |
| 2 | Vitvi06g00117\_t001 |  | \| \| |  | \| \| |  |  |  |  |  |  |  |  |
| 2 | Vitvi06g00118\_t001 |  | \| \| |  | \| \| |  |  |  |  |  |  |  |  |
| 2 | Vitvi06g04033\_t001 |  | \| \| |  | \| \| |  |  |  |  |  |  |  |  |
| 2 | Vitvi06g00119\_t001 |  | Vitvi08g02345\_t001 |  | \| \| |  |  |  |  |  |  |  |  |
| 2 | Vitvi06g04034\_t001 |  | \| \| |  | \| \| |  |  |  |  |  |  |  |  |
| 2 | Vitvi06g00120\_t002 |  | \| \| |  | \| \| |  |  |  |  |  |  |  |  |
| 2 | Vitvi06g00121\_t002 |  | \| \| |  | \| \| |  |  |  |  |  |  |  |  |
| 2 | Vitvi06g04035\_t001 |  | \| \| |  | \| \| |  |  |  |  |  |  |  |  |
| 2 | Vitvi06g04036\_t001 |  | \| \| |  | \| \| |  |  |  |  |  |  |  |  |
| 2 | Vitvi06g01610\_t001 |  | \| \| |  | \| \| |  |  |  |  |  |  |  |  |
| 2 | Vitvi06g00123\_t001 |  | Vitvi08g01732\_t001 |  | Vitvi13g01819\_t001 |  |  |  |  |  |  |  |  |
| 2 | Vitvi06g00124\_t001 |  | \| \| |  | \| \| |  |  |  |  |  |  |  |  |
| 2 | Vitvi06g00125\_t001 |  | Vitvi08g01731\_t001 |  | \| \| |  |  |  |  |  |  |  |  |
| 2 | Vitvi06g00126\_t001 |  | Vitvi08g01730\_t001 |  | \| \| |  |  |  |  |  |  |  |  |
| 2 | Vitvi06g00127\_t001 |  | \| \| |  | \| \| |  |  |  |  |  |  |  |  |
| 2 | Vitvi06g00128\_t001 |  | \| \| |  | \| \| |  |  |  |  |  |  |  |  |
| 2 | Vitvi06g04037\_t001 |  | \| \| |  | \| \| |  |  |  |  |  |  |  |  |
| 2 | Vitvi06g00130\_t001 |  | Vitvi08g01725\_t001 |  | Vitvi13g01816\_t001 |  |  |  |  |  |  |  |  |
| 1 | Vitvi06g04038\_t001 |  | \| \| |  |  |  |  |  |  |  |  |  |  |
| 1 | Vitvi06g00132\_t001 |  | \| \| |  |  |  |  |  |  |  |  |  |  |
| 1 | Vitvi06g04039\_t001 |  | \| \| |  |  |  |  |  |  |  |  |  |  |
| 1 | Vitvi06g00134\_t001 |  | \| \| |  |  |  |  |  |  |  |  |  |  |
| 1 | Vitvi06g01611\_t001 |  | \| \| |  |  |  |  |  |  |  |  |  |  |
| 1 | Vitvi06g00136\_t001 |  | \| \| |  |  |  |  |  |  |  |  |  |  |
| 1 | Vitvi06g00137\_t001 |  | \| \| |  |  |  |  |  |  |  |  |  |  |
| 1 | Vitvi06g01612\_t001 |  | \| \| |  |  |  |  |  |  |  |  |  |  |
| 1 | Vitvi06g00138\_t001 |  | \| \| |  |  |  |  |  |  |  |  |  |  |
| 1 | Vitvi06g01613\_t001 |  | \| \| |  |  |  |  |  |  |  |  |  |  |
| 1 | Vitvi06g04040\_t001 |  | \| \| |  |  |  |  |  |  |  |  |  |  |
| 1 | Vitvi06g04041\_t001 |  | \| \| |  |  |  |  |  |  |  |  |  |  |
| 1 | Vitvi06g00139\_t001 |  | \| \| |  |  |  |  |  |  |  |  |  |  |
| 1 | Vitvi06g00140\_t001 |  | \| \| |  |  |  |  |  |  |  |  |  |  |
| 1 | Vitvi06g01615\_t001 |  | Vitvi08g02342\_t001 |  |  |  |  |  |  |  |  |  |  |
| 2 | Vitvi06g00141\_t001 |  | \| \| |  | Vitvi14g04492\_t001 |  |  |  |  |  |  |  |  |
| 2 | Vitvi06g04042\_t001 |  | \| \| |  | \| \| |  |  |  |  |  |  |  |  |
| 2 | Vitvi06g04043\_t001 |  | \| \| |  | \| \| |  |  |  |  |  |  |  |  |
| 2 | Vitvi06g04044\_t001 |  | \| \| |  | \| \| |  |  |  |  |  |  |  |  |
| 2 | Vitvi06g04045\_t001 |  | \| \| |  | \| \| |  |  |  |  |  |  |  |  |
| 2 | Vitvi06g00143\_t001 |  | \| \| |  | Vitvi14g04491\_t001 |  |  |  |  |  |  |  |  |
| 2 | Vitvi06g00149\_t001 |  | \| \| |  | \| \| |  |  |  |  |  |  |  |  |
| 2 | Vitvi06g04046\_t001 |  | \| \| |  | \| \| |  |  |  |  |  |  |  |  |
| 2 | Vitvi06g00150\_t001 |  | \| \| |  | \| \| |  |  |  |  |  |  |  |  |
| 2 | Vitvi06g00153\_t001 |  | \| \| |  | \| \| |  |  |  |  |  |  |  |  |
| 2 | Vitvi06g00155\_t001 |  | \| \| |  | Vitvi14g04489\_t001 |  |  |  |  |  |  |  |  |
| 2 | Vitvi06g00156\_t001 |  | \| \| |  | \| \| |  |  |  |  |  |  |  |  |
| 2 | Vitvi06g00158\_t001 |  | \| \| |  | \| \| |  |  |  |  |  |  |  |  |
| 2 | Vitvi06g00159\_t001 |  | \| \| |  | \| \| |  |  |  |  |  |  |  |  |
| 2 | Vitvi06g00160\_t001 |  | \| \| |  | \| \| |  |  |  |  |  |  |  |  |
| 2 | Vitvi06g00161\_t001 |  | \| \| |  | \| \| |  |  |  |  |  |  |  |  |
| 2 | Vitvi06g00162\_t001 |  | Vitvi08g01708\_t001 |  | Vitvi13g01773\_t001 |  |  |  |  |  |  |  |  |
| 1 | Vitvi06g04047\_t001 |  |  |  | \| \| |  |  |  |  |  |  |  |  |
| 1 | Vitvi06g00163\_t001 |  |  |  | \| \| |  |  |  |  |  |  |  |  |
| 1 | Vitvi06g01616\_t001 |  |  |  | \| \| |  |  |  |  |  |  |  |  |
| 1 | Vitvi06g01617\_t001 |  |  |  | \| \| |  |  |  |  |  |  |  |  |
| 1 | Vitvi06g04048\_t001 |  |  |  | \| \| |  |  |  |  |  |  |  |  |
| 1 | Vitvi06g04049\_t001 |  |  |  | \| \| |  |  |  |  |  |  |  |  |
| 1 | Vitvi06g01618\_t001 |  |  |  | \| \| |  |  |  |  |  |  |  |  |
| 1 | Vitvi06g00165\_t001 |  |  |  | \| \| |  |  |  |  |  |  |  |  |
| 1 | Vitvi06g04050\_t001 |  |  |  | \| \| |  |  |  |  |  |  |  |  |
| 1 | Vitvi06g00166\_t001 |  |  |  | \| \| |  |  |  |  |  |  |  |  |
| 1 | Vitvi06g04051\_t001 |  |  |  | \| \| |  |  |  |  |  |  |  |  |
| 1 | Vitvi06g00167\_t001 |  |  |  | \| \| |  |  |  |  |  |  |  |  |
| 1 | Vitvi06g00169\_t001 |  |  |  | \| \| |  |  |  |  |  |  |  |  |
| 1 | Vitvi06g00170\_t001 |  |  |  | \| \| |  |  |  |  |  |  |  |  |
| 1 | Vitvi06g01620\_t001 |  |  |  | \| \| |  |  |  |  |  |  |  |  |
| 1 | Vitvi06g00172\_t001 |  |  |  | \| \| |  |  |  |  |  |  |  |  |
| 1 | Vitvi06g00173\_t001 |  |  |  | \| \| |  |  |  |  |  |  |  |  |
| 1 | Vitvi06g04052\_t001 |  |  |  | Vitvi14g04481\_t001 |  |  |  |  |  |  |  |  |
| 1 | Vitvi06g00177\_t001 |  |  |  | \| \| |  |  |  |  |  |  |  |  |
| 1 | Vitvi06g04053\_t001 |  |  |  | \| \| |  |  |  |  |  |  |  |  |
| 1 | Vitvi06g00179\_t001 |  |  |  | \| \| |  |  |  |  |  |  |  |  |
| 1 | Vitvi06g00180\_t001 |  |  |  | \| \| |  |  |  |  |  |  |  |  |
| 2 | Vitvi06g00181\_t001 |  | Vitvi08g01852\_t001 |  | Vitvi13g01764\_t001 |  |  |  |  |  |  |  |  |
| 2 | Vitvi06g00182\_t001 |  | \| \| |  | Vitvi13g01760\_t001 |  |  |  |  |  |  |  |  |
| 2 | Vitvi06g04054\_t001 |  | \| \| |  | \| \| |  |  |  |  |  |  |  |  |
| 2 | Vitvi06g04055\_t001 |  | \| \| |  | \| \| |  |  |  |  |  |  |  |  |
| 2 | Vitvi06g00184\_t001 |  | \| \| |  | \| \| |  |  |  |  |  |  |  |  |
| 2 | Vitvi06g00185\_t001 |  | \| \| |  | \| \| |  |  |  |  |  |  |  |  |
| 2 | Vitvi06g00186\_t001 |  | \| \| |  | Vitvi13g01756\_t001 |  |  |  |  |  |  |  |  |
| 2 | Vitvi06g00187\_t001 |  | Vitvi08g01861\_t001 |  | \| \| |  |  |  |  |  |  |  |  |
| 2 | Vitvi06g00188\_t001 |  | \| \| |  | \| \| |  |  |  |  |  |  |  |  |
| 2 | Vitvi06g00189\_t003 |  | Vitvi08g01864\_t001 |  | Vitvi13g01755\_t001 |  |  |  |  |  |  |  |  |
| 2 | Vitvi06g00191\_t002 |  | Vitvi08g01866\_t002 |  | \| \| |  |  |  |  |  |  |  |  |
| 2 | Vitvi06g04056\_t001 |  | Vitvi08g02381\_t001 |  | Vitvi13g02516\_t001 |  |  |  |  |  |  |  |  |
| 1 | Vitvi06g04057\_t001 |  | Vitvi08g02382\_t001 |  |  |  |  |  |  |  |  |  |  |
| 1 | Vitvi06g04058\_t001 |  | \| \| |  |  |  |  |  |  |  |  |  |  |
| 1 | Vitvi06g04059\_t001 |  | \| \| |  |  |  |  |  |  |  |  |  |  |
| 1 | Vitvi06g00192\_t001 |  | \| \| |  |  |  |  |  |  |  |  |  |  |
| 1 | Vitvi06g04060\_t001 |  | \| \| |  |  |  |  |  |  |  |  |  |  |
| 1 | Vitvi06g00193\_t001 |  | \| \| |  |  |  |  |  |  |  |  |  |  |
| 2 | Vitvi06g00195\_t001 |  | Vitvi08g01869\_t001 |  | Vitvi13g01748\_t001 |  |  |  |  |  |  |  |  |
| 1 | Vitvi06g00198\_t001 |  |  |  | \| \| |  |  |  |  |  |  |  |  |
| 1 | Vitvi06g00199\_t003 |  |  |  | \| \| |  |  |  |  |  |  |  |  |
| 1 | Vitvi06g00200\_t001 |  |  |  | \| \| |  |  |  |  |  |  |  |  |
| 1 | Vitvi06g04061\_t001 |  |  |  | \| \| |  |  |  |  |  |  |  |  |
| 1 | Vitvi06g00201\_t002 |  |  |  | Vitvi13g01745\_t001 |  |  |  |  |  |  |  |  |
| 1 | Vitvi06g00202\_t001 |  |  |  | \| \| |  |  |  |  |  |  |  |  |
| 1 | Vitvi06g04062\_t001 |  |  |  | Vitvi13g01744\_t001 |  |  |  |  |  |  |  |  |
| 1 | Vitvi06g01622\_t001 |  |  |  | \| \| |  |  |  |  |  |  |  |  |
| 1 | Vitvi06g01625\_t001 |  |  |  | \| \| |  |  |  |  |  |  |  |  |
| 1 | Vitvi06g01626\_t001 |  |  |  | \| \| |  |  |  |  |  |  |  |  |
| 1 | Vitvi06g04063\_t001 |  |  |  | \| \| |  |  |  |  |  |  |  |  |
| 1 | Vitvi06g04064\_t001 |  |  |  | \| \| |  |  |  |  |  |  |  |  |
| 1 | Vitvi06g01628\_t001 |  |  |  | \| \| |  |  |  |  |  |  |  |  |
| 1 | Vitvi06g01629\_t001 |  |  |  | \| \| |  |  |  |  |  |  |  |  |
| 1 | Vitvi06g04065\_t001 |  |  |  | \| \| |  |  |  |  |  |  |  |  |
| 1 | Vitvi06g04066\_t001 |  |  |  | \| \| |  |  |  |  |  |  |  |  |
| 1 | Vitvi06g04067\_t001 |  |  |  | \| \| |  |  |  |  |  |  |  |  |
| 1 | Vitvi06g00205\_t001 |  |  |  | \| \| |  |  |  |  |  |  |  |  |
| 1 | Vitvi06g00206\_t001 |  |  |  | \| \| |  |  |  |  |  |  |  |  |
| 1 | Vitvi06g00207\_t001 |  |  |  | \| \| |  |  |  |  |  |  |  |  |
| 1 | Vitvi06g00209\_t001 |  |  |  | \| \| |  |  |  |  |  |  |  |  |
| 1 | Vitvi06g04068\_t001 |  |  |  | \| \| |  |  |  |  |  |  |  |  |
| 1 | Vitvi06g00210\_t001 |  |  |  | \| \| |  |  |  |  |  |  |  |  |
| 1 | Vitvi06g00211\_t001 |  |  |  | \| \| |  |  |  |  |  |  |  |  |
| 1 | Vitvi06g01631\_t001 |  |  |  | \| \| |  |  |  |  |  |  |  |  |
| 1 | Vitvi06g00212\_t001 |  |  |  | \| \| |  |  |  |  |  |  |  |  |
| 1 | Vitvi06g00213\_t002 |  |  |  | \| \| |  |  |  |  |  |  |  |  |
| 1 | Vitvi06g00214\_t001 |  |  |  | Vitvi13g01737\_t001 |  |  |  |  |  |  |  |  |
| 1 | Vitvi06g00215\_t001 |  |  |  | \| \| |  |  |  |  |  |  |  |  |
| 1 | Vitvi06g04069\_t001 |  |  |  | \| \| |  |  |  |  |  |  |  |  |
| 1 | Vitvi06g00216\_t001 |  |  |  | Vitvi13g01734\_t001 |  |  |  |  |  |  |  |  |
| 1 | Vitvi06g00217\_t001 |  |  |  | \| \| |  |  |  |  |  |  |  |  |
| 1 | Vitvi06g00218\_t003 |  |  |  | Vitvi13g01732\_t001 |  |  |  |  |  |  |  |  |
| 1 | Vitvi06g04070\_t001 |  |  |  | \| \| |  |  |  |  |  |  |  |  |
| 1 | Vitvi06g00220\_t001 |  |  |  | \| \| |  |  |  |  |  |  |  |  |
| 1 | Vitvi06g01633\_t001 |  |  |  | \| \| |  |  |  |  |  |  |  |  |
| 1 | Vitvi06g00222\_t001 |  |  |  | Vitvi13g04751\_t001 |  |  |  |  |  |  |  |  |
| 1 | Vitvi06g00223\_t001 |  |  |  | \| \| |  |  |  |  |  |  |  |  |
| 1 | Vitvi06g01635\_t001 |  |  |  | \| \| |  |  |  |  |  |  |  |  |
| 1 | Vitvi06g01636\_t001 |  |  |  | \| \| |  |  |  |  |  |  |  |  |
| 1 | Vitvi06g04071\_t001 |  |  |  | \| \| |  |  |  |  |  |  |  |  |
| 1 | Vitvi06g00224\_t001 |  |  |  | Vitvi13g01729\_t002 |  |  |  |  |  |  |  |  |
| 1 | Vitvi06g00225\_t001 |  |  |  | \| \| |  |  |  |  |  |  |  |  |
| 1 | Vitvi06g04072\_t001 |  |  |  | \| \| |  |  |  |  |  |  |  |  |
| 1 | Vitvi06g00226\_t001 |  |  |  | \| \| |  |  |  |  |  |  |  |  |
| 1 | Vitvi06g00227\_t001 |  |  |  | \| \| |  |  |  |  |  |  |  |  |
| 1 | Vitvi06g04073\_t001 |  |  |  | \| \| |  |  |  |  |  |  |  |  |
| 1 | Vitvi06g00229\_t001 |  |  |  | \| \| |  |  |  |  |  |  |  |  |
| 1 | Vitvi06g00230\_t001 |  |  |  | \| \| |  |  |  |  |  |  |  |  |
| 1 | Vitvi06g01637\_t001 |  |  |  | Vitvi13g02488\_t001 |  |  |  |  |  |  |  |  |
| 0 | Vitvi06g04074\_t001 |  |  |  |  |  |  |  |  |  |  |  |  |
| 0 | Vitvi06g04075\_t001 |  |  |  |  |  |  |  |  |  |  |  |  |
| 0 | Vitvi06g04076\_t001 |  |  |  |  |  |  |  |  |  |  |  |  |
| 0 | Vitvi06g04077\_t001 |  |  |  |  |  |  |  |  |  |  |  |  |
| 0 | Vitvi06g04078\_t001 |  |  |  |  |  |  |  |  |  |  |  |  |
| 0 | Vitvi06g01639\_t001 |  |  |  |  |  |  |  |  |  |  |  |  |
| 0 | Vitvi06g01640\_t001 |  |  |  |  |  |  |  |  |  |  |  |  |
| 0 | Vitvi06g04079\_t001 |  |  |  |  |  |  |  |  |  |  |  |  |
| 0 | Vitvi06g01641\_t001 |  |  |  |  |  |  |  |  |  |  |  |  |
| 0 | Vitvi06g04080\_t001 |  |  |  |  |  |  |  |  |  |  |  |  |
| 0 | Vitvi06g00233\_t001 |  |  |  |  |  |  |  |  |  |  |  |  |
| 0 | Vitvi06g01644\_t001 |  |  |  |  |  |  |  |  |  |  |  |  |
| 0 | Vitvi06g00234\_t001 |  |  |  |  |  |  |  |  |  |  |  |  |
| 0 | Vitvi06g00235\_t001 |  |  |  |  |  |  |  |  |  |  |  |  |
| 0 | Vitvi06g00236\_t001 |  |  |  |  |  |  |  |  |  |  |  |  |
| 0 | Vitvi06g00237\_t001 |  |  |  |  |  |  |  |  |  |  |  |  |
| 0 | Vitvi06g04081\_t001 |  |  |  |  |  |  |  |  |  |  |  |  |
| 0 | Vitvi06g00238\_t001 |  |  |  |  |  |  |  |  |  |  |  |  |
| 1 | Vitvi06g01646\_t001 |  | Vitvi08g01011\_t001 |  |  |  |  |  |  |  |  |  |  |
| 2 | Vitvi06g00241\_t003 |  | \| \| |  | Vitvi13g00640\_t001 |  |  |  |  |  |  |  |  |
| 2 | Vitvi06g00242\_t001 |  | Vitvi08g01013\_t001 |  | Vitvi13g00638\_t001 |  |  |  |  |  |  |  |  |
| 2 | Vitvi06g00243\_t001 |  | \| \| |  | Vitvi13g00637\_t001.1.6037826b |  |  |  |  |  |  |  |  |
| 2 | Vitvi06g00244\_t001 |  | \| \| |  | \| \| |  |  |  |  |  |  |  |  |
| 2 | Vitvi06g00245\_t001 |  | Vitvi08g02140\_t003 |  | \| \| |  |  |  |  |  |  |  |  |
| 2 | Vitvi06g00246\_t001 |  | Vitvi08g01015\_t001 |  | Vitvi13g00632\_t001 |  |  |  |  |  |  |  |  |
| 2 | Vitvi06g01647\_t001 |  | \| \| |  | \| \| |  |  |  |  |  |  |  |  |
| 2 | Vitvi06g01648\_t001 |  | \| \| |  | \| \| |  |  |  |  |  |  |  |  |
| 2 | Vitvi06g04082\_t001 |  | \| \| |  | \| \| |  |  |  |  |  |  |  |  |
| 2 | Vitvi06g01649\_t001 |  | \| \| |  | \| \| |  |  |  |  |  |  |  |  |
| 2 | Vitvi06g04083\_t001 |  | \| \| |  | \| \| |  |  |  |  |  |  |  |  |
| 2 | Vitvi06g04084\_t001 |  | \| \| |  | \| \| |  |  |  |  |  |  |  |  |
| 2 | Vitvi06g04085\_t001 |  | \| \| |  | \| \| |  |  |  |  |  |  |  |  |
| 2 | Vitvi06g00248\_t001 |  | \| \| |  | \| \| |  |  |  |  |  |  |  |  |
| 2 | Vitvi06g01651\_t001 |  | \| \| |  | \| \| |  |  |  |  |  |  |  |  |
| 2 | Vitvi06g04086\_t001 |  | \| \| |  | \| \| |  |  |  |  |  |  |  |  |
| 2 | Vitvi06g04087\_t001 |  | \| \| |  | \| \| |  |  |  |  |  |  |  |  |
| 2 | Vitvi06g04088\_t001 |  | \| \| |  | \| \| |  |  |  |  |  |  |  |  |
| 2 | Vitvi06g04089\_t001 |  | \| \| |  | \| \| |  |  |  |  |  |  |  |  |
| 2 | Vitvi06g00250\_t001 |  | \| \| |  | \| \| |  |  |  |  |  |  |  |  |
| 2 | Vitvi06g00251\_t001 |  | \| \| |  | \| \| |  |  |  |  |  |  |  |  |
| 2 | Vitvi06g04090\_t001 |  | \| \| |  | \| \| |  |  |  |  |  |  |  |  |
| 2 | Vitvi06g04091\_t001 |  | \| \| |  | Vitvi13g00622\_t001 |  |  |  |  |  |  |  |  |
| 2 | Vitvi06g00258\_t001 |  | \| \| |  | Vitvi13g00620\_t001 |  |  |  |  |  |  |  |  |
| 2 | Vitvi06g04092\_t001 |  | \| \| |  | \| \| |  |  |  |  |  |  |  |  |
| 2 | Vitvi06g00259\_t001 |  | \| \| |  | \| \| |  |  |  |  |  |  |  |  |
| 2 | Vitvi06g04093\_t001 |  | \| \| |  | \| \| |  |  |  |  |  |  |  |  |
| 2 | Vitvi06g00261\_t001 |  | \| \| |  | \| \| |  |  |  |  |  |  |  |  |
| 2 | Vitvi06g04094\_t001 |  | \| \| |  | \| \| |  |  |  |  |  |  |  |  |
| 2 | Vitvi06g00262\_t001 |  | Vitvi08g01028\_t001 |  | \| \| |  |  |  |  |  |  |  |  |
| 2 | Vitvi06g00263\_t001 |  | Vitvi08g01030\_t001 |  | \| \| |  |  |  |  |  |  |  |  |
| 2 | Vitvi06g00266\_t001 |  | \| \| |  | \| \| |  |  |  |  |  |  |  |  |
| 2 | Vitvi06g00267\_t001 |  | \| \| |  | \| \| |  |  |  |  |  |  |  |  |
| 2 | Vitvi06g00268\_t001 |  | \| \| |  | \| \| |  |  |  |  |  |  |  |  |
| 2 | Vitvi06g00269\_t001 |  | \| \| |  | Vitvi13g00615\_t001 |  |  |  |  |  |  |  |  |
| 2 | Vitvi06g04095\_t001 |  | \| \| |  | Vitvi13g00614\_t001 |  |  |  |  |  |  |  |  |
| 2 | Vitvi06g00272\_t002 |  | Vitvi08g01033\_t001 |  | Vitvi13g02058\_t001 |  |  |  |  |  |  |  |  |
| 2 | Vitvi06g04096\_t001 |  | \| \| |  | \| \| |  |  |  |  |  |  |  |  |
| 2 | Vitvi06g04097\_t001 |  | \| \| |  | \| \| |  |  |  |  |  |  |  |  |
| 2 | Vitvi06g00274\_t004 |  | \| \| |  | \| \| |  |  |  |  |  |  |  |  |
| 2 | Vitvi06g04098\_t001 |  | \| \| |  | \| \| |  |  |  |  |  |  |  |  |
| 2 | Vitvi06g00275\_t001 |  | Vitvi08g01035\_t001 |  | Vitvi13g00610\_t001 |  |  |  |  |  |  |  |  |
| 2 | Vitvi06g01656\_t001 |  | \| \| |  | \| \| |  |  |  |  |  |  |  |  |
| 2 | Vitvi06g00276\_t001 |  | \| \| |  | Vitvi13g00609\_t001 |  |  |  |  |  |  |  |  |
| 2 | Vitvi06g04099\_t001 |  | \| \| |  | \| \| |  |  |  |  |  |  |  |  |
| 2 | Vitvi06g00277\_t001 |  | \| \| |  | \| \| |  |  |  |  |  |  |  |  |
| 2 | Vitvi06g00278\_t001 |  | \| \| |  | \| \| |  |  |  |  |  |  |  |  |
| 2 | Vitvi06g04100\_t001 |  | \| \| |  | \| \| |  |  |  |  |  |  |  |  |
| 2 | Vitvi06g04101\_t001 |  | \| \| |  | \| \| |  |  |  |  |  |  |  |  |
| 2 | Vitvi06g00279\_t001 |  | Vitvi08g01036\_t001 |  | \| \| |  |  |  |  |  |  |  |  |
| 2 | Vitvi06g00280\_t001 |  | Vitvi08g01037\_t001 |  | Vitvi13g00606\_t001 |  |  |  |  |  |  |  |  |
| 2 | Vitvi06g00281\_t001 |  | Vitvi08g01038\_t001 |  | Vitvi13g00605\_t001 |  |  |  |  |  |  |  |  |
| 2 | Vitvi06g01658\_t001 |  | \| \| |  | \| \| |  |  |  |  |  |  |  |  |
| 2 | Vitvi06g00282\_t001 |  | \| \| |  | \| \| |  |  |  |  |  |  |  |  |
| 2 | Vitvi06g00283\_t001 |  | \| \| |  | \| \| |  |  |  |  |  |  |  |  |
| 2 | Vitvi06g00284\_t001 |  | \| \| |  | Vitvi13g00602\_t001 |  |  |  |  |  |  |  |  |
| 2 | Vitvi06g00285\_t001 |  | \| \| |  | \| \| |  |  |  |  |  |  |  |  |
| 2 | Vitvi06g00286\_t001 |  | \| \| |  | \| \| |  |  |  |  |  |  |  |  |
| 2 | Vitvi06g00287\_t001 |  | \| \| |  | \| \| |  |  |  |  |  |  |  |  |
| 2 | Vitvi06g00288\_t001 |  | \| \| |  | \| \| |  |  |  |  |  |  |  |  |
| 2 | Vitvi06g00289\_t001 |  | \| \| |  | \| \| |  |  |  |  |  |  |  |  |
| 2 | Vitvi06g00290\_t001 |  | \| \| |  | \| \| |  |  |  |  |  |  |  |  |
| 2 | Vitvi06g04102\_t001 |  | \| \| |  | \| \| |  |  |  |  |  |  |  |  |
| 2 | Vitvi06g00292\_t001 |  | \| \| |  | \| \| |  |  |  |  |  |  |  |  |
| 2 | Vitvi06g00293\_t001 |  | \| \| |  | Vitvi13g00597\_t002 |  |  |  |  |  |  |  |  |
| 2 | Vitvi06g00294\_t001 |  | Vitvi08g01042\_t001 |  | \| \| |  |  |  |  |  |  |  |  |
| 2 | Vitvi06g00295\_t001 |  | \| \| |  | \| \| |  |  |  |  |  |  |  |  |
| 2 | Vitvi06g04103\_t001 |  | \| \| |  | \| \| |  |  |  |  |  |  |  |  |
| 2 | Vitvi06g00296\_t002 |  | \| \| |  | \| \| |  |  |  |  |  |  |  |  |
| 2 | Vitvi06g04104\_t001 |  | \| \| |  | \| \| |  |  |  |  |  |  |  |  |
| 2 | Vitvi06g00298\_t001 |  | \| \| |  | \| \| |  |  |  |  |  |  |  |  |
| 2 | Vitvi06g00299\_t001 |  | \| \| |  | \| \| |  |  |  |  |  |  |  |  |
| 2 | Vitvi06g00300\_t001 |  | \| \| |  | \| \| |  |  |  |  |  |  |  |  |
| 2 | Vitvi06g00301\_t001 |  | \| \| |  | \| \| |  |  |  |  |  |  |  |  |
| 2 | Vitvi06g01659\_t001 |  | \| \| |  | \| \| |  |  |  |  |  |  |  |  |
| 2 | Vitvi06g00302\_t001 |  | Vitvi08g01044\_t001 |  | \| \| |  |  |  |  |  |  |  |  |
| 2 | Vitvi06g00303\_t001 |  | Vitvi08g02156\_t001 |  | \| \| |  |  |  |  |  |  |  |  |
| 2 | Vitvi06g00304\_t001 |  | Vitvi08g01050\_t003 |  | \| \| |  |  |  |  |  |  |  |  |
| 2 | Vitvi06g00305\_t001 |  | Vitvi08g01052\_t001 |  | \| \| |  |  |  |  |  |  |  |  |
| 2 | Vitvi06g00306\_t001 |  | Vitvi08g01053\_t001 |  | \| \| |  |  |  |  |  |  |  |  |
| 2 | Vitvi06g04105\_t001 |  | \| \| |  | \| \| |  |  |  |  |  |  |  |  |
| 2 | Vitvi06g01661\_t001 |  | \| \| |  | Vitvi13g00583\_t001 |  |  |  |  |  |  |  |  |
| 2 | Vitvi06g00308\_t001 |  | Vitvi08g01055\_t001 |  | Vitvi13g00578\_t001 |  |  |  |  |  |  |  |  |
| 2 | Vitvi06g00309\_t001 |  | \| \| |  | \| \| |  |  |  |  |  |  |  |  |
| 2 | Vitvi06g00310\_t001 |  | \| \| |  | \| \| |  |  |  |  |  |  |  |  |
| 2 | Vitvi06g00311\_t001 |  | \| \| |  | \| \| |  |  |  |  |  |  |  |  |
| 2 | Vitvi06g00312\_t001 |  | \| \| |  | \| \| |  |  |  |  |  |  |  |  |
| 2 | Vitvi06g00313\_t001 |  | \| \| |  | \| \| |  |  |  |  |  |  |  |  |
| 2 | Vitvi06g01662\_t001 |  | Vitvi08g02161\_t003 |  | \| \| |  |  |  |  |  |  |  |  |
| 2 | Vitvi06g00314\_t001 |  | \| \| |  | \| \| |  |  |  |  |  |  |  |  |
| 2 | Vitvi06g00315\_t003 |  | \| \| |  | \| \| |  |  |  |  |  |  |  |  |
| 2 | Vitvi06g04106\_t001 |  | \| \| |  | \| \| |  |  |  |  |  |  |  |  |
| 2 | Vitvi06g00316\_t002 |  | \| \| |  | \| \| |  |  |  |  |  |  |  |  |
| 2 | Vitvi06g00318\_t001 |  | \| \| |  | \| \| |  |  |  |  |  |  |  |  |
| 2 | Vitvi06g04107\_t001 |  | Vitvi08g01069\_t001 |  | Vitvi13g02054\_t001 |  |  |  |  |  |  |  |  |
| 2 | Vitvi06g01664\_t002 |  | \| \| |  | \| \| |  |  |  |  |  |  |  |  |
| 2 | Vitvi06g00320\_t001 |  | \| \| |  | \| \| |  |  |  |  |  |  |  |  |
| 2 | Vitvi06g04108\_t001 |  | \| \| |  | \| \| |  |  |  |  |  |  |  |  |
| 2 | Vitvi06g04109\_t003 |  | \| \| |  | \| \| |  |  |  |  |  |  |  |  |
| 2 | Vitvi06g00322\_t001 |  | \| \| |  | \| \| |  |  |  |  |  |  |  |  |
| 2 | Vitvi06g00323\_t001 |  | \| \| |  | \| \| |  |  |  |  |  |  |  |  |
| 2 | Vitvi06g00325\_t001 |  | \| \| |  | \| \| |  |  |  |  |  |  |  |  |
| 2 | Vitvi06g00327\_t001 |  | \| \| |  | \| \| |  |  |  |  |  |  |  |  |
| 2 | Vitvi06g00328\_t001 |  | \| \| |  | \| \| |  |  |  |  |  |  |  |  |
| 2 | Vitvi06g00329\_t001 |  | \| \| |  | \| \| |  |  |  |  |  |  |  |  |
| 2 | Vitvi06g00330\_t001 |  | \| \| |  | \| \| |  |  |  |  |  |  |  |  |
| 2 | Vitvi06g04110\_t001 |  | \| \| |  | \| \| |  |  |  |  |  |  |  |  |
| 2 | Vitvi06g00331\_t001 |  | Vitvi08g01073\_t001 |  | \| \| |  |  |  |  |  |  |  |  |
| 2 | Vitvi06g00334\_t001 |  | \| \| |  | \| \| |  |  |  |  |  |  |  |  |
| 2 | Vitvi06g00335\_t001 |  | \| \| |  | \| \| |  |  |  |  |  |  |  |  |
| 2 | Vitvi06g00336\_t001 |  | \| \| |  | Vitvi13g00543\_t001 |  |  |  |  |  |  |  |  |
| 2 | Vitvi06g00337\_t001 |  | \| \| |  | \| \| |  |  |  |  |  |  |  |  |
| 2 | Vitvi06g00338\_t001 |  | \| \| |  | \| \| |  |  |  |  |  |  |  |  |
| 2 | Vitvi06g00339\_t001 |  | \| \| |  | \| \| |  |  |  |  |  |  |  |  |
| 2 | Vitvi06g04111\_t001 |  | \| \| |  | \| \| |  |  |  |  |  |  |  |  |
| 2 | Vitvi06g00340\_t001 |  | \| \| |  | \| \| |  |  |  |  |  |  |  |  |
| 2 | Vitvi06g00341\_t001 |  | \| \| |  | \| \| |  |  |  |  |  |  |  |  |
| 2 | Vitvi06g00342\_t001 |  | \| \| |  | \| \| |  |  |  |  |  |  |  |  |
| 2 | Vitvi06g01665\_t004 |  | Vitvi08g01075\_t003 |  | \| \| |  |  |  |  |  |  |  |  |
| 2 | Vitvi06g04112\_t001 |  | Vitvi08g01076\_t001 |  | Vitvi13g04156\_t001 |  |  |  |  |  |  |  |  |
| 1 | Vitvi06g00345\_t001 |  |  |  | \| \| |  |  |  |  |  |  |  |  |
| 1 | Vitvi06g00346\_t001 |  |  |  | \| \| |  |  |  |  |  |  |  |  |
| 1 | Vitvi06g00347\_t001 |  |  |  | \| \| |  |  |  |  |  |  |  |  |
| 1 | Vitvi06g04113\_t001 |  |  |  | \| \| |  |  |  |  |  |  |  |  |
| 1 | Vitvi06g00348\_t001 |  |  |  | \| \| |  |  |  |  |  |  |  |  |
| 1 | Vitvi06g00349\_t001 |  |  |  | \| \| |  |  |  |  |  |  |  |  |
| 1 | Vitvi06g00350\_t001 |  |  |  | Vitvi13g00538\_t001 |  |  |  |  |  |  |  |  |
| 2 | Vitvi06g00351\_t001 |  | Vitvi08g01154\_t001 |  | Vitvi13g00535\_t001 |  |  |  |  |  |  |  |  |
| 2 | Vitvi06g00352\_t001 |  | \| \| |  | Vitvi13g04154\_t001 |  |  |  |  |  |  |  |  |
| 2 | Vitvi06g04114\_t001 |  | \| \| |  | \| \| |  |  |  |  |  |  |  |  |
| 2 | Vitvi06g00354\_t001 |  | \| \| |  | \| \| |  |  |  |  |  |  |  |  |
| 2 | Vitvi06g01667\_t001 |  | \| \| |  | \| \| |  |  |  |  |  |  |  |  |
| 2 | Vitvi06g00356\_t001 |  | \| \| |  | \| \| |  |  |  |  |  |  |  |  |
| 2 | Vitvi06g00358\_t003 |  | \| \| |  | \| \| |  |  |  |  |  |  |  |  |
| 2 | Vitvi06g00359\_t002 |  | \| \| |  | \| \| |  |  |  |  |  |  |  |  |
| 2 | Vitvi06g00360\_t001 |  | Vitvi08g01146\_t001 |  | Vitvi13g00529\_t001 |  |  |  |  |  |  |  |  |
| 2 | Vitvi06g00362\_t001 |  | \| \| |  | \| \| |  |  |  |  |  |  |  |  |
| 2 | Vitvi06g04115\_t001 |  | \| \| |  | \| \| |  |  |  |  |  |  |  |  |
| 2 | Vitvi06g04116\_t001 |  | \| \| |  | \| \| |  |  |  |  |  |  |  |  |
| 2 | Vitvi06g04117\_t001 |  | \| \| |  | \| \| |  |  |  |  |  |  |  |  |
| 2 | Vitvi06g04118\_t001 |  | \| \| |  | \| \| |  |  |  |  |  |  |  |  |
| 2 | Vitvi06g04119\_t001 |  | \| \| |  | \| \| |  |  |  |  |  |  |  |  |
| 2 | Vitvi06g04120\_t001 |  | \| \| |  | \| \| |  |  |  |  |  |  |  |  |
| 2 | Vitvi06g04121\_t001 |  | \| \| |  | \| \| |  |  |  |  |  |  |  |  |
| 2 | Vitvi06g04122\_t001 |  | \| \| |  | \| \| |  |  |  |  |  |  |  |  |
| 2 | Vitvi06g00365\_t001 |  | \| \| |  | \| \| |  |  |  |  |  |  |  |  |
| 2 | Vitvi06g00366\_t001 |  | \| \| |  | Vitvi13g00523\_t001 |  |  |  |  |  |  |  |  |
| 2 | Vitvi06g00367\_t001.1.6037826e |  | \| \| |  | \| \| |  |  |  |  |  |  |  |  |
| 3 | Vitvi06g00368\_t001 |  | Vitvi08g01139\_t001 |  | \| \| |  | Vitvi13g00203\_t004 |  |  |  |  |  |  |
| 3 | Vitvi06g00369\_t001 |  | Vitvi08g01133\_t002 |  | \| \| |  | Vitvi13g00205\_t001 |  |  |  |  |  |  |
| 3 | Vitvi06g00370\_t001 |  | \| \| |  | \| \| |  | \| \| |  |  |  |  |  |  |
| 3 | Vitvi06g00371\_t001 |  | \| \| |  | \| \| |  | \| \| |  |  |  |  |  |  |
| 3 | Vitvi06g00372\_t001 |  | Vitvi08g01129\_t001 |  | \| \| |  | Vitvi13g00208\_t001 |  |  |  |  |  |  |
| 3 | Vitvi06g04123\_t001 |  | \| \| |  | \| \| |  | \| \| |  |  |  |  |  |  |
| 3 | Vitvi06g00374\_t001 |  | \| \| |  | \| \| |  | \| \| |  |  |  |  |  |  |
| 3 | Vitvi06g00375\_t001 |  | \| \| |  | \| \| |  | \| \| |  |  |  |  |  |  |
| 3 | Vitvi06g04124\_t001 |  | \| \| |  | \| \| |  | \| \| |  |  |  |  |  |  |
| 3 | Vitvi06g00376\_t001 |  | \| \| |  | \| \| |  | \| \| |  |  |  |  |  |  |
| 3 | Vitvi06g01670\_t001 |  | \| \| |  | \| \| |  | \| \| |  |  |  |  |  |  |
| 3 | Vitvi06g00378\_t001 |  | \| \| |  | \| \| |  | \| \| |  |  |  |  |  |  |
| 3 | Vitvi06g00379\_t001 |  | \| \| |  | \| \| |  | Vitvi13g04048\_t001 |  |  |  |  |  |  |
| 3 | Vitvi06g00380\_t001 |  | \| \| |  | Vitvi13g02039\_t001 |  | \| \| |  |  |  |  |  |  |
| 2 | Vitvi06g00381\_t001 |  | \| \| |  |  |  | \| \| |  |  |  |  |  |  |
| 2 | Vitvi06g04125\_t001 |  | \| \| |  |  |  | \| \| |  |  |  |  |  |  |
| 2 | Vitvi06g00382\_t001 |  | Vitvi08g01120\_t001 |  |  |  | \| \| |  |  |  |  |  |  |
| 2 | Vitvi06g00383\_t001 |  | \| \| |  |  |  | \| \| |  |  |  |  |  |  |
| 2 | Vitvi06g01671\_t001 |  | \| \| |  |  |  | \| \| |  |  |  |  |  |  |
| 2 | Vitvi06g01672\_t001 |  | \| \| |  |  |  | \| \| |  |  |  |  |  |  |
| 2 | Vitvi06g01673\_t001 |  | \| \| |  |  |  | \| \| |  |  |  |  |  |  |
| 2 | Vitvi06g01675\_t001 |  | \| \| |  |  |  | \| \| |  |  |  |  |  |  |
| 2 | Vitvi06g01676\_t001 |  | \| \| |  |  |  | \| \| |  |  |  |  |  |  |
| 2 | Vitvi06g00384\_t001 |  | \| \| |  |  |  | \| \| |  |  |  |  |  |  |
| 2 | Vitvi06g04126\_t001 |  | \| \| |  |  |  | \| \| |  |  |  |  |  |  |
| 2 | Vitvi06g04127\_t001 |  | \| \| |  |  |  | \| \| |  |  |  |  |  |  |
| 2 | Vitvi06g00386\_t002 |  | Vitvi08g01111\_t001 |  |  |  | \| \| |  |  |  |  |  |  |
| 2 | Vitvi06g00387\_t001 |  | Vitvi08g04198\_t001 |  |  |  | \| \| |  |  |  |  |  |  |
| 2 | Vitvi06g00388\_t001 |  | \| \| |  |  |  | Vitvi13g00223\_t001 |  |  |  |  |  |  |
| 2 | Vitvi06g04128\_t001 |  | \| \| |  |  |  | \| \| |  |  |  |  |  |  |
| 2 | Vitvi06g04129\_t001 |  | \| \| |  |  |  | \| \| |  |  |  |  |  |  |
| 2 | Vitvi06g01678\_t001 |  | \| \| |  |  |  | \| \| |  |  |  |  |  |  |
| 2 | Vitvi06g00389\_t001 |  | \| \| |  |  |  | \| \| |  |  |  |  |  |  |
| 2 | Vitvi06g04130\_t001 |  | \| \| |  |  |  | \| \| |  |  |  |  |  |  |
| 2 | Vitvi06g04131\_t001 |  | \| \| |  |  |  | \| \| |  |  |  |  |  |  |
| 2 | Vitvi06g00391\_t001 |  | \| \| |  |  |  | \| \| |  |  |  |  |  |  |
| 2 | Vitvi06g01680\_t001 |  | \| \| |  |  |  | Vitvi13g04050\_t001 |  |  |  |  |  |  |
| 2 | Vitvi06g04132\_t001 |  | \| \| |  |  |  | \| \| |  |  |  |  |  |  |
| 2 | Vitvi06g04133\_t001 |  | \| \| |  |  |  | Vitvi13g01927\_t001 |  |  |  |  |  |  |
| 2 | Vitvi06g00392\_t001 |  | \| \| |  |  |  | \| \| |  |  |  |  |  |  |
| 2 | Vitvi06g00393\_t001 |  | Vitvi08g01103\_t001 |  |  |  | Vitvi13g00229\_t001 |  |  |  |  |  |  |
| 2 | Vitvi06g04134\_t001 |  | \| \| |  |  |  | \| \| |  |  |  |  |  |  |
| 2 | Vitvi06g00394\_t001 |  | \| \| |  |  |  | \| \| |  |  |  |  |  |  |
| 2 | Vitvi06g04135\_t001 |  | \| \| |  |  |  | \| \| |  |  |  |  |  |  |
| 2 | Vitvi06g04136\_t001 |  | \| \| |  |  |  | \| \| |  |  |  |  |  |  |
| 2 | Vitvi06g00396\_t001 |  | \| \| |  |  |  | Vitvi13g00230\_t001 |  |  |  |  |  |  |
| 2 | Vitvi06g00397\_t001 |  | Vitvi08g01101\_t001 |  |  |  | Vitvi13g00233\_t001 |  |  |  |  |  |  |
| 2 | Vitvi06g00398\_t001 |  | \| \| |  |  |  | Vitvi13g00234\_t001 |  |  |  |  |  |  |
| 2 | Vitvi06g00399\_t001 |  | Vitvi08g01097\_t001 |  |  |  | \| \| |  |  |  |  |  |  |
| 2 | Vitvi06g04137\_t001 |  | \| \| |  |  |  | \| \| |  |  |  |  |  |  |
| 2 | Vitvi06g00400\_t001 |  | \| \| |  |  |  | Vitvi13g00237\_t001 |  |  |  |  |  |  |
| 1 | Vitvi06g00401\_t001 |  | \| \| |  |  |  |  |  |  |  |  |  |  |
| 1 | Vitvi06g00402\_t001 |  | \| \| |  |  |  |  |  |  |  |  |  |  |
| 1 | Vitvi06g00403\_t001 |  | \| \| |  |  |  |  |  |  |  |  |  |  |
| 1 | Vitvi06g00405\_t001 |  | \| \| |  |  |  |  |  |  |  |  |  |  |
| 1 | Vitvi06g00406\_t001 |  | \| \| |  |  |  |  |  |  |  |  |  |  |
| 1 | Vitvi06g01681\_t001 |  | \| \| |  |  |  |  |  |  |  |  |  |  |
| 1 | Vitvi06g04138\_t001 |  | \| \| |  |  |  |  |  |  |  |  |  |  |
| 1 | Vitvi06g00407\_t001 |  | \| \| |  |  |  |  |  |  |  |  |  |  |
| 2 | Vitvi06g00408\_t001 |  | Vitvi08g01090\_t001 |  | Vitvi13g00249\_t001 |  |  |  |  |  |  |  |  |
| 2 | Vitvi06g04139\_t001 |  | \| \| |  | \| \| |  |  |  |  |  |  |  |  |
| 2 | Vitvi06g00409\_t001 |  | \| \| |  | Vitvi13g00252\_t001 |  |  |  |  |  |  |  |  |
| 2 | Vitvi06g04140\_t001 |  | \| \| |  | \| \| |  |  |  |  |  |  |  |  |
| 2 | Vitvi06g00410\_t001 |  | \| \| |  | \| \| |  |  |  |  |  |  |  |  |
| 2 | Vitvi06g00411\_t001 |  | Vitvi08g02168\_t002 |  | \| \| |  |  |  |  |  |  |  |  |
| 2 | Vitvi06g00412\_t001 |  | \| \| |  | Vitvi13g00255\_t001 |  |  |  |  |  |  |  |  |
| 2 | Vitvi06g00413\_t001 |  | \| \| |  | \| \| |  |  |  |  |  |  |  |  |
| 2 | Vitvi06g04141\_t001 |  | \| \| |  | \| \| |  |  |  |  |  |  |  |  |
| 2 | Vitvi06g00414\_t002 |  | \| \| |  | \| \| |  |  |  |  |  |  |  |  |
| 2 | Vitvi06g04142\_t001 |  | \| \| |  | \| \| |  |  |  |  |  |  |  |  |
| 2 | Vitvi06g00415\_t001 |  | \| \| |  | Vitvi13g00260\_t002 |  |  |  |  |  |  |  |  |
| 2 | Vitvi06g04143\_t001 |  | \| \| |  | \| \| |  |  |  |  |  |  |  |  |
| 2 | Vitvi06g00417\_t001 |  | \| \| |  | \| \| |  |  |  |  |  |  |  |  |
| 2 | Vitvi06g01682\_t001 |  | \| \| |  | Vitvi13g00262\_t001 |  |  |  |  |  |  |  |  |
| 2 | Vitvi06g00419\_t001 |  | Vitvi08g04188\_t001 |  | Vitvi13g00263\_t001 |  |  |  |  |  |  |  |  |
| 2 | Vitvi06g04144\_t001 |  | \| \| |  | \| \| |  |  |  |  |  |  |  |  |
| 2 | Vitvi06g00420\_t001 |  | Vitvi08g01082\_t004 |  | Vitvi13g00266\_t001 |  |  |  |  |  |  |  |  |
| 2 | Vitvi06g01683\_t001 |  | \| \| |  | \| \| |  |  |  |  |  |  |  |  |
| 2 | Vitvi06g00421\_t001 |  | Vitvi08g01079\_t001 |  | \| \| |  |  |  |  |  |  |  |  |
| 1 | Vitvi06g00422\_t001 |  |  |  | \| \| |  |  |  |  |  |  |  |  |
| 1 | Vitvi06g01684\_t001 |  |  |  | \| \| |  |  |  |  |  |  |  |  |
| 1 | Vitvi06g01685\_t001 |  |  |  | \| \| |  |  |  |  |  |  |  |  |
| 1 | Vitvi06g01686\_t001 |  |  |  | \| \| |  |  |  |  |  |  |  |  |
| 1 | Vitvi06g04145\_t001 |  |  |  | \| \| |  |  |  |  |  |  |  |  |
| 1 | Vitvi06g01687\_t001 |  |  |  | \| \| |  |  |  |  |  |  |  |  |
| 1 | Vitvi06g04146\_t001 |  |  |  | \| \| |  |  |  |  |  |  |  |  |
| 2 | Vitvi06g01688\_t001 |  | Vitvi08g01163\_t002 |  | \| \| |  |  |  |  |  |  |  |  |
| 2 | Vitvi06g00423\_t001 |  | \| \| |  | \| \| |  |  |  |  |  |  |  |  |
| 2 | Vitvi06g00424\_t001 |  | Vitvi08g01165\_t001 |  | Vitvi13g00269\_t003 |  |  |  |  |  |  |  |  |
| 2 | Vitvi06g00425\_t001 |  | \| \| |  | \| \| |  |  |  |  |  |  |  |  |
| 2 | Vitvi06g00426\_t001 |  | \| \| |  | \| \| |  |  |  |  |  |  |  |  |
| 2 | Vitvi06g04147\_t001 |  | \| \| |  | \| \| |  |  |  |  |  |  |  |  |
| 2 | Vitvi06g01689\_t001 |  | \| \| |  | \| \| |  |  |  |  |  |  |  |  |
| 2 | Vitvi06g01690\_t001 |  | \| \| |  | \| \| |  |  |  |  |  |  |  |  |
| 2 | Vitvi06g01691\_t001 |  | \| \| |  | \| \| |  |  |  |  |  |  |  |  |
| 2 | Vitvi06g04148\_t001 |  | \| \| |  | \| \| |  |  |  |  |  |  |  |  |
| 2 | Vitvi06g00429\_t002 |  | \| \| |  | Vitvi13g00272\_t001 |  |  |  |  |  |  |  |  |
| 2 | Vitvi06g04149\_t001 |  | \| \| |  | \| \| |  |  |  |  |  |  |  |  |
| 2 | Vitvi06g04150\_t001 |  | \| \| |  | \| \| |  |  |  |  |  |  |  |  |
| 2 | Vitvi06g00430\_t001 |  | \| \| |  | \| \| |  |  |  |  |  |  |  |  |
| 2 | Vitvi06g00432\_t001 |  | \| \| |  | Vitvi13g00275\_t001 |  |  |  |  |  |  |  |  |
| 2 | Vitvi06g04151\_t001 |  | Vitvi08g04213\_t001 |  | Vitvi13g04084\_t001 |  |  |  |  |  |  |  |  |
| 2 | Vitvi06g00434\_t001 |  | Vitvi08g01176\_t001 |  | \| \| |  |  |  |  |  |  |  |  |
| 2 | Vitvi06g00435\_t001 |  | \| \| |  | Vitvi13g01966\_t003 |  |  |  |  |  |  |  |  |
| 2 | Vitvi06g00436\_t001 |  | Vitvi08g01179\_t001 |  | \| \| |  |  |  |  |  |  |  |  |
| 2 | Vitvi06g00437\_t001 |  | \| \| |  | \| \| |  |  |  |  |  |  |  |  |
| 2 | Vitvi06g04152\_t001 |  | \| \| |  | \| \| |  |  |  |  |  |  |  |  |
| 2 | Vitvi06g00438\_t001 |  | \| \| |  | \| \| |  |  |  |  |  |  |  |  |
| 2 | Vitvi06g00439\_t003 |  | Vitvi08g01180\_t001 |  | Vitvi13g00280\_t001 |  |  |  |  |  |  |  |  |
| 2 | Vitvi06g00440\_t001 |  | \| \| |  | \| \| |  |  |  |  |  |  |  |  |
| 2 | Vitvi06g00441\_t001 |  | \| \| |  | \| \| |  |  |  |  |  |  |  |  |
| 2 | Vitvi06g00442\_t001 |  | \| \| |  | \| \| |  |  |  |  |  |  |  |  |
| 2 | Vitvi06g00443\_t001 |  | Vitvi08g02189\_t001 |  | Vitvi13g00281\_t001 |  |  |  |  |  |  |  |  |
| 2 | Vitvi06g00444\_t001 |  | Vitvi08g01182\_t001 |  | Vitvi13g00282\_t002 |  |  |  |  |  |  |  |  |
| 2 | Vitvi06g04153\_t001 |  | \| \| |  | \| \| |  |  |  |  |  |  |  |  |
| 2 | Vitvi06g00445\_t001 |  | \| \| |  | \| \| |  |  |  |  |  |  |  |  |
| 2 | Vitvi06g04154\_t001 |  | \| \| |  | \| \| |  |  |  |  |  |  |  |  |
| 2 | Vitvi06g00447\_t001 |  | \| \| |  | Vitvi13g00285\_t001 |  |  |  |  |  |  |  |  |
| 2 | Vitvi06g04155\_t001 |  | \| \| |  | \| \| |  |  |  |  |  |  |  |  |
| 2 | Vitvi06g01693\_t001 |  | \| \| |  | \| \| |  |  |  |  |  |  |  |  |
| 2 | Vitvi06g00449\_t001 |  | Vitvi08g01186\_t001 |  | \| \| |  |  |  |  |  |  |  |  |
| 2 | Vitvi06g00450\_t001 |  | \| \| |  | \| \| |  |  |  |  |  |  |  |  |
| 2 | Vitvi06g01694\_t001 |  | \| \| |  | \| \| |  |  |  |  |  |  |  |  |
| 2 | Vitvi06g00451\_t001 |  | Vitvi08g01188\_t001 |  | \| \| |  |  |  |  |  |  |  |  |
| 2 | Vitvi06g00452\_t001 |  | Vitvi08g02191\_t001 |  | \| \| |  |  |  |  |  |  |  |  |
| 2 | Vitvi06g01695\_t001 |  | \| \| |  | \| \| |  |  |  |  |  |  |  |  |
| 2 | Vitvi06g00453\_t003 |  | \| \| |  | Vitvi13g01973\_t002 |  |  |  |  |  |  |  |  |
| 1 | Vitvi06g00454\_t001 |  | \| \| |  |  |  |  |  |  |  |  |  |  |
| 1 | Vitvi06g00455\_t001.1.6037826f |  | \| \| |  |  |  |  |  |  |  |  |  |  |
| 1 | Vitvi06g00456\_t001 |  | Vitvi08g01194\_t001 |  |  |  |  |  |  |  |  |  |  |
| 1 | Vitvi06g04156\_t001 |  | \| \| |  |  |  |  |  |  |  |  |  |  |
| 1 | Vitvi06g00459\_t001 |  | \| \| |  |  |  |  |  |  |  |  |  |  |
| 1 | Vitvi06g00460\_t001 |  | Vitvi08g01195\_t001 |  |  |  |  |  |  |  |  |  |  |
| 1 | Vitvi06g00462\_t001 |  | \| \| |  |  |  |  |  |  |  |  |  |  |
| 1 | Vitvi06g00463\_t001 |  | Vitvi08g01196\_t001 |  |  |  |  |  |  |  |  |  |  |
| 1 | Vitvi06g01696\_t003 |  | \| \| |  |  |  |  |  |  |  |  |  |  |
| 1 | Vitvi06g04157\_t001 |  | \| \| |  |  |  |  |  |  |  |  |  |  |
| 1 | Vitvi06g04158\_t001 |  | \| \| |  |  |  |  |  |  |  |  |  |  |
| 1 | Vitvi06g00464\_t001 |  | \| \| |  |  |  |  |  |  |  |  |  |  |
| 1 | Vitvi06g00465\_t001 |  | \| \| |  |  |  |  |  |  |  |  |  |  |
| 1 | Vitvi06g00466\_t001 |  | Vitvi08g02195\_t001 |  |  |  |  |  |  |  |  |  |  |
| 1 | Vitvi06g04159\_t001 |  | \| \| |  |  |  |  |  |  |  |  |  |  |
| 1 | Vitvi06g01700\_t001 |  | \| \| |  |  |  |  |  |  |  |  |  |  |
| 1 | Vitvi06g01701\_t001 |  | \| \| |  |  |  |  |  |  |  |  |  |  |
| 1 | Vitvi06g01702\_t001 |  | \| \| |  |  |  |  |  |  |  |  |  |  |
| 1 | Vitvi06g04160\_t001 |  | \| \| |  |  |  |  |  |  |  |  |  |  |
| 1 | Vitvi06g00467\_t001 |  | \| \| |  |  |  |  |  |  |  |  |  |  |
| 1 | Vitvi06g00468\_t001 |  | \| \| |  |  |  |  |  |  |  |  |  |  |
| 1 | Vitvi06g04161\_t001 |  | \| \| |  |  |  |  |  |  |  |  |  |  |
| 1 | Vitvi06g00470\_t001 |  | \| \| |  |  |  |  |  |  |  |  |  |  |
| 1 | Vitvi06g01703\_t001 |  | \| \| |  |  |  |  |  |  |  |  |  |  |
| 1 | Vitvi06g00471\_t001 |  | \| \| |  |  |  |  |  |  |  |  |  |  |
| 1 | Vitvi06g00472\_t001 |  | \| \| |  |  |  |  |  |  |  |  |  |  |
| 1 | Vitvi06g00473\_t001 |  | Vitvi08g01204\_t004 |  |  |  |  |  |  |  |  |  |  |
| 1 | Vitvi06g00474\_t001 |  | \| \| |  |  |  |  |  |  |  |  |  |  |
| 1 | Vitvi06g00475\_t001 |  | \| \| |  |  |  |  |  |  |  |  |  |  |
| 1 | Vitvi06g00476\_t001 |  | \| \| |  |  |  |  |  |  |  |  |  |  |
| 1 | Vitvi06g04162\_t001 |  | \| \| |  |  |  |  |  |  |  |  |  |  |
| 2 | Vitvi06g00477\_t001 |  | \| \| |  | Vitvi13g01987\_t001 |  |  |  |  |  |  |  |  |
| 2 | Vitvi06g00479\_t001 |  | \| \| |  | \| \| |  |  |  |  |  |  |  |  |
| 2 | Vitvi06g00480\_t001 |  | \| \| |  | \| \| |  |  |  |  |  |  |  |  |
| 2 | Vitvi06g00481\_t001 |  | Vitvi08g01206\_t001 |  | Vitvi13g00309\_t001 |  |  |  |  |  |  |  |  |
| 2 | Vitvi06g01704\_t001 |  | \| \| |  | \| \| |  |  |  |  |  |  |  |  |
| 2 | Vitvi06g04163\_t001 |  | \| \| |  | \| \| |  |  |  |  |  |  |  |  |
| 2 | Vitvi06g00483\_t001 |  | Vitvi08g01209\_t001 |  | \| \| |  |  |  |  |  |  |  |  |
| 2 | Vitvi06g01705\_t001 |  | \| \| |  | \| \| |  |  |  |  |  |  |  |  |
| 2 | Vitvi06g00484\_t001 |  | \| \| |  | \| \| |  |  |  |  |  |  |  |  |
| 2 | Vitvi06g00486\_t001 |  | \| \| |  | \| \| |  |  |  |  |  |  |  |  |
| 2 | Vitvi06g00487\_t001 |  | Vitvi08g01211\_t001 |  | \| \| |  |  |  |  |  |  |  |  |
| 2 | Vitvi06g00488\_t001 |  | Vitvi08g01213\_t002 |  | Vitvi13g00310\_t001 |  |  |  |  |  |  |  |  |
| 2 | Vitvi06g04164\_t001 |  | \| \| |  | \| \| |  |  |  |  |  |  |  |  |
| 2 | Vitvi06g01569\_t001 |  | Vitvi08g01214\_t001 |  | Vitvi13g00311\_t001 |  |  |  |  |  |  |  |  |
| 2 | Vitvi06g00489\_t001 |  | \| \| |  | \| \| |  |  |  |  |  |  |  |  |
| 2 | Vitvi06g00490\_t001 |  | \| \| |  | \| \| |  |  |  |  |  |  |  |  |
| 2 | Vitvi06g00491\_t001 |  | \| \| |  | Vitvi13g00312\_t001 |  |  |  |  |  |  |  |  |
| 2 | Vitvi06g04165\_t001 |  | \| \| |  | \| \| |  |  |  |  |  |  |  |  |
| 2 | Vitvi06g00492\_t001 |  | \| \| |  | \| \| |  |  |  |  |  |  |  |  |
| 2 | Vitvi06g00493\_t001 |  | \| \| |  | \| \| |  |  |  |  |  |  |  |  |
| 2 | Vitvi06g00494\_t001 |  | \| \| |  | \| \| |  |  |  |  |  |  |  |  |
| 2 | Vitvi06g00495\_t001 |  | Vitvi08g01217\_t001 |  | \| \| |  |  |  |  |  |  |  |  |
| 2 | Vitvi06g00496\_t001 |  | Vitvi08g01218\_t001 |  | \| \| |  |  |  |  |  |  |  |  |
| 2 | Vitvi06g00497\_t001 |  | \| \| |  | \| \| |  |  |  |  |  |  |  |  |
| 2 | Vitvi06g00498\_t001 |  | \| \| |  | \| \| |  |  |  |  |  |  |  |  |
| 2 | Vitvi06g01706\_t001 |  | \| \| |  | Vitvi13g01990\_t001 |  |  |  |  |  |  |  |  |
| 2 | Vitvi06g04166\_t001 |  | \| \| |  | Vitvi13g01992\_t001 |  |  |  |  |  |  |  |  |
| 2 | Vitvi06g00500\_t001 |  | \| \| |  | \| \| |  |  |  |  |  |  |  |  |
| 2 | Vitvi06g00501\_t001 |  | \| \| |  | Vitvi13g00324\_t001 |  |  |  |  |  |  |  |  |
| 2 | Vitvi06g00502\_t001 |  | Vitvi08g01232\_t001 |  | Vitvi13g00326\_t001 |  |  |  |  |  |  |  |  |
| 2 | Vitvi06g04167\_t001 |  | \| \| |  | \| \| |  |  |  |  |  |  |  |  |
| 2 | Vitvi06g00503\_t001 |  | \| \| |  | Vitvi13g00328\_t001 |  |  |  |  |  |  |  |  |
| 2 | Vitvi06g01708\_t001 |  | \| \| |  | \| \| |  |  |  |  |  |  |  |  |
| 2 | Vitvi06g00504\_t001 |  | \| \| |  | \| \| |  |  |  |  |  |  |  |  |
| 2 | Vitvi06g04168\_t001 |  | \| \| |  | \| \| |  |  |  |  |  |  |  |  |
| 2 | Vitvi06g00505\_t001 |  | \| \| |  | \| \| |  |  |  |  |  |  |  |  |
| 2 | Vitvi06g00506\_t001 |  | \| \| |  | \| \| |  |  |  |  |  |  |  |  |
| 2 | Vitvi06g00508\_t001 |  | \| \| |  | \| \| |  |  |  |  |  |  |  |  |
| 2 | Vitvi06g00509\_t001 |  | \| \| |  | \| \| |  |  |  |  |  |  |  |  |
| 2 | Vitvi06g00510\_t001 |  | \| \| |  | \| \| |  |  |  |  |  |  |  |  |
| 2 | Vitvi06g00511\_t001 |  | \| \| |  | \| \| |  |  |  |  |  |  |  |  |
| 2 | Vitvi06g00512\_t001 |  | Vitvi08g01241\_t001 |  | Vitvi13g00332\_t001 |  |  |  |  |  |  |  |  |
| 2 | Vitvi06g00513\_t001 |  | Vitvi08g01245\_t001 |  | Vitvi13g01994\_t001 |  |  |  |  |  |  |  |  |
| 2 | Vitvi06g00514\_t001 |  | \| \| |  | \| \| |  |  |  |  |  |  |  |  |
| 2 | Vitvi06g04169\_t001 |  | \| \| |  | \| \| |  |  |  |  |  |  |  |  |
| 2 | Vitvi06g00515\_t001 |  | \| \| |  | \| \| |  |  |  |  |  |  |  |  |
| 2 | Vitvi06g00516\_t001 |  | \| \| |  | \| \| |  |  |  |  |  |  |  |  |
| 2 | Vitvi06g00517\_t001 |  | \| \| |  | \| \| |  |  |  |  |  |  |  |  |
| 2 | Vitvi06g00518\_t001 |  | \| \| |  | \| \| |  |  |  |  |  |  |  |  |
| 2 | Vitvi06g00520\_t001 |  | Vitvi08g01248\_t001 |  | Vitvi13g01996\_t001 |  |  |  |  |  |  |  |  |
| 2 | Vitvi06g01710\_t001 |  | Vitvi08g02203\_t001 |  | Vitvi13g00340\_t001 |  |  |  |  |  |  |  |  |
| 2 | Vitvi06g00521\_t001 |  | Vitvi08g01249\_t001 |  | \| \| |  |  |  |  |  |  |  |  |
| 2 | Vitvi06g00522\_t001 |  | \| \| |  | \| \| |  |  |  |  |  |  |  |  |
| 2 | Vitvi06g04170\_t001 |  | \| \| |  | \| \| |  |  |  |  |  |  |  |  |
| 2 | Vitvi06g01718\_t001 |  | Vitvi08g01250\_t001 |  | Vitvi13g01997\_t001 |  |  |  |  |  |  |  |  |
| 2 | Vitvi06g04171\_t001 |  | \| \| |  | Vitvi13g01998\_t001 |  |  |  |  |  |  |  |  |
| 2 | Vitvi06g04172\_t001 |  | Vitvi08g04238\_t001 |  | \| \| |  |  |  |  |  |  |  |  |
| 2 | Vitvi06g04173\_t001 |  | \| \| |  | \| \| |  |  |  |  |  |  |  |  |
| 2 | Vitvi06g00529\_t001 |  | \| \| |  | \| \| |  |  |  |  |  |  |  |  |
| 2 | Vitvi06g04174\_t001 |  | \| \| |  | \| \| |  |  |  |  |  |  |  |  |
| 2 | Vitvi06g04175\_t001 |  | \| \| |  | \| \| |  |  |  |  |  |  |  |  |
| 2 | Vitvi06g04176\_t001 |  | \| \| |  | \| \| |  |  |  |  |  |  |  |  |
| 2 | Vitvi06g00533\_t001 |  | \| \| |  | Vitvi13g00344\_t001 |  |  |  |  |  |  |  |  |
| 2 | Vitvi06g00534\_t001 |  | \| \| |  | Vitvi13g00345\_t001 |  |  |  |  |  |  |  |  |
| 2 | Vitvi06g04177\_t001 |  | Vitvi08g02206\_t001 |  | \| \| |  |  |  |  |  |  |  |  |
| 2 | Vitvi06g00535\_t001 |  | Vitvi08g01256\_t001 |  | Vitvi13g02003\_t001 |  |  |  |  |  |  |  |  |
| 1 | Vitvi06g00536\_t001 |  |  |  | \| \| |  |  |  |  |  |  |  |  |
| 1 | Vitvi06g00538\_t001 |  |  |  | \| \| |  |  |  |  |  |  |  |  |
| 1 | Vitvi06g04178\_t001 |  |  |  | \| \| |  |  |  |  |  |  |  |  |
| 1 | Vitvi06g00539\_t001 |  |  |  | \| \| |  |  |  |  |  |  |  |  |
| 1 | Vitvi06g00540\_t003 |  |  |  | Vitvi13g00362\_t003 |  |  |  |  |  |  |  |  |
| 1 | Vitvi06g00541\_t001 |  |  |  | \| \| |  |  |  |  |  |  |  |  |
| 1 | Vitvi06g00542\_t001 |  |  |  | Vitvi13g00368\_t001 |  |  |  |  |  |  |  |  |
| 1 | Vitvi06g00543\_t001 |  |  |  | \| \| |  |  |  |  |  |  |  |  |
| 1 | Vitvi06g04179\_t001 |  |  |  | \| \| |  |  |  |  |  |  |  |  |
| 1 | Vitvi06g04180\_t001 |  |  |  | \| \| |  |  |  |  |  |  |  |  |
| 1 | Vitvi06g00546\_t001 |  |  |  | \| \| |  |  |  |  |  |  |  |  |
| 1 | Vitvi06g00547\_t001 |  |  |  | \| \| |  |  |  |  |  |  |  |  |
| 1 | Vitvi06g04181\_t001 |  |  |  | \| \| |  |  |  |  |  |  |  |  |
| 1 | Vitvi06g04182\_t001 |  |  |  | \| \| |  |  |  |  |  |  |  |  |
| 1 | Vitvi06g00548\_t001 |  |  |  | \| \| |  |  |  |  |  |  |  |  |
| 1 | Vitvi06g04183\_t001 |  |  |  | \| \| |  |  |  |  |  |  |  |  |
| 1 | Vitvi06g00549\_t001 |  |  |  | \| \| |  |  |  |  |  |  |  |  |
| 1 | Vitvi06g00550\_t001 |  |  |  | \| \| |  |  |  |  |  |  |  |  |
| 1 | Vitvi06g00552\_t001 |  |  |  | \| \| |  |  |  |  |  |  |  |  |
| 1 | Vitvi06g00553\_t001 |  |  |  | \| \| |  |  |  |  |  |  |  |  |
| 1 | Vitvi06g01722\_t003 |  |  |  | \| \| |  |  |  |  |  |  |  |  |
| 2 | Vitvi06g01723\_t001 |  | Vitvi08g04253\_t006 |  | Vitvi13g00387\_t001 |  |  |  |  |  |  |  |  |
| 2 | Vitvi06g00555\_t001 |  | \| \| |  | Vitvi13g00389\_t001 |  |  |  |  |  |  |  |  |
| 2 | Vitvi06g01724\_t001 |  | Vitvi08g02226\_t002 |  | \| \| |  |  |  |  |  |  |  |  |
| 2 | Vitvi06g01725\_t001 |  | \| \| |  | \| \| |  |  |  |  |  |  |  |  |
| 2 | Vitvi06g04184\_t001 |  | \| \| |  | \| \| |  |  |  |  |  |  |  |  |
| 2 | Vitvi06g04185\_t001 |  | \| \| |  | \| \| |  |  |  |  |  |  |  |  |
| 2 | Vitvi06g04186\_t001 |  | \| \| |  | \| \| |  |  |  |  |  |  |  |  |
| 2 | Vitvi06g01727\_t001 |  | \| \| |  | \| \| |  |  |  |  |  |  |  |  |
| 2 | Vitvi06g01728\_t001 |  | \| \| |  | \| \| |  |  |  |  |  |  |  |  |
| 2 | Vitvi06g04187\_t001 |  | Vitvi08g02228\_t001 |  | \| \| |  |  |  |  |  |  |  |  |
| 2 | Vitvi06g04188\_t001 |  | \| \| |  | \| \| |  |  |  |  |  |  |  |  |
| 2 | Vitvi06g00556\_t001 |  | Vitvi08g01285\_t001 |  | \| \| |  |  |  |  |  |  |  |  |
| 2 | Vitvi06g00558\_t001 |  | Vitvi08g01287\_t001 |  | Vitvi13g02013\_t001 |  |  |  |  |  |  |  |  |
| 2 | Vitvi06g04189\_t001 |  | \| \| |  | \| \| |  |  |  |  |  |  |  |  |
| 2 | Vitvi06g01729\_t001 |  | Vitvi08g02230\_t001 |  | \| \| |  |  |  |  |  |  |  |  |
| 2 | Vitvi06g01730\_t001 |  | \| \| |  | \| \| |  |  |  |  |  |  |  |  |
| 2 | Vitvi06g00559\_t001 |  | Vitvi08g01290\_t001 |  | \| \| |  |  |  |  |  |  |  |  |
| 2 | Vitvi06g00560\_t001 |  | \| \| |  | \| \| |  |  |  |  |  |  |  |  |
| 2 | Vitvi06g00561\_t001 |  | \| \| |  | Vitvi13g02024\_t001 |  |  |  |  |  |  |  |  |
| 2 | Vitvi06g00562\_t001 |  | \| \| |  | Vitvi13g04126\_t001 |  |  |  |  |  |  |  |  |
| 2 | Vitvi06g04190\_t001 |  | \| \| |  | \| \| |  |  |  |  |  |  |  |  |
| 2 | Vitvi06g00563\_t001 |  | Vitvi08g01293\_t001 |  | \| \| |  |  |  |  |  |  |  |  |
| 2 | Vitvi06g00564\_t001 |  | Vitvi08g01295\_t001 |  | \| \| |  |  |  |  |  |  |  |  |
| 2 | Vitvi06g04191\_t001 |  | \| \| |  | \| \| |  |  |  |  |  |  |  |  |
| 2 | Vitvi06g00565\_t001 |  | Vitvi08g01296\_t001 |  | \| \| |  |  |  |  |  |  |  |  |
| 2 | Vitvi06g04192\_t001 |  | \| \| |  | \| \| |  |  |  |  |  |  |  |  |
| 2 | Vitvi06g01732\_t001 |  | \| \| |  | \| \| |  |  |  |  |  |  |  |  |
| 2 | Vitvi06g00567\_t001 |  | Vitvi08g01297\_t001 |  | Vitvi13g04140\_t003 |  |  |  |  |  |  |  |  |
| 2 | Vitvi06g01734\_t001 |  | Vitvi08g01300\_t001 |  | \| \| |  |  |  |  |  |  |  |  |
| 2 | Vitvi06g00568\_t001 |  | \| \| |  | \| \| |  |  |  |  |  |  |  |  |
| 2 | Vitvi06g00569\_t001 |  | Vitvi08g02232\_t002 |  | \| \| |  |  |  |  |  |  |  |  |
| 2 | Vitvi06g04193\_t001 |  | \| \| |  | \| \| |  |  |  |  |  |  |  |  |
| 2 | Vitvi06g00570\_t001 |  | \| \| |  | \| \| |  |  |  |  |  |  |  |  |
| 2 | Vitvi06g00573\_t001 |  | Vitvi08g01311\_t001 |  | \| \| |  |  |  |  |  |  |  |  |
| 2 | Vitvi06g00574\_t001 |  | \| \| |  | \| \| |  |  |  |  |  |  |  |  |
| 2 | Vitvi06g04194\_t001 |  | \| \| |  | \| \| |  |  |  |  |  |  |  |  |
| 2 | Vitvi06g00575\_t001 |  | Vitvi08g01312\_t001 |  | Vitvi13g00501\_t001 |  |  |  |  |  |  |  |  |
| 2 | Vitvi06g00576\_t001 |  | \| \| |  | \| \| |  |  |  |  |  |  |  |  |
| 2 | Vitvi06g01736\_t001 |  | \| \| |  | \| \| |  |  |  |  |  |  |  |  |
| 2 | Vitvi06g00577\_t001.1.6037826e |  | Vitvi08g01313\_t001 |  | \| \| |  |  |  |  |  |  |  |  |
| 2 | Vitvi06g00578\_t001 |  | \| \| |  | \| \| |  |  |  |  |  |  |  |  |
| 2 | Vitvi06g04195\_t001 |  | \| \| |  | \| \| |  |  |  |  |  |  |  |  |
| 2 | Vitvi06g00579\_t001 |  | \| \| |  | \| \| |  |  |  |  |  |  |  |  |
| 2 | Vitvi06g01737\_t001 |  | \| \| |  | \| \| |  |  |  |  |  |  |  |  |
| 2 | Vitvi06g04196\_t001 |  | \| \| |  | \| \| |  |  |  |  |  |  |  |  |
| 2 | Vitvi06g00580\_t001 |  | \| \| |  | \| \| |  |  |  |  |  |  |  |  |
| 2 | Vitvi06g04197\_t001 |  | \| \| |  | \| \| |  |  |  |  |  |  |  |  |
| 2 | Vitvi06g04198\_t001 |  | \| \| |  | \| \| |  |  |  |  |  |  |  |  |
| 2 | Vitvi06g00583\_t001 |  | \| \| |  | \| \| |  |  |  |  |  |  |  |  |
| 2 | Vitvi06g00584\_t001 |  | \| \| |  | \| \| |  |  |  |  |  |  |  |  |
| 2 | Vitvi06g00585\_t001 |  | Vitvi08g01325\_t001 |  | \| \| |  |  |  |  |  |  |  |  |
| 2 | Vitvi06g00586\_t001 |  | \| \| |  | \| \| |  |  |  |  |  |  |  |  |
| 2 | Vitvi06g00587\_t001 |  | Vitvi08g01327\_t001 |  | \| \| |  |  |  |  |  |  |  |  |
| 2 | Vitvi06g04199\_t001 |  | \| \| |  | \| \| |  |  |  |  |  |  |  |  |
| 2 | Vitvi06g00588\_t001 |  | Vitvi08g01329\_t001 |  | \| \| |  |  |  |  |  |  |  |  |
| 2 | Vitvi06g01738\_t001 |  | \| \| |  | \| \| |  |  |  |  |  |  |  |  |
| 2 | Vitvi06g00589\_t001 |  | \| \| |  | \| \| |  |  |  |  |  |  |  |  |
| 2 | Vitvi06g00590\_t001 |  | Vitvi08g01333\_t001 |  | \| \| |  |  |  |  |  |  |  |  |
| 2 | Vitvi06g04200\_t001 |  | \| \| |  | \| \| |  |  |  |  |  |  |  |  |
| 2 | Vitvi06g00591\_t001 |  | Vitvi08g01335\_t001 |  | Vitvi13g00509\_t001 |  |  |  |  |  |  |  |  |
| 2 | Vitvi06g00592\_t001 |  | Vitvi08g01336\_t001 |  | Vitvi13g00510\_t001 |  |  |  |  |  |  |  |  |
| 2 | Vitvi06g00593\_t001 |  | \| \| |  | \| \| |  |  |  |  |  |  |  |  |
| 2 | Vitvi06g04201\_t001 |  | \| \| |  | \| \| |  |  |  |  |  |  |  |  |
| 2 | Vitvi06g00594\_t001 |  | \| \| |  | \| \| |  |  |  |  |  |  |  |  |
| 2 | Vitvi06g00595\_t001 |  | \| \| |  | \| \| |  |  |  |  |  |  |  |  |
| 2 | Vitvi06g01739\_t001 |  | \| \| |  | \| \| |  |  |  |  |  |  |  |  |
| 2 | Vitvi06g00598\_t001 |  | \| \| |  | \| \| |  |  |  |  |  |  |  |  |
| 2 | Vitvi06g00599\_t001 |  | \| \| |  | Vitvi13g00516\_t001 |  |  |  |  |  |  |  |  |
| 1 | Vitvi06g00600\_t001 |  | \| \| |  |  |  |  |  |  |  |  |  |  |
| 1 | Vitvi06g04202\_t001 |  | Vitvi08g02235\_t001 |  |  |  |  |  |  |  |  |  |  |
| 0 | Vitvi06g04203\_t001 |  |  |  |  |  |  |  |  |  |  |  |  |
| 0 | Vitvi06g01740\_t001 |  |  |  |  |  |  |  |  |  |  |  |  |
| 0 | Vitvi06g04204\_t001 |  |  |  |  |  |  |  |  |  |  |  |  |
| 0 | Vitvi06g00601\_t001 |  |  |  |  |  |  |  |  |  |  |  |  |
| 0 | Vitvi06g00602\_t001 |  |  |  |  |  |  |  |  |  |  |  |  |
| 0 | Vitvi06g00604\_t001 |  |  |  |  |  |  |  |  |  |  |  |  |
| 0 | Vitvi06g00605\_t001 |  |  |  |  |  |  |  |  |  |  |  |  |
| 0 | Vitvi06g00606\_t001 |  |  |  |  |  |  |  |  |  |  |  |  |
| 0 | Vitvi06g00607\_t001 |  |  |  |  |  |  |  |  |  |  |  |  |
| 0 | Vitvi06g00608\_t001 |  |  |  |  |  |  |  |  |  |  |  |  |
| 0 | Vitvi06g00610\_t001 |  |  |  |  |  |  |  |  |  |  |  |  |
| 0 | Vitvi06g00611\_t001 |  |  |  |  |  |  |  |  |  |  |  |  |
| 0 | Vitvi06g00613\_t001 |  |  |  |  |  |  |  |  |  |  |  |  |
| 0 | Vitvi06g04205\_t001 |  |  |  |  |  |  |  |  |  |  |  |  |
| 0 | Vitvi06g00614\_t001 |  |  |  |  |  |  |  |  |  |  |  |  |
| 0 | Vitvi06g04206\_t001 |  |  |  |  |  |  |  |  |  |  |  |  |
| 0 | Vitvi06g00615\_t001 |  |  |  |  |  |  |  |  |  |  |  |  |
| 0 | Vitvi06g00617\_t001 |  |  |  |  |  |  |  |  |  |  |  |  |
| 0 | Vitvi06g00618\_t001 |  |  |  |  |  |  |  |  |  |  |  |  |
| 0 | Vitvi06g00619\_t001 |  |  |  |  |  |  |  |  |  |  |  |  |
| 0 | Vitvi06g04207\_t001 |  |  |  |  |  |  |  |  |  |  |  |  |
| 0 | Vitvi06g00621\_t001 |  |  |  |  |  |  |  |  |  |  |  |  |
| 0 | Vitvi06g04208\_t001 |  |  |  |  |  |  |  |  |  |  |  |  |
| 0 | Vitvi06g01743\_t001 |  |  |  |  |  |  |  |  |  |  |  |  |
| 0 | Vitvi06g04209\_t001 |  |  |  |  |  |  |  |  |  |  |  |  |
| 0 | Vitvi06g04210\_t001 |  |  |  |  |  |  |  |  |  |  |  |  |
| 0 | Vitvi06g04211\_t001 |  |  |  |  |  |  |  |  |  |  |  |  |
| 0 | Vitvi06g04212\_t001 |  |  |  |  |  |  |  |  |  |  |  |  |
| 0 | Vitvi06g00625\_t001 |  |  |  |  |  |  |  |  |  |  |  |  |
| 0 | Vitvi06g00626\_t001 |  |  |  |  |  |  |  |  |  |  |  |  |
| 0 | Vitvi06g04213\_t001 |  |  |  |  |  |  |  |  |  |  |  |  |
| 0 | Vitvi06g00627\_t001 |  |  |  |  |  |  |  |  |  |  |  |  |
| 0 | Vitvi06g01746\_t001 |  |  |  |  |  |  |  |  |  |  |  |  |
| 0 | Vitvi06g00629\_t001 |  |  |  |  |  |  |  |  |  |  |  |  |
| 0 | Vitvi06g00630\_t001 |  |  |  |  |  |  |  |  |  |  |  |  |
| 0 | Vitvi06g00631\_t001 |  |  |  |  |  |  |  |  |  |  |  |  |
| 0 | Vitvi06g00632\_t001 |  |  |  |  |  |  |  |  |  |  |  |  |
| 0 | Vitvi06g00633\_t001 |  |  |  |  |  |  |  |  |  |  |  |  |
| 0 | Vitvi06g01748\_t001 |  |  |  |  |  |  |  |  |  |  |  |  |
| 0 | Vitvi06g00634\_t001 |  |  |  |  |  |  |  |  |  |  |  |  |
| 0 | Vitvi06g00635\_t001 |  |  |  |  |  |  |  |  |  |  |  |  |
| 0 | Vitvi06g04214\_t001 |  |  |  |  |  |  |  |  |  |  |  |  |
| 0 | Vitvi06g00637\_t001 |  |  |  |  |  |  |  |  |  |  |  |  |
| 0 | Vitvi06g00638\_t001 |  |  |  |  |  |  |  |  |  |  |  |  |
| 0 | Vitvi06g00639\_t001 |  |  |  |  |  |  |  |  |  |  |  |  |
| 0 | Vitvi06g00640\_t001 |  |  |  |  |  |  |  |  |  |  |  |  |
| 0 | Vitvi06g00641\_t001 |  |  |  |  |  |  |  |  |  |  |  |  |
| 1 | Vitvi06g00642\_t001 |  | Vitvi13g01892\_t001 |  |  |  |  |  |  |  |  |  |  |
| 1 | Vitvi06g00643\_t001 |  | Vitvi13g00046\_t001 |  |  |  |  |  |  |  |  |  |  |
| 2 | Vitvi06g00644\_t001 |  | \| \| |  | Vitvi08g00641\_t001 |  |  |  |  |  |  |  |  |
| 2 | Vitvi06g00645\_t001 |  | \| \| |  | \| \| |  |  |  |  |  |  |  |  |
| 2 | Vitvi06g00646\_t001 |  | \| \| |  | \| \| |  |  |  |  |  |  |  |  |
| 2 | Vitvi06g00647\_t001 |  | Vitvi13g00047\_t001 |  | \| \| |  |  |  |  |  |  |  |  |
| 2 | Vitvi06g00648\_t001 |  | \| \| |  | Vitvi08g00642\_t001 |  |  |  |  |  |  |  |  |
| 2 | Vitvi06g00649\_t001 |  | \| \| |  | \| \| |  |  |  |  |  |  |  |  |
| 2 | Vitvi06g00650\_t001 |  | Vitvi13g00050\_t001 |  | \| \| |  |  |  |  |  |  |  |  |
| 2 | Vitvi06g00651\_t002 |  | \| \| |  | \| \| |  |  |  |  |  |  |  |  |
| 2 | Vitvi06g00652\_t001 |  | Vitvi13g00053\_t001 |  | \| \| |  |  |  |  |  |  |  |  |
| 2 | Vitvi06g00653\_t002 |  | Vitvi13g00058\_t001 |  | \| \| |  |  |  |  |  |  |  |  |
| 2 | Vitvi06g01749\_t001 |  | \| \| |  | Vitvi08g02056\_t001 |  |  |  |  |  |  |  |  |
| 2 | Vitvi06g00654\_t001 |  | Vitvi13g00060\_t001 |  | Vitvi08g00662\_t001 |  |  |  |  |  |  |  |  |
| 2 | Vitvi06g00656\_t001 |  | \| \| |  | \| \| |  |  |  |  |  |  |  |  |
| 2 | Vitvi06g04215\_t001 |  | \| \| |  | \| \| |  |  |  |  |  |  |  |  |
| 2 | Vitvi06g00657\_t001 |  | \| \| |  | \| \| |  |  |  |  |  |  |  |  |
| 2 | Vitvi06g01751\_t001 |  | \| \| |  | \| \| |  |  |  |  |  |  |  |  |
| 2 | Vitvi06g01752\_t001 |  | \| \| |  | \| \| |  |  |  |  |  |  |  |  |
| 2 | Vitvi06g00658\_t001 |  | \| \| |  | Vitvi08g04091\_t001 |  |  |  |  |  |  |  |  |
| 2 | Vitvi06g00659\_t001 |  | \| \| |  | \| \| |  |  |  |  |  |  |  |  |
| 2 | Vitvi06g00661\_t001 |  | \| \| |  | \| \| |  |  |  |  |  |  |  |  |
| 2 | Vitvi06g00662\_t001 |  | \| \| |  | \| \| |  |  |  |  |  |  |  |  |
| 2 | Vitvi06g00665\_t001 |  | \| \| |  | \| \| |  |  |  |  |  |  |  |  |
| 2 | Vitvi06g00666\_t001 |  | \| \| |  | \| \| |  |  |  |  |  |  |  |  |
| 2 | Vitvi06g00667\_t001 |  | Vitvi13g00071\_t001 |  | \| \| |  |  |  |  |  |  |  |  |
| 2 | Vitvi06g00668\_t001 |  | \| \| |  | \| \| |  |  |  |  |  |  |  |  |
| 2 | Vitvi06g00669\_t001 |  | Vitvi13g00072\_t001 |  | \| \| |  |  |  |  |  |  |  |  |
| 2 | Vitvi06g00670\_t001 |  | \| \| |  | Vitvi08g00685\_t001 |  |  |  |  |  |  |  |  |
| 2 | Vitvi06g00671\_t001 |  | \| \| |  | Vitvi08g00686\_t001 |  |  |  |  |  |  |  |  |
| 2 | Vitvi06g04216\_t001 |  | \| \| |  | \| \| |  |  |  |  |  |  |  |  |
| 2 | Vitvi06g00672\_t001 |  | \| \| |  | \| \| |  |  |  |  |  |  |  |  |
| 2 | Vitvi06g00673\_t001 |  | \| \| |  | Vitvi08g00690\_t001 |  |  |  |  |  |  |  |  |
| 1 | Vitvi06g00674\_t001 |  | \| \| |  |  |  |  |  |  |  |  |  |  |
| 1 | Vitvi06g04217\_t001 |  | \| \| |  |  |  |  |  |  |  |  |  |  |
| 1 | Vitvi06g00676\_t001 |  | \| \| |  |  |  |  |  |  |  |  |  |  |
| 1 | Vitvi06g00677\_t001 |  | \| \| |  |  |  |  |  |  |  |  |  |  |
| 2 | Vitvi06g01753\_t001 |  | Vitvi13g00079\_t001 |  | Vitvi08g00718\_t001 |  |  |  |  |  |  |  |  |
| 2 | Vitvi06g00678\_t001 |  | Vitvi13g01860\_t001 |  | \| \| |  |  |  |  |  |  |  |  |
| 2 | Vitvi06g00679\_t001 |  | \| \| |  | \| \| |  |  |  |  |  |  |  |  |
| 2 | Vitvi06g00680\_t001 |  | \| \| |  | Vitvi08g00721\_t001 |  |  |  |  |  |  |  |  |
| 2 | Vitvi06g00681\_t001 |  | \| \| |  | \| \| |  |  |  |  |  |  |  |  |
| 2 | Vitvi06g01754\_t001 |  | \| \| |  | \| \| |  |  |  |  |  |  |  |  |
| 2 | Vitvi06g00683\_t001 |  | \| \| |  | \| \| |  |  |  |  |  |  |  |  |
| 2 | Vitvi06g04218\_t001 |  | \| \| |  | \| \| |  |  |  |  |  |  |  |  |
| 2 | Vitvi06g01755\_t001 |  | \| \| |  | \| \| |  |  |  |  |  |  |  |  |
| 2 | Vitvi06g01756\_t001 |  | \| \| |  | \| \| |  |  |  |  |  |  |  |  |
| 2 | Vitvi06g01757\_t001 |  | \| \| |  | \| \| |  |  |  |  |  |  |  |  |
| 2 | Vitvi06g00686\_t001 |  | Vitvi13g00088\_t001 |  | Vitvi08g00736\_t001 |  |  |  |  |  |  |  |  |
| 2 | Vitvi06g00687\_t001 |  | \| \| |  | \| \| |  |  |  |  |  |  |  |  |
| 2 | Vitvi06g00688\_t001 |  | \| \| |  | Vitvi08g00738\_t001 |  |  |  |  |  |  |  |  |
| 2 | Vitvi06g00689\_t001 |  | Vitvi13g00092\_t001 |  | \| \| |  |  |  |  |  |  |  |  |
| 2 | Vitvi06g04219\_t001 |  | \| \| |  | \| \| |  |  |  |  |  |  |  |  |
| 2 | Vitvi06g00690\_t001 |  | Vitvi13g00097\_t001 |  | \| \| |  |  |  |  |  |  |  |  |
| 2 | Vitvi06g00691\_t001 |  | \| \| |  | \| \| |  |  |  |  |  |  |  |  |
| 2 | Vitvi06g00692\_t003 |  | \| \| |  | Vitvi08g00752\_t001 |  |  |  |  |  |  |  |  |
| 2 | Vitvi06g00693\_t001 |  | \| \| |  | \| \| |  |  |  |  |  |  |  |  |
| 2 | Vitvi06g00694\_t001 |  | \| \| |  | \| \| |  |  |  |  |  |  |  |  |
| 2 | Vitvi06g04220\_t001 |  | \| \| |  | \| \| |  |  |  |  |  |  |  |  |
| 2 | Vitvi06g04221\_t001 |  | \| \| |  | \| \| |  |  |  |  |  |  |  |  |
| 2 | Vitvi06g00695\_t001 |  | \| \| |  | \| \| |  |  |  |  |  |  |  |  |
| 2 | Vitvi06g00696\_t001 |  | Vitvi13g00101\_t001 |  | \| \| |  |  |  |  |  |  |  |  |
| 2 | Vitvi06g04222\_t001 |  | \| \| |  | \| \| |  |  |  |  |  |  |  |  |
| 2 | Vitvi06g04223\_t001 |  | \| \| |  | \| \| |  |  |  |  |  |  |  |  |
| 2 | Vitvi06g01759\_t001 |  | \| \| |  | Vitvi08g00754\_t001 |  |  |  |  |  |  |  |  |
| 2 | Vitvi06g00699\_t001 |  | \| \| |  | \| \| |  |  |  |  |  |  |  |  |
| 2 | Vitvi06g00700\_t001 |  | \| \| |  | \| \| |  |  |  |  |  |  |  |  |
| 2 | Vitvi06g00701\_t001.1.6037826e |  | Vitvi13g00106\_t001 |  | Vitvi08g00757\_t001 |  |  |  |  |  |  |  |  |
| 2 | Vitvi06g01761\_t001 |  | Vitvi13g00107\_t001 |  | Vitvi08g02076\_t001 |  |  |  |  |  |  |  |  |
| 2 | Vitvi06g00702\_t001 |  | \| \| |  | \| \| |  |  |  |  |  |  |  |  |
| 2 | Vitvi06g00704\_t001 |  | \| \| |  | \| \| |  |  |  |  |  |  |  |  |
| 2 | Vitvi06g04224\_t001 |  | \| \| |  | \| \| |  |  |  |  |  |  |  |  |
| 2 | Vitvi06g00705\_t001 |  | \| \| |  | \| \| |  |  |  |  |  |  |  |  |
| 2 | Vitvi06g00706\_t001 |  | Vitvi13g00109\_t001 |  | \| \| |  |  |  |  |  |  |  |  |
| 2 | Vitvi06g00707\_t001 |  | \| \| |  | \| \| |  |  |  |  |  |  |  |  |
| 2 | Vitvi06g00708\_t001 |  | \| \| |  | \| \| |  |  |  |  |  |  |  |  |
| 2 | Vitvi06g01762\_t001 |  | \| \| |  | \| \| |  |  |  |  |  |  |  |  |
| 2 | Vitvi06g00709\_t001 |  | \| \| |  | \| \| |  |  |  |  |  |  |  |  |
| 2 | Vitvi06g01763\_t001 |  | \| \| |  | \| \| |  |  |  |  |  |  |  |  |
| 2 | Vitvi06g04225\_t001 |  | \| \| |  | \| \| |  |  |  |  |  |  |  |  |
| 2 | Vitvi06g04226\_t001 |  | \| \| |  | \| \| |  |  |  |  |  |  |  |  |
| 2 | Vitvi06g04227\_t001 |  | \| \| |  | \| \| |  |  |  |  |  |  |  |  |
| 2 | Vitvi06g00714\_t001 |  | \| \| |  | \| \| |  |  |  |  |  |  |  |  |
| 2 | Vitvi06g00715\_t001 |  | \| \| |  | \| \| |  |  |  |  |  |  |  |  |
| 2 | Vitvi06g04228\_t001 |  | \| \| |  | \| \| |  |  |  |  |  |  |  |  |
| 2 | Vitvi06g04229\_t001 |  | \| \| |  | \| \| |  |  |  |  |  |  |  |  |
| 2 | Vitvi06g04230\_t001 |  | \| \| |  | \| \| |  |  |  |  |  |  |  |  |
| 2 | Vitvi06g00718\_t001 |  | \| \| |  | \| \| |  |  |  |  |  |  |  |  |
| 2 | Vitvi06g04231\_t001 |  | \| \| |  | \| \| |  |  |  |  |  |  |  |  |
| 2 | Vitvi06g00720\_t001 |  | \| \| |  | Vitvi08g00766\_t001 |  |  |  |  |  |  |  |  |
| 2 | Vitvi06g00721\_t001 |  | \| \| |  | \| \| |  |  |  |  |  |  |  |  |
| 2 | Vitvi06g00722\_t001 |  | \| \| |  | \| \| |  |  |  |  |  |  |  |  |
| 2 | Vitvi06g00723\_t001 |  | \| \| |  | \| \| |  |  |  |  |  |  |  |  |
| 2 | Vitvi06g00724\_t001 |  | Vitvi13g00114\_t001 |  | Vitvi08g00768\_t001 |  |  |  |  |  |  |  |  |
| 2 | Vitvi06g00725\_t001 |  | \| \| |  | \| \| |  |  |  |  |  |  |  |  |
| 2 | Vitvi06g00726\_t001 |  | \| \| |  | \| \| |  |  |  |  |  |  |  |  |
| 2 | Vitvi06g04232\_t001 |  | \| \| |  | Vitvi08g00780\_t001 |  |  |  |  |  |  |  |  |
| 2 | Vitvi06g00728\_t001 |  | Vitvi13g00117\_t001 |  | \| \| |  |  |  |  |  |  |  |  |
| 2 | Vitvi06g00729\_t001 |  | \| \| |  | \| \| |  |  |  |  |  |  |  |  |
| 2 | Vitvi06g04233\_t001 |  | \| \| |  | \| \| |  |  |  |  |  |  |  |  |
| 2 | Vitvi06g04234\_t001 |  | \| \| |  | \| \| |  |  |  |  |  |  |  |  |
| 2 | Vitvi06g04235\_t001 |  | \| \| |  | \| \| |  |  |  |  |  |  |  |  |
| 2 | Vitvi06g04236\_t001 |  | \| \| |  | \| \| |  |  |  |  |  |  |  |  |
| 2 | Vitvi06g00731\_t001 |  | \| \| |  | \| \| |  |  |  |  |  |  |  |  |
| 2 | Vitvi06g00732\_t002 |  | Vitvi13g00118\_t001 |  | \| \| |  |  |  |  |  |  |  |  |
| 2 | Vitvi06g00733\_t001 |  | \| \| |  | \| \| |  |  |  |  |  |  |  |  |
| 2 | Vitvi06g00734\_t001 |  | \| \| |  | \| \| |  |  |  |  |  |  |  |  |
| 2 | Vitvi06g00735\_t001 |  | \| \| |  | \| \| |  |  |  |  |  |  |  |  |
| 2 | Vitvi06g00736\_t001 |  | \| \| |  | \| \| |  |  |  |  |  |  |  |  |
| 2 | Vitvi06g00737\_t001 |  | \| \| |  | Vitvi08g00790\_t001 |  |  |  |  |  |  |  |  |
| 2 | Vitvi06g01768\_t001 |  | \| \| |  | \| \| |  |  |  |  |  |  |  |  |
| 2 | Vitvi06g00738\_t001 |  | \| \| |  | \| \| |  |  |  |  |  |  |  |  |
| 2 | Vitvi06g00739\_t001 |  | \| \| |  | \| \| |  |  |  |  |  |  |  |  |
| 2 | Vitvi06g00740\_t001 |  | \| \| |  | Vitvi08g00791\_t001 |  |  |  |  |  |  |  |  |
| 2 | Vitvi06g00741\_t001 |  | \| \| |  | Vitvi08g00793\_t001 |  |  |  |  |  |  |  |  |
| 2 | Vitvi06g00742\_t001 |  | \| \| |  | Vitvi08g00794\_t001 |  |  |  |  |  |  |  |  |
| 2 | Vitvi06g00743\_t001 |  | \| \| |  | \| \| |  |  |  |  |  |  |  |  |
| 2 | Vitvi06g00744\_t001 |  | \| \| |  | Vitvi08g00801\_t001 |  |  |  |  |  |  |  |  |
| 2 | Vitvi06g00745\_t001 |  | Vitvi13g00131\_t001 |  | Vitvi08g00802\_t001 |  |  |  |  |  |  |  |  |
| 2 | Vitvi06g01769\_t001 |  | \| \| |  | \| \| |  |  |  |  |  |  |  |  |
| 2 | Vitvi06g00746\_t001 |  | \| \| |  | \| \| |  |  |  |  |  |  |  |  |
| 2 | Vitvi06g04237\_t001 |  | \| \| |  | \| \| |  |  |  |  |  |  |  |  |
| 2 | Vitvi06g00748\_t001 |  | \| \| |  | \| \| |  |  |  |  |  |  |  |  |
| 2 | Vitvi06g04238\_t001 |  | \| \| |  | \| \| |  |  |  |  |  |  |  |  |
| 2 | Vitvi06g04239\_t001 |  | \| \| |  | \| \| |  |  |  |  |  |  |  |  |
| 2 | Vitvi06g04240\_t001 |  | \| \| |  | \| \| |  |  |  |  |  |  |  |  |
| 2 | Vitvi06g04241\_t001 |  | \| \| |  | \| \| |  |  |  |  |  |  |  |  |
| 2 | Vitvi06g04242\_t001 |  | \| \| |  | \| \| |  |  |  |  |  |  |  |  |
| 2 | Vitvi06g00753\_t001 |  | \| \| |  | \| \| |  |  |  |  |  |  |  |  |
| 2 | Vitvi06g01771\_t001 |  | \| \| |  | \| \| |  |  |  |  |  |  |  |  |
| 2 | Vitvi06g04243\_t001 |  | \| \| |  | \| \| |  |  |  |  |  |  |  |  |
| 2 | Vitvi06g00754\_t001 |  | \| \| |  | \| \| |  |  |  |  |  |  |  |  |
| 2 | Vitvi06g01772\_t001 |  | \| \| |  | Vitvi08g02086\_t001 |  |  |  |  |  |  |  |  |
| 2 | Vitvi06g00757\_t001 |  | \| \| |  | \| \| |  |  |  |  |  |  |  |  |
| 2 | Vitvi06g00758\_t001 |  | \| \| |  | \| \| |  |  |  |  |  |  |  |  |
| 2 | Vitvi06g00759\_t001 |  | \| \| |  | Vitvi08g00816\_t001 |  |  |  |  |  |  |  |  |
| 2 | Vitvi06g01773\_t002 |  | \| \| |  | \| \| |  |  |  |  |  |  |  |  |
| 2 | Vitvi06g00761\_t001 |  | Vitvi13g00138\_t001 |  | \| \| |  |  |  |  |  |  |  |  |
| 2 | Vitvi06g00762\_t001 |  | \| \| |  | \| \| |  |  |  |  |  |  |  |  |
| 2 | Vitvi06g00763\_t001 |  | Vitvi13g00139\_t002 |  | Vitvi08g00820\_t001 |  |  |  |  |  |  |  |  |
| 2 | Vitvi06g00764\_t001 |  | \| \| |  | \| \| |  |  |  |  |  |  |  |  |
| 2 | Vitvi06g04244\_t001 |  | \| \| |  | \| \| |  |  |  |  |  |  |  |  |
| 2 | Vitvi06g00767\_t001 |  | \| \| |  | \| \| |  |  |  |  |  |  |  |  |
| 2 | Vitvi06g00768\_t001 |  | \| \| |  | Vitvi08g00821\_t001 |  |  |  |  |  |  |  |  |
| 2 | Vitvi06g00769\_t001 |  | \| \| |  | Vitvi08g00823\_t001 |  |  |  |  |  |  |  |  |
| 2 | Vitvi06g00770\_t001 |  | \| \| |  | \| \| |  |  |  |  |  |  |  |  |
| 2 | Vitvi06g00771\_t001 |  | Vitvi13g00143\_t001 |  | \| \| |  |  |  |  |  |  |  |  |
| 2 | Vitvi06g04245\_t001 |  | \| \| |  | \| \| |  |  |  |  |  |  |  |  |
| 2 | Vitvi06g00772\_t001 |  | Vitvi13g00144\_t001 |  | \| \| |  |  |  |  |  |  |  |  |
| 2 | Vitvi06g00773\_t002 |  | \| \| |  | \| \| |  |  |  |  |  |  |  |  |
| 2 | Vitvi06g04246\_t001 |  | \| \| |  | \| \| |  |  |  |  |  |  |  |  |
| 2 | Vitvi06g04247\_t001 |  | \| \| |  | \| \| |  |  |  |  |  |  |  |  |
| 2 | Vitvi06g01776\_t001 |  | Vitvi13g01903\_t001 |  | Vitvi08g02089\_t001 |  |  |  |  |  |  |  |  |
| 2 | Vitvi06g00774\_t001 |  | \| \| |  | \| \| |  |  |  |  |  |  |  |  |
| 2 | Vitvi06g00775\_t003 |  | Vitvi13g00149\_t001 |  | Vitvi08g00828\_t001 |  |  |  |  |  |  |  |  |
| 2 | Vitvi06g00776\_t001 |  | \| \| |  | \| \| |  |  |  |  |  |  |  |  |
| 2 | Vitvi06g01777\_t001 |  | \| \| |  | \| \| |  |  |  |  |  |  |  |  |
| 2 | Vitvi06g00777\_t001 |  | \| \| |  | \| \| |  |  |  |  |  |  |  |  |
| 2 | Vitvi06g00779\_t001 |  | \| \| |  | Vitvi08g00835\_t001 |  |  |  |  |  |  |  |  |
| 2 | Vitvi06g00780\_t001 |  | \| \| |  | \| \| |  |  |  |  |  |  |  |  |
| 2 | Vitvi06g00781\_t001 |  | \| \| |  | \| \| |  |  |  |  |  |  |  |  |
| 2 | Vitvi06g00782\_t001 |  | \| \| |  | \| \| |  |  |  |  |  |  |  |  |
| 2 | Vitvi06g04248\_t001 |  | \| \| |  | \| \| |  |  |  |  |  |  |  |  |
| 2 | Vitvi06g00783\_t001 |  | \| \| |  | Vitvi08g00840\_t001 |  |  |  |  |  |  |  |  |
| 2 | Vitvi06g00784\_t001 |  | \| \| |  | Vitvi08g00843\_t001 |  |  |  |  |  |  |  |  |
| 2 | Vitvi06g00785\_t001 |  | Vitvi13g00158\_t001 |  | Vitvi08g00844\_t001 |  |  |  |  |  |  |  |  |
| 2 | Vitvi06g00786\_t001 |  | Vitvi13g00160\_t001 |  | \| \| |  |  |  |  |  |  |  |  |
| 2 | Vitvi06g00787\_t001 |  | Vitvi13g00177\_t001 |  | \| \| |  |  |  |  |  |  |  |  |
| 2 | Vitvi06g00789\_t002 |  | \| \| |  | \| \| |  |  |  |  |  |  |  |  |
| 2 | Vitvi06g00790\_t001 |  | \| \| |  | \| \| |  |  |  |  |  |  |  |  |
| 2 | Vitvi06g00791\_t001 |  | \| \| |  | \| \| |  |  |  |  |  |  |  |  |
| 2 | Vitvi06g01778\_t001 |  | \| \| |  | \| \| |  |  |  |  |  |  |  |  |
| 2 | Vitvi06g04249\_t001 |  | \| \| |  | \| \| |  |  |  |  |  |  |  |  |
| 2 | Vitvi06g04250\_t001 |  | \| \| |  | \| \| |  |  |  |  |  |  |  |  |
| 2 | Vitvi06g00796\_t001 |  | \| \| |  | \| \| |  |  |  |  |  |  |  |  |
| 2 | Vitvi06g00798\_t001 |  | \| \| |  | \| \| |  |  |  |  |  |  |  |  |
| 2 | Vitvi06g00799\_t001 |  | \| \| |  | Vitvi08g00853\_t001 |  |  |  |  |  |  |  |  |
| 2 | Vitvi06g00800\_t001 |  | \| \| |  | \| \| |  |  |  |  |  |  |  |  |
| 2 | Vitvi06g04251\_t001 |  | \| \| |  | \| \| |  |  |  |  |  |  |  |  |
| 2 | Vitvi06g00801\_t001 |  | \| \| |  | Vitvi08g00857\_t001 |  |  |  |  |  |  |  |  |
| 2 | Vitvi06g04252\_t001 |  | \| \| |  | \| \| |  |  |  |  |  |  |  |  |
| 2 | Vitvi06g04253\_t001 |  | \| \| |  | Vitvi08g00859\_t001 |  |  |  |  |  |  |  |  |
| 2 | Vitvi06g04254\_t001 |  | \| \| |  | \| \| |  |  |  |  |  |  |  |  |
| 2 | Vitvi06g01781\_t001 |  | \| \| |  | \| \| |  |  |  |  |  |  |  |  |
| 2 | Vitvi06g00803\_t001 |  | \| \| |  | \| \| |  |  |  |  |  |  |  |  |
| 2 | Vitvi06g00804\_t001 |  | \| \| |  | \| \| |  |  |  |  |  |  |  |  |
| 2 | Vitvi06g00805\_t001 |  | Vitvi13g00195\_t001 |  | \| \| |  |  |  |  |  |  |  |  |
| 1 | Vitvi06g04255\_t001 |  |  |  | \| \| |  |  |  |  |  |  |  |  |
| 1 | Vitvi06g01782\_t001 |  |  |  | \| \| |  |  |  |  |  |  |  |  |
| 1 | Vitvi06g00807\_t001 |  |  |  | \| \| |  |  |  |  |  |  |  |  |
| 1 | Vitvi06g04256\_t001 |  |  |  | \| \| |  |  |  |  |  |  |  |  |
| 1 | Vitvi06g00808\_t001 |  |  |  | \| \| |  |  |  |  |  |  |  |  |
| 1 | Vitvi06g00810\_t001 |  |  |  | \| \| |  |  |  |  |  |  |  |  |
| 1 | Vitvi06g00811\_t001 |  |  |  | Vitvi08g04142\_t002 |  |  |  |  |  |  |  |  |
| 1 | Vitvi06g00812\_t001 |  |  |  | Vitvi08g00883\_t004 |  |  |  |  |  |  |  |  |
| 0 | Vitvi06g00813\_t001 |  |  |  |  |  |  |  |  |  |  |  |  |
| 0 | Vitvi06g04257\_t001 |  |  |  |  |  |  |  |  |  |  |  |  |
| 0 | Vitvi06g00814\_t001 |  |  |  |  |  |  |  |  |  |  |  |  |
| 0 | Vitvi06g00817\_t001 |  |  |  |  |  |  |  |  |  |  |  |  |
| 0 | Vitvi06g00819\_t002 |  |  |  |  |  |  |  |  |  |  |  |  |
| 0 | Vitvi06g04258\_t001 |  |  |  |  |  |  |  |  |  |  |  |  |
| 0 | Vitvi06g00822\_t001 |  |  |  |  |  |  |  |  |  |  |  |  |
| 0 | Vitvi06g00823\_t001 |  |  |  |  |  |  |  |  |  |  |  |  |
| 0 | Vitvi06g00824\_t001 |  |  |  |  |  |  |  |  |  |  |  |  |
| 0 | Vitvi06g00825\_t001 |  |  |  |  |  |  |  |  |  |  |  |  |
| 0 | Vitvi06g00826\_t001 |  |  |  |  |  |  |  |  |  |  |  |  |
| 0 | Vitvi06g00827\_t001 |  |  |  |  |  |  |  |  |  |  |  |  |
| 0 | Vitvi06g00829\_t001 |  |  |  |  |  |  |  |  |  |  |  |  |
| 0 | Vitvi06g00831\_t001 |  |  |  |  |  |  |  |  |  |  |  |  |
| 0 | Vitvi06g00832\_t001 |  |  |  |  |  |  |  |  |  |  |  |  |
| 0 | Vitvi06g00833\_t001 |  |  |  |  |  |  |  |  |  |  |  |  |
| 0 | Vitvi06g00834\_t002 |  |  |  |  |  |  |  |  |  |  |  |  |
| 0 | Vitvi06g00836\_t001 |  |  |  |  |  |  |  |  |  |  |  |  |
| 0 | Vitvi06g00840\_t001 |  |  |  |  |  |  |  |  |  |  |  |  |
| 0 | Vitvi06g00841\_t001 |  |  |  |  |  |  |  |  |  |  |  |  |
| 0 | Vitvi06g00843\_t001 |  |  |  |  |  |  |  |  |  |  |  |  |
| 0 | Vitvi06g00844\_t002 |  |  |  |  |  |  |  |  |  |  |  |  |
| 0 | Vitvi06g00845\_t001 |  |  |  |  |  |  |  |  |  |  |  |  |
| 0 | Vitvi06g04259\_t001 |  |  |  |  |  |  |  |  |  |  |  |  |
| 0 | Vitvi06g04260\_t001 |  |  |  |  |  |  |  |  |  |  |  |  |
| 0 | Vitvi06g00846\_t001 |  |  |  |  |  |  |  |  |  |  |  |  |
| 0 | Vitvi06g00848\_t001 |  |  |  |  |  |  |  |  |  |  |  |  |
| 0 | Vitvi06g04261\_t001 |  |  |  |  |  |  |  |  |  |  |  |  |
| 0 | Vitvi06g04262\_t001 |  |  |  |  |  |  |  |  |  |  |  |  |
| 0 | Vitvi06g04263\_t001 |  |  |  |  |  |  |  |  |  |  |  |  |
| 0 | Vitvi06g04264\_t001 |  |  |  |  |  |  |  |  |  |  |  |  |
| 0 | Vitvi06g00876\_t001 |  |  |  |  |  |  |  |  |  |  |  |  |
| 0 | Vitvi06g04265\_t001 |  |  |  |  |  |  |  |  |  |  |  |  |
| 0 | Vitvi06g04266\_t001 |  |  |  |  |  |  |  |  |  |  |  |  |
| 0 | Vitvi06g04267\_t001 |  |  |  |  |  |  |  |  |  |  |  |  |
| 0 | Vitvi06g00893\_t001 |  |  |  |  |  |  |  |  |  |  |  |  |
| 0 | Vitvi06g04268\_t001 |  |  |  |  |  |  |  |  |  |  |  |  |
| 0 | Vitvi06g00894\_t001 |  |  |  |  |  |  |  |  |  |  |  |  |
| 0 | Vitvi06g00895\_t001 |  |  |  |  |  |  |  |  |  |  |  |  |
| 0 | Vitvi06g00896\_t001 |  |  |  |  |  |  |  |  |  |  |  |  |
| 0 | Vitvi06g00897\_t001 |  |  |  |  |  |  |  |  |  |  |  |  |
| 0 | Vitvi06g00898\_t001 |  |  |  |  |  |  |  |  |  |  |  |  |
| 0 | Vitvi06g04269\_t001 |  |  |  |  |  |  |  |  |  |  |  |  |
| 0 | Vitvi06g00899\_t001 |  |  |  |  |  |  |  |  |  |  |  |  |
| 0 | Vitvi06g04270\_t001 |  |  |  |  |  |  |  |  |  |  |  |  |
| 0 | Vitvi06g04271\_t001 |  |  |  |  |  |  |  |  |  |  |  |  |
| 0 | Vitvi06g04272\_t001 |  |  |  |  |  |  |  |  |  |  |  |  |
| 0 | Vitvi06g04273\_t001 |  |  |  |  |  |  |  |  |  |  |  |  |
| 0 | Vitvi06g00903\_t001 |  |  |  |  |  |  |  |  |  |  |  |  |
| 0 | Vitvi06g00906\_t001 |  |  |  |  |  |  |  |  |  |  |  |  |
| 0 | Vitvi06g00910\_t002 |  |  |  |  |  |  |  |  |  |  |  |  |
| 0 | Vitvi06g04274\_t001 |  |  |  |  |  |  |  |  |  |  |  |  |
| 0 | Vitvi06g04275\_t001 |  |  |  |  |  |  |  |  |  |  |  |  |
| 0 | Vitvi06g04276\_t001 |  |  |  |  |  |  |  |  |  |  |  |  |
| 0 | Vitvi06g00915\_t001 |  |  |  |  |  |  |  |  |  |  |  |  |
| 0 | Vitvi06g00916\_t001 |  |  |  |  |  |  |  |  |  |  |  |  |
| 0 | Vitvi06g00917\_t001 |  |  |  |  |  |  |  |  |  |  |  |  |
| 0 | Vitvi06g00918\_t001 |  |  |  |  |  |  |  |  |  |  |  |  |
| 0 | Vitvi06g00920\_t001 |  |  |  |  |  |  |  |  |  |  |  |  |
| 0 | Vitvi06g00922\_t001 |  |  |  |  |  |  |  |  |  |  |  |  |
| 0 | Vitvi06g00928\_t001 |  |  |  |  |  |  |  |  |  |  |  |  |
| 0 | Vitvi06g04277\_t001 |  |  |  |  |  |  |  |  |  |  |  |  |
| 0 | Vitvi06g04278\_t001 |  |  |  |  |  |  |  |  |  |  |  |  |
| 0 | Vitvi06g04279\_t001 |  |  |  |  |  |  |  |  |  |  |  |  |
| 0 | Vitvi06g00931\_t001 |  |  |  |  |  |  |  |  |  |  |  |  |
| 0 | Vitvi06g00932\_t001 |  |  |  |  |  |  |  |  |  |  |  |  |
| 0 | Vitvi06g04280\_t001 |  |  |  |  |  |  |  |  |  |  |  |  |
| 0 | Vitvi06g04281\_t001 |  |  |  |  |  |  |  |  |  |  |  |  |
| 0 | Vitvi06g00937\_t001 |  |  |  |  |  |  |  |  |  |  |  |  |
| 0 | Vitvi06g04282\_t001 |  |  |  |  |  |  |  |  |  |  |  |  |
| 0 | Vitvi06g04283\_t001 |  |  |  |  |  |  |  |  |  |  |  |  |
| 0 | Vitvi06g00943\_t001 |  |  |  |  |  |  |  |  |  |  |  |  |
| 0 | Vitvi06g00944\_t001 |  |  |  |  |  |  |  |  |  |  |  |  |
| 0 | Vitvi06g00946\_t001 |  |  |  |  |  |  |  |  |  |  |  |  |
| 0 | Vitvi06g00947\_t001 |  |  |  |  |  |  |  |  |  |  |  |  |
| 0 | Vitvi06g01810\_t001 |  |  |  |  |  |  |  |  |  |  |  |  |
| 0 | Vitvi06g04284\_t001 |  |  |  |  |  |  |  |  |  |  |  |  |
| 0 | Vitvi06g01811\_t001 |  |  |  |  |  |  |  |  |  |  |  |  |
| 0 | Vitvi06g00956\_t001 |  |  |  |  |  |  |  |  |  |  |  |  |
| 0 | Vitvi06g00959\_t001 |  |  |  |  |  |  |  |  |  |  |  |  |
| 0 | Vitvi06g04285\_t001 |  |  |  |  |  |  |  |  |  |  |  |  |
| 0 | Vitvi06g00961\_t001 |  |  |  |  |  |  |  |  |  |  |  |  |
| 0 | Vitvi06g00962\_t003 |  |  |  |  |  |  |  |  |  |  |  |  |
| 0 | Vitvi06g00964\_t001 |  |  |  |  |  |  |  |  |  |  |  |  |
| 0 | Vitvi06g00966\_t001 |  |  |  |  |  |  |  |  |  |  |  |  |
| 0 | Vitvi06g00968\_t001 |  |  |  |  |  |  |  |  |  |  |  |  |
| 0 | Vitvi06g04286\_t001 |  |  |  |  |  |  |  |  |  |  |  |  |
| 0 | Vitvi06g00972\_t001 |  |  |  |  |  |  |  |  |  |  |  |  |
| 0 | Vitvi06g00973\_t001 |  |  |  |  |  |  |  |  |  |  |  |  |
| 0 | Vitvi06g00975\_t001 |  |  |  |  |  |  |  |  |  |  |  |  |
| 0 | Vitvi06g04287\_t001 |  |  |  |  |  |  |  |  |  |  |  |  |
| 0 | Vitvi06g00978\_t001 |  |  |  |  |  |  |  |  |  |  |  |  |
| 0 | Vitvi06g00979\_t001 |  |  |  |  |  |  |  |  |  |  |  |  |
| 0 | Vitvi06g00982\_t001 |  |  |  |  |  |  |  |  |  |  |  |  |
| 0 | Vitvi06g04288\_t001 |  |  |  |  |  |  |  |  |  |  |  |  |
| 0 | Vitvi06g01818\_t001 |  |  |  |  |  |  |  |  |  |  |  |  |
| 0 | Vitvi06g00984\_t001 |  |  |  |  |  |  |  |  |  |  |  |  |
| 0 | Vitvi06g00985\_t001 |  |  |  |  |  |  |  |  |  |  |  |  |
| 0 | Vitvi06g00988\_t001 |  |  |  |  |  |  |  |  |  |  |  |  |
| 0 | Vitvi06g01819\_t001 |  |  |  |  |  |  |  |  |  |  |  |  |
| 0 | Vitvi06g00990\_t001 |  |  |  |  |  |  |  |  |  |  |  |  |
| 0 | Vitvi06g00991\_t002 |  |  |  |  |  |  |  |  |  |  |  |  |
| 0 | Vitvi06g00992\_t001 |  |  |  |  |  |  |  |  |  |  |  |  |
| 0 | Vitvi06g00993\_t001 |  |  |  |  |  |  |  |  |  |  |  |  |
| 0 | Vitvi06g00994\_t001 |  |  |  |  |  |  |  |  |  |  |  |  |
| 0 | Vitvi06g00995\_t001 |  |  |  |  |  |  |  |  |  |  |  |  |
| 0 | Vitvi06g00996\_t001 |  |  |  |  |  |  |  |  |  |  |  |  |
| 0 | Vitvi06g00997\_t001 |  |  |  |  |  |  |  |  |  |  |  |  |
| 0 | Vitvi06g01821\_t001 |  |  |  |  |  |  |  |  |  |  |  |  |
| 0 | Vitvi06g00998\_t001 |  |  |  |  |  |  |  |  |  |  |  |  |
| 0 | Vitvi06g00999\_t001 |  |  |  |  |  |  |  |  |  |  |  |  |
| 0 | Vitvi06g01000\_t001 |  |  |  |  |  |  |  |  |  |  |  |  |
| 0 | Vitvi06g04289\_t001 |  |  |  |  |  |  |  |  |  |  |  |  |
| 0 | Vitvi06g01004\_t002 |  |  |  |  |  |  |  |  |  |  |  |  |
| 0 | Vitvi06g01010\_t001 |  |  |  |  |  |  |  |  |  |  |  |  |
| 0 | Vitvi06g01011\_t001 |  |  |  |  |  |  |  |  |  |  |  |  |
| 0 | Vitvi06g04290\_t001 |  |  |  |  |  |  |  |  |  |  |  |  |
| 0 | Vitvi06g04291\_t001 |  |  |  |  |  |  |  |  |  |  |  |  |
| 0 | Vitvi06g04292\_t001 |  |  |  |  |  |  |  |  |  |  |  |  |
| 0 | Vitvi06g01017\_t001 |  |  |  |  |  |  |  |  |  |  |  |  |
| 0 | Vitvi06g04293\_t001 |  |  |  |  |  |  |  |  |  |  |  |  |
| 0 | Vitvi06g01020\_t001 |  |  |  |  |  |  |  |  |  |  |  |  |
| 0 | Vitvi06g01021\_t001 |  |  |  |  |  |  |  |  |  |  |  |  |
| 0 | Vitvi06g01023\_t001 |  |  |  |  |  |  |  |  |  |  |  |  |
| 0 | Vitvi06g01024\_t001 |  |  |  |  |  |  |  |  |  |  |  |  |
| 0 | Vitvi06g01026\_t001 |  |  |  |  |  |  |  |  |  |  |  |  |
| 0 | Vitvi06g01027\_t001 |  |  |  |  |  |  |  |  |  |  |  |  |
| 0 | Vitvi06g01033\_t001 |  |  |  |  |  |  |  |  |  |  |  |  |
| 0 | Vitvi06g01827\_t001 |  |  |  |  |  |  |  |  |  |  |  |  |
| 0 | Vitvi06g01034\_t001 |  |  |  |  |  |  |  |  |  |  |  |  |
| 0 | Vitvi06g01035\_t001 |  |  |  |  |  |  |  |  |  |  |  |  |
| 0 | Vitvi06g01036\_t001 |  |  |  |  |  |  |  |  |  |  |  |  |
| 0 | Vitvi06g01829\_t001 |  |  |  |  |  |  |  |  |  |  |  |  |
| 0 | Vitvi06g01830\_t002 |  |  |  |  |  |  |  |  |  |  |  |  |
| 0 | Vitvi06g04294\_t001 |  |  |  |  |  |  |  |  |  |  |  |  |
| 0 | Vitvi06g04295\_t001 |  |  |  |  |  |  |  |  |  |  |  |  |
| 0 | Vitvi06g04296\_t001 |  |  |  |  |  |  |  |  |  |  |  |  |
| 0 | Vitvi06g04297\_t001 |  |  |  |  |  |  |  |  |  |  |  |  |
| 0 | Vitvi06g04298\_t001 |  |  |  |  |  |  |  |  |  |  |  |  |
| 0 | Vitvi06g01043\_t001 |  |  |  |  |  |  |  |  |  |  |  |  |
| 0 | Vitvi06g01044\_t001 |  |  |  |  |  |  |  |  |  |  |  |  |
| 0 | Vitvi06g01045\_t001 |  |  |  |  |  |  |  |  |  |  |  |  |
| 0 | Vitvi06g01834\_t001 |  |  |  |  |  |  |  |  |  |  |  |  |
| 0 | Vitvi06g01047\_t001 |  |  |  |  |  |  |  |  |  |  |  |  |
| 0 | Vitvi06g01835\_t001 |  |  |  |  |  |  |  |  |  |  |  |  |
| 0 | Vitvi06g04299\_t001 |  |  |  |  |  |  |  |  |  |  |  |  |
| 0 | Vitvi06g01048\_t001 |  |  |  |  |  |  |  |  |  |  |  |  |
| 0 | Vitvi06g01050\_t001 |  |  |  |  |  |  |  |  |  |  |  |  |
| 0 | Vitvi06g04300\_t001 |  |  |  |  |  |  |  |  |  |  |  |  |
| 0 | Vitvi06g01051\_t001 |  |  |  |  |  |  |  |  |  |  |  |  |
| 0 | Vitvi06g01052\_t001 |  |  |  |  |  |  |  |  |  |  |  |  |
| 0 | Vitvi06g01053\_t001 |  |  |  |  |  |  |  |  |  |  |  |  |
| 0 | Vitvi06g01055\_t001 |  |  |  |  |  |  |  |  |  |  |  |  |
| 0 | Vitvi06g04301\_t001 |  |  |  |  |  |  |  |  |  |  |  |  |
| 0 | Vitvi06g01056\_t001 |  |  |  |  |  |  |  |  |  |  |  |  |
| 0 | Vitvi06g01057\_t001 |  |  |  |  |  |  |  |  |  |  |  |  |
| 0 | Vitvi06g01058\_t001 |  |  |  |  |  |  |  |  |  |  |  |  |
| 0 | Vitvi06g01060\_t001 |  |  |  |  |  |  |  |  |  |  |  |  |
| 0 | Vitvi06g01061\_t001 |  |  |  |  |  |  |  |  |  |  |  |  |
| 0 | Vitvi06g04302\_t001 |  |  |  |  |  |  |  |  |  |  |  |  |
| 0 | Vitvi06g04303\_t001 |  |  |  |  |  |  |  |  |  |  |  |  |
| 0 | Vitvi06g04304\_t001 |  |  |  |  |  |  |  |  |  |  |  |  |
| 0 | Vitvi06g01064\_t001 |  |  |  |  |  |  |  |  |  |  |  |  |
| 0 | Vitvi06g04305\_t001 |  |  |  |  |  |  |  |  |  |  |  |  |
| 0 | Vitvi06g01069\_t001 |  |  |  |  |  |  |  |  |  |  |  |  |
| 0 | Vitvi06g01070\_t001 |  |  |  |  |  |  |  |  |  |  |  |  |
| 0 | Vitvi06g01071\_t001 |  |  |  |  |  |  |  |  |  |  |  |  |
| 0 | Vitvi06g01839\_t001 |  |  |  |  |  |  |  |  |  |  |  |  |
| 0 | Vitvi06g01072\_t001 |  |  |  |  |  |  |  |  |  |  |  |  |
| 0 | Vitvi06g01073\_t004 |  |  |  |  |  |  |  |  |  |  |  |  |
| 0 | Vitvi06g01074\_t002 |  |  |  |  |  |  |  |  |  |  |  |  |
| 0 | Vitvi06g01075\_t001 |  |  |  |  |  |  |  |  |  |  |  |  |
| 0 | Vitvi06g01078\_t001 |  |  |  |  |  |  |  |  |  |  |  |  |
| 0 | Vitvi06g04306\_t001 |  |  |  |  |  |  |  |  |  |  |  |  |
| 0 | Vitvi06g04307\_t001 |  |  |  |  |  |  |  |  |  |  |  |  |
| 0 | Vitvi06g01084\_t003 |  |  |  |  |  |  |  |  |  |  |  |  |
| 0 | Vitvi06g04309\_t001 |  |  |  |  |  |  |  |  |  |  |  |  |
| 0 | Vitvi06g01842\_t001 |  |  |  |  |  |  |  |  |  |  |  |  |
| 0 | Vitvi06g01843\_t001 |  |  |  |  |  |  |  |  |  |  |  |  |
| 0 | Vitvi06g01086\_t003 |  |  |  |  |  |  |  |  |  |  |  |  |
| 0 | Vitvi06g01087\_t001 |  |  |  |  |  |  |  |  |  |  |  |  |
| 0 | Vitvi06g01088\_t001 |  |  |  |  |  |  |  |  |  |  |  |  |
| 0 | Vitvi06g04310\_t001 |  |  |  |  |  |  |  |  |  |  |  |  |
| 0 | Vitvi06g01844\_t001 |  |  |  |  |  |  |  |  |  |  |  |  |
| 0 | Vitvi06g01091\_t001 |  |  |  |  |  |  |  |  |  |  |  |  |
| 0 | Vitvi06g01092\_t002 |  |  |  |  |  |  |  |  |  |  |  |  |
| 0 | Vitvi06g01093\_t001 |  |  |  |  |  |  |  |  |  |  |  |  |
| 0 | Vitvi06g01094\_t001 |  |  |  |  |  |  |  |  |  |  |  |  |
| 0 | Vitvi06g01095\_t001 |  |  |  |  |  |  |  |  |  |  |  |  |
| 0 | Vitvi06g01846\_t001 |  |  |  |  |  |  |  |  |  |  |  |  |
| 0 | Vitvi06g01097\_t001 |  |  |  |  |  |  |  |  |  |  |  |  |
| 0 | Vitvi06g04311\_t001 |  |  |  |  |  |  |  |  |  |  |  |  |
| 0 | Vitvi06g01100\_t001 |  |  |  |  |  |  |  |  |  |  |  |  |
| 0 | Vitvi06g01101\_t001 |  |  |  |  |  |  |  |  |  |  |  |  |
| 0 | Vitvi06g01847\_t001 |  |  |  |  |  |  |  |  |  |  |  |  |
| 0 | Vitvi06g01104\_t001 |  |  |  |  |  |  |  |  |  |  |  |  |
| 0 | Vitvi06g04312\_t001 |  |  |  |  |  |  |  |  |  |  |  |  |
| 0 | Vitvi06g01850\_t001 |  |  |  |  |  |  |  |  |  |  |  |  |
| 0 | Vitvi06g01851\_t001 |  |  |  |  |  |  |  |  |  |  |  |  |
| 0 | Vitvi06g01106\_t001 |  |  |  |  |  |  |  |  |  |  |  |  |
| 0 | Vitvi06g01107\_t002 |  |  |  |  |  |  |  |  |  |  |  |  |
| 0 | Vitvi06g01108\_t001 |  |  |  |  |  |  |  |  |  |  |  |  |
| 0 | Vitvi06g01109\_t001 |  |  |  |  |  |  |  |  |  |  |  |  |
| 0 | Vitvi06g01852\_t001 |  |  |  |  |  |  |  |  |  |  |  |  |
| 0 | Vitvi06g04313\_t001 |  |  |  |  |  |  |  |  |  |  |  |  |
| 0 | Vitvi06g01110\_t001 |  |  |  |  |  |  |  |  |  |  |  |  |
| 0 | Vitvi06g04314\_t001 |  |  |  |  |  |  |  |  |  |  |  |  |
| 0 | Vitvi06g01111\_t001 |  |  |  |  |  |  |  |  |  |  |  |  |
| 0 | Vitvi06g04315\_t001 |  |  |  |  |  |  |  |  |  |  |  |  |
| 0 | Vitvi06g01112\_t001 |  |  |  |  |  |  |  |  |  |  |  |  |
| 0 | Vitvi06g01113\_t001 |  |  |  |  |  |  |  |  |  |  |  |  |
| 0 | Vitvi06g01114\_t002 |  |  |  |  |  |  |  |  |  |  |  |  |
| 0 | Vitvi06g01116\_t001 |  |  |  |  |  |  |  |  |  |  |  |  |
| 0 | Vitvi06g01117\_t001 |  |  |  |  |  |  |  |  |  |  |  |  |
| 0 | Vitvi06g04316\_t001 |  |  |  |  |  |  |  |  |  |  |  |  |
| 0 | Vitvi06g01119\_t001 |  |  |  |  |  |  |  |  |  |  |  |  |
| 0 | Vitvi06g01122\_t001 |  |  |  |  |  |  |  |  |  |  |  |  |
| 0 | Vitvi06g04317\_t001 |  |  |  |  |  |  |  |  |  |  |  |  |
| 0 | Vitvi06g04318\_t001 |  |  |  |  |  |  |  |  |  |  |  |  |
| 0 | Vitvi06g01856\_t001 |  |  |  |  |  |  |  |  |  |  |  |  |
| 0 | Vitvi06g01125\_t001 |  |  |  |  |  |  |  |  |  |  |  |  |
| 0 | Vitvi06g01124\_t001 |  |  |  |  |  |  |  |  |  |  |  |  |
| 0 | Vitvi06g04319\_t001 |  |  |  |  |  |  |  |  |  |  |  |  |
| 0 | Vitvi06g01130\_t001 |  |  |  |  |  |  |  |  |  |  |  |  |
| 0 | Vitvi06g01131\_t001 |  |  |  |  |  |  |  |  |  |  |  |  |
| 0 | Vitvi06g01858\_t001 |  |  |  |  |  |  |  |  |  |  |  |  |
| 0 | Vitvi06g04320\_t001 |  |  |  |  |  |  |  |  |  |  |  |  |
| 0 | Vitvi06g01132\_t001 |  |  |  |  |  |  |  |  |  |  |  |  |
| 0 | Vitvi06g01133\_t001 |  |  |  |  |  |  |  |  |  |  |  |  |
| 0 | Vitvi06g01134\_t001 |  |  |  |  |  |  |  |  |  |  |  |  |
| 0 | Vitvi06g04321\_t001 |  |  |  |  |  |  |  |  |  |  |  |  |
| 0 | Vitvi06g01861\_t001 |  |  |  |  |  |  |  |  |  |  |  |  |
| 0 | Vitvi06g04322\_t001 |  |  |  |  |  |  |  |  |  |  |  |  |
| 0 | Vitvi06g04323\_t001 |  |  |  |  |  |  |  |  |  |  |  |  |
| 0 | Vitvi06g01138\_t001 |  |  |  |  |  |  |  |  |  |  |  |  |
| 0 | Vitvi06g04324\_t001 |  |  |  |  |  |  |  |  |  |  |  |  |
| 0 | Vitvi06g04325\_t001 |  |  |  |  |  |  |  |  |  |  |  |  |
| 0 | Vitvi06g01862\_t001 |  |  |  |  |  |  |  |  |  |  |  |  |
| 0 | Vitvi06g01863\_t001 |  |  |  |  |  |  |  |  |  |  |  |  |
| 0 | Vitvi06g01864\_t001 |  |  |  |  |  |  |  |  |  |  |  |  |
| 0 | Vitvi06g01865\_t001 |  |  |  |  |  |  |  |  |  |  |  |  |
| 0 | Vitvi06g01866\_t001 |  |  |  |  |  |  |  |  |  |  |  |  |
| 0 | Vitvi06g04326\_t001 |  |  |  |  |  |  |  |  |  |  |  |  |
| 0 | Vitvi06g01867\_t001 |  |  |  |  |  |  |  |  |  |  |  |  |
| 0 | Vitvi06g01139\_t001 |  |  |  |  |  |  |  |  |  |  |  |  |
| 0 | Vitvi06g04327\_t001 |  |  |  |  |  |  |  |  |  |  |  |  |
| 0 | Vitvi06g01140\_t001 |  |  |  |  |  |  |  |  |  |  |  |  |
| 0 | Vitvi06g01144\_t001 |  |  |  |  |  |  |  |  |  |  |  |  |
| 0 | Vitvi06g01146\_t001 |  |  |  |  |  |  |  |  |  |  |  |  |
| 0 | Vitvi06g01147\_t001 |  |  |  |  |  |  |  |  |  |  |  |  |
| 0 | Vitvi06g01148\_t002 |  |  |  |  |  |  |  |  |  |  |  |  |
| 0 | Vitvi06g01868\_t001 |  |  |  |  |  |  |  |  |  |  |  |  |
| 0 | Vitvi06g01149\_t001 |  |  |  |  |  |  |  |  |  |  |  |  |
| 0 | Vitvi06g04328\_t001 |  |  |  |  |  |  |  |  |  |  |  |  |
| 0 | Vitvi06g01152\_t001 |  |  |  |  |  |  |  |  |  |  |  |  |
| 0 | Vitvi06g04329\_t001 |  |  |  |  |  |  |  |  |  |  |  |  |
| 0 | Vitvi06g04330\_t001 |  |  |  |  |  |  |  |  |  |  |  |  |
| 0 | Vitvi06g04331\_t001 |  |  |  |  |  |  |  |  |  |  |  |  |
| 0 | Vitvi06g04332\_t001 |  |  |  |  |  |  |  |  |  |  |  |  |
| 0 | Vitvi06g01869\_t001 |  |  |  |  |  |  |  |  |  |  |  |  |
| 0 | Vitvi06g01870\_t001 |  |  |  |  |  |  |  |  |  |  |  |  |
| 0 | Vitvi06g01871\_t001 |  |  |  |  |  |  |  |  |  |  |  |  |
| 0 | Vitvi06g01161\_t003 |  |  |  |  |  |  |  |  |  |  |  |  |
| 0 | Vitvi06g01872\_t001 |  |  |  |  |  |  |  |  |  |  |  |  |
| 0 | Vitvi06g01162\_t001 |  |  |  |  |  |  |  |  |  |  |  |  |
| 0 | Vitvi06g01163\_t001 |  |  |  |  |  |  |  |  |  |  |  |  |
| 0 | Vitvi06g01164\_t001 |  |  |  |  |  |  |  |  |  |  |  |  |
| 0 | Vitvi06g01873\_t001 |  |  |  |  |  |  |  |  |  |  |  |  |
| 0 | Vitvi06g01165\_t002 |  |  |  |  |  |  |  |  |  |  |  |  |
| 0 | Vitvi06g01166\_t001 |  |  |  |  |  |  |  |  |  |  |  |  |
| 0 | Vitvi06g01877\_t001 |  |  |  |  |  |  |  |  |  |  |  |  |
| 0 | Vitvi06g01878\_t001 |  |  |  |  |  |  |  |  |  |  |  |  |
| 0 | Vitvi06g01167\_t001 |  |  |  |  |  |  |  |  |  |  |  |  |
| 0 | Vitvi06g04333\_t001 |  |  |  |  |  |  |  |  |  |  |  |  |
| 0 | Vitvi06g04334\_t001 |  |  |  |  |  |  |  |  |  |  |  |  |
| 0 | Vitvi06g01168\_t001 |  |  |  |  |  |  |  |  |  |  |  |  |
| 0 | Vitvi06g01169\_t002 |  |  |  |  |  |  |  |  |  |  |  |  |
| 0 | Vitvi06g01172\_t001 |  |  |  |  |  |  |  |  |  |  |  |  |
| 0 | Vitvi06g01881\_t001 |  |  |  |  |  |  |  |  |  |  |  |  |
| 0 | Vitvi06g04335\_t001 |  |  |  |  |  |  |  |  |  |  |  |  |
| 0 | Vitvi06g04336\_t001 |  |  |  |  |  |  |  |  |  |  |  |  |
| 0 | Vitvi06g01175\_t001 |  |  |  |  |  |  |  |  |  |  |  |  |
| 0 | Vitvi06g04337\_t001 |  |  |  |  |  |  |  |  |  |  |  |  |
| 0 | Vitvi06g04338\_t001 |  |  |  |  |  |  |  |  |  |  |  |  |
| 0 | Vitvi06g04339\_t001 |  |  |  |  |  |  |  |  |  |  |  |  |
| 0 | Vitvi06g04340\_t001 |  |  |  |  |  |  |  |  |  |  |  |  |
| 0 | Vitvi06g04341\_t001 |  |  |  |  |  |  |  |  |  |  |  |  |
| 0 | Vitvi06g04342\_t001 |  |  |  |  |  |  |  |  |  |  |  |  |
| 0 | Vitvi06g01887\_t001 |  |  |  |  |  |  |  |  |  |  |  |  |
| 0 | Vitvi06g01180\_t001 |  |  |  |  |  |  |  |  |  |  |  |  |
| 0 | Vitvi06g01890\_t001 |  |  |  |  |  |  |  |  |  |  |  |  |
| 0 | Vitvi06g04343\_t001 |  |  |  |  |  |  |  |  |  |  |  |  |
| 0 | Vitvi06g04344\_t001 |  |  |  |  |  |  |  |  |  |  |  |  |
| 0 | Vitvi06g04345\_t001 |  |  |  |  |  |  |  |  |  |  |  |  |
| 0 | Vitvi06g01190\_t001 |  |  |  |  |  |  |  |  |  |  |  |  |
| 0 | Vitvi06g04346\_t001 |  |  |  |  |  |  |  |  |  |  |  |  |
| 0 | Vitvi06g04347\_t001 |  |  |  |  |  |  |  |  |  |  |  |  |
| 0 | Vitvi06g01195\_t001 |  |  |  |  |  |  |  |  |  |  |  |  |
| 0 | Vitvi06g04348\_t001 |  |  |  |  |  |  |  |  |  |  |  |  |
| 0 | Vitvi06g01197\_t001 |  |  |  |  |  |  |  |  |  |  |  |  |
| 0 | Vitvi06g04349\_t001 |  |  |  |  |  |  |  |  |  |  |  |  |
| 0 | Vitvi06g01201\_t001 |  |  |  |  |  |  |  |  |  |  |  |  |
| 0 | Vitvi06g04350\_t001 |  |  |  |  |  |  |  |  |  |  |  |  |
| 0 | Vitvi06g01203\_t001 |  |  |  |  |  |  |  |  |  |  |  |  |
| 0 | Vitvi06g01205\_t001 |  |  |  |  |  |  |  |  |  |  |  |  |
| 0 | Vitvi06g01207\_t001 |  |  |  |  |  |  |  |  |  |  |  |  |
| 0 | Vitvi06g04351\_t001 |  |  |  |  |  |  |  |  |  |  |  |  |
| 0 | Vitvi06g04352\_t001 |  |  |  |  |  |  |  |  |  |  |  |  |
| 0 | Vitvi06g01211\_t001 |  |  |  |  |  |  |  |  |  |  |  |  |
| 0 | Vitvi06g04353\_t001 |  |  |  |  |  |  |  |  |  |  |  |  |
| 0 | Vitvi06g04354\_t001 |  |  |  |  |  |  |  |  |  |  |  |  |
| 0 | Vitvi06g01212\_t001 |  |  |  |  |  |  |  |  |  |  |  |  |
| 0 | Vitvi06g01214\_t001 |  |  |  |  |  |  |  |  |  |  |  |  |
| 0 | Vitvi06g01215\_t001 |  |  |  |  |  |  |  |  |  |  |  |  |
| 0 | Vitvi06g04355\_t001 |  |  |  |  |  |  |  |  |  |  |  |  |
| 0 | Vitvi06g01216\_t001 |  |  |  |  |  |  |  |  |  |  |  |  |
| 0 | Vitvi06g01217\_t002 |  |  |  |  |  |  |  |  |  |  |  |  |
| 0 | Vitvi06g01218\_t001 |  |  |  |  |  |  |  |  |  |  |  |  |
| 0 | Vitvi06g01220\_t001 |  |  |  |  |  |  |  |  |  |  |  |  |
| 0 | Vitvi06g04356\_t001 |  |  |  |  |  |  |  |  |  |  |  |  |
| 0 | Vitvi06g01222\_t001 |  |  |  |  |  |  |  |  |  |  |  |  |
| 0 | Vitvi06g04357\_t001 |  |  |  |  |  |  |  |  |  |  |  |  |
| 0 | Vitvi06g04358\_t001 |  |  |  |  |  |  |  |  |  |  |  |  |
| 0 | Vitvi06g04359\_t001 |  |  |  |  |  |  |  |  |  |  |  |  |
| 0 | Vitvi06g01225\_t001 |  |  |  |  |  |  |  |  |  |  |  |  |
| 0 | Vitvi06g04360\_t001 |  |  |  |  |  |  |  |  |  |  |  |  |
| 0 | Vitvi06g01226\_t001 |  |  |  |  |  |  |  |  |  |  |  |  |
| 0 | Vitvi06g01227\_t001 |  |  |  |  |  |  |  |  |  |  |  |  |
| 0 | Vitvi06g01231\_t001 |  |  |  |  |  |  |  |  |  |  |  |  |
| 0 | Vitvi06g01232\_t001 |  |  |  |  |  |  |  |  |  |  |  |  |
| 0 | Vitvi06g01233\_t001 |  |  |  |  |  |  |  |  |  |  |  |  |
| 0 | Vitvi06g04361\_t001 |  |  |  |  |  |  |  |  |  |  |  |  |
| 0 | Vitvi06g01236\_t001 |  |  |  |  |  |  |  |  |  |  |  |  |
| 0 | Vitvi06g01237\_t001 |  |  |  |  |  |  |  |  |  |  |  |  |
| 0 | Vitvi06g01238\_t001 |  |  |  |  |  |  |  |  |  |  |  |  |
| 0 | Vitvi06g01239\_t001 |  |  |  |  |  |  |  |  |  |  |  |  |
| 0 | Vitvi06g01240\_t001 |  |  |  |  |  |  |  |  |  |  |  |  |
| 0 | Vitvi06g04362\_t001 |  |  |  |  |  |  |  |  |  |  |  |  |
| 0 | Vitvi06g04363\_t001 |  |  |  |  |  |  |  |  |  |  |  |  |
| 0 | Vitvi06g04364\_t001 |  |  |  |  |  |  |  |  |  |  |  |  |
| 0 | Vitvi06g01241\_t001 |  |  |  |  |  |  |  |  |  |  |  |  |
| 0 | Vitvi06g04365\_t001 |  |  |  |  |  |  |  |  |  |  |  |  |
| 0 | Vitvi06g01242\_t001 |  |  |  |  |  |  |  |  |  |  |  |  |
| 0 | Vitvi06g01243\_t001 |  |  |  |  |  |  |  |  |  |  |  |  |
| 0 | Vitvi06g01244\_t001 |  |  |  |  |  |  |  |  |  |  |  |  |
| 0 | Vitvi06g01907\_t001 |  |  |  |  |  |  |  |  |  |  |  |  |
| 0 | Vitvi06g04366\_t001 |  |  |  |  |  |  |  |  |  |  |  |  |
| 0 | Vitvi06g01245\_t001 |  |  |  |  |  |  |  |  |  |  |  |  |
| 0 | Vitvi06g04367\_t001 |  |  |  |  |  |  |  |  |  |  |  |  |
| 0 | Vitvi06g01246\_t001 |  |  |  |  |  |  |  |  |  |  |  |  |
| 0 | Vitvi06g01248\_t001 |  |  |  |  |  |  |  |  |  |  |  |  |
| 0 | Vitvi06g01249\_t001 |  |  |  |  |  |  |  |  |  |  |  |  |
| 0 | Vitvi06g01909\_t001 |  |  |  |  |  |  |  |  |  |  |  |  |
| 0 | Vitvi06g01910\_t001 |  |  |  |  |  |  |  |  |  |  |  |  |
| 0 | Vitvi06g01911\_t001 |  |  |  |  |  |  |  |  |  |  |  |  |
| 0 | Vitvi06g04368\_t001 |  |  |  |  |  |  |  |  |  |  |  |  |
| 0 | Vitvi06g01250\_t001 |  |  |  |  |  |  |  |  |  |  |  |  |
| 0 | Vitvi06g04369\_t001 |  |  |  |  |  |  |  |  |  |  |  |  |
| 0 | Vitvi06g04370\_t001 |  |  |  |  |  |  |  |  |  |  |  |  |
| 0 | Vitvi06g01251\_t001 |  |  |  |  |  |  |  |  |  |  |  |  |
| 0 | Vitvi06g01252\_t001 |  |  |  |  |  |  |  |  |  |  |  |  |
| 0 | Vitvi06g01253\_t001 |  |  |  |  |  |  |  |  |  |  |  |  |
| 0 | Vitvi06g01254\_t001 |  |  |  |  |  |  |  |  |  |  |  |  |
| 0 | Vitvi06g04371\_t001 |  |  |  |  |  |  |  |  |  |  |  |  |
| 0 | Vitvi06g01255\_t001 |  |  |  |  |  |  |  |  |  |  |  |  |
| 0 | Vitvi06g04372\_t001 |  |  |  |  |  |  |  |  |  |  |  |  |
| 0 | Vitvi06g04373\_t001 |  |  |  |  |  |  |  |  |  |  |  |  |
| 0 | Vitvi06g04374\_t001 |  |  |  |  |  |  |  |  |  |  |  |  |
| 0 | Vitvi06g04375\_t001 |  |  |  |  |  |  |  |  |  |  |  |  |
| 0 | Vitvi06g04376\_t001 |  |  |  |  |  |  |  |  |  |  |  |  |
| 0 | Vitvi06g04377\_t001 |  |  |  |  |  |  |  |  |  |  |  |  |
| 0 | Vitvi06g04378\_t001 |  |  |  |  |  |  |  |  |  |  |  |  |
| 0 | Vitvi06g01913\_t001 |  |  |  |  |  |  |  |  |  |  |  |  |
| 0 | Vitvi06g04379\_t001 |  |  |  |  |  |  |  |  |  |  |  |  |
| 0 | Vitvi06g01259\_t001 |  |  |  |  |  |  |  |  |  |  |  |  |
| 0 | Vitvi06g04380\_t001 |  |  |  |  |  |  |  |  |  |  |  |  |
| 0 | Vitvi06g04381\_t001 |  |  |  |  |  |  |  |  |  |  |  |  |
| 0 | Vitvi06g04382\_t001 |  |  |  |  |  |  |  |  |  |  |  |  |
| 0 | Vitvi06g01260\_t001 |  |  |  |  |  |  |  |  |  |  |  |  |
| 0 | Vitvi06g04383\_t001 |  |  |  |  |  |  |  |  |  |  |  |  |
| 0 | Vitvi06g01261\_t002 |  |  |  |  |  |  |  |  |  |  |  |  |
| 0 | Vitvi06g01262\_t002 |  |  |  |  |  |  |  |  |  |  |  |  |
| 0 | Vitvi06g04384\_t001 |  |  |  |  |  |  |  |  |  |  |  |  |
| 0 | Vitvi06g01263\_t001 |  |  |  |  |  |  |  |  |  |  |  |  |
| 0 | Vitvi06g04385\_t001 |  |  |  |  |  |  |  |  |  |  |  |  |
| 0 | Vitvi06g01264\_t001 |  |  |  |  |  |  |  |  |  |  |  |  |
| 0 | Vitvi06g01265\_t001 |  |  |  |  |  |  |  |  |  |  |  |  |
| 0 | Vitvi06g01266\_t002 |  |  |  |  |  |  |  |  |  |  |  |  |
| 0 | Vitvi06g04386\_t001 |  |  |  |  |  |  |  |  |  |  |  |  |
| 0 | Vitvi06g01267\_t001 |  |  |  |  |  |  |  |  |  |  |  |  |
| 0 | Vitvi06g01268\_t001 |  |  |  |  |  |  |  |  |  |  |  |  |
| 0 | Vitvi06g01269\_t001 |  |  |  |  |  |  |  |  |  |  |  |  |
| 0 | Vitvi06g01270\_t001 |  |  |  |  |  |  |  |  |  |  |  |  |
| 0 | Vitvi06g01271\_t001 |  |  |  |  |  |  |  |  |  |  |  |  |
| 0 | Vitvi06g01272\_t001 |  |  |  |  |  |  |  |  |  |  |  |  |
| 1 | Vitvi06g01273\_t001 |  | Vitvi08g01023\_t001 |  |  |  |  |  |  |  |  |  |  |
| 1 | Vitvi06g01274\_t001 |  | \| \| |  |  |  |  |  |  |  |  |  |  |
| 1 | Vitvi06g01275\_t001 |  | \| \| |  |  |  |  |  |  |  |  |  |  |
| 1 | Vitvi06g01276\_t001 |  | \| \| |  |  |  |  |  |  |  |  |  |  |
| 1 | Vitvi06g01279\_t001 |  | \| \| |  |  |  |  |  |  |  |  |  |  |
| 1 | Vitvi06g04387\_t001 |  | \| \| |  |  |  |  |  |  |  |  |  |  |
| 1 | Vitvi06g04388\_t001 |  | \| \| |  |  |  |  |  |  |  |  |  |  |
| 1 | Vitvi06g04389\_t001 |  | \| \| |  |  |  |  |  |  |  |  |  |  |
| 1 | Vitvi06g04390\_t001 |  | \| \| |  |  |  |  |  |  |  |  |  |  |
| 1 | Vitvi06g04391\_t001 |  | \| \| |  |  |  |  |  |  |  |  |  |  |
| 1 | Vitvi06g01917\_t001 |  | \| \| |  |  |  |  |  |  |  |  |  |  |
| 1 | Vitvi06g01918\_t001 |  | \| \| |  |  |  |  |  |  |  |  |  |  |
| 1 | Vitvi06g04392\_t001 |  | \| \| |  |  |  |  |  |  |  |  |  |  |
| 1 | Vitvi06g01281\_t001 |  | \| \| |  |  |  |  |  |  |  |  |  |  |
| 1 | Vitvi06g04393\_t001 |  | \| \| |  |  |  |  |  |  |  |  |  |  |
| 1 | Vitvi06g04394\_t001 |  | \| \| |  |  |  |  |  |  |  |  |  |  |
| 1 | Vitvi06g01282\_t001 |  | \| \| |  |  |  |  |  |  |  |  |  |  |
| 1 | Vitvi06g01283\_t001 |  | \| \| |  |  |  |  |  |  |  |  |  |  |
| 1 | Vitvi06g04395\_t001 |  | \| \| |  |  |  |  |  |  |  |  |  |  |
| 1 | Vitvi06g01285\_t001 |  | \| \| |  |  |  |  |  |  |  |  |  |  |
| 1 | Vitvi06g01286\_t001 |  | \| \| |  |  |  |  |  |  |  |  |  |  |
| 1 | Vitvi06g01288\_t001 |  | \| \| |  |  |  |  |  |  |  |  |  |  |
| 1 | Vitvi06g01289\_t001 |  | Vitvi08g01013\_t001 |  |  |  |  |  |  |  |  |  |  |
| 1 | Vitvi06g01290\_t001 |  | \| \| |  |  |  |  |  |  |  |  |  |  |
| 1 | Vitvi06g01293\_t001 |  | \| \| |  |  |  |  |  |  |  |  |  |  |
| 1 | Vitvi06g04396\_t001 |  | \| \| |  |  |  |  |  |  |  |  |  |  |
| 1 | Vitvi06g01295\_t001 |  | \| \| |  |  |  |  |  |  |  |  |  |  |
| 1 | Vitvi06g01297\_t001 |  | Vitvi08g01012\_t002 |  |  |  |  |  |  |  |  |  |  |
| 1 | Vitvi06g04397\_t001 |  | \| \| |  |  |  |  |  |  |  |  |  |  |
| 1 | Vitvi06g01298\_t001 |  | \| \| |  |  |  |  |  |  |  |  |  |  |
| 1 | Vitvi06g01299\_t001 |  | Vitvi08g01004\_t001 |  |  |  |  |  |  |  |  |  |  |
| 1 | Vitvi06g04398\_t001 |  | \| \| |  |  |  |  |  |  |  |  |  |  |
| 1 | Vitvi06g01301\_t001 |  | \| \| |  |  |  |  |  |  |  |  |  |  |
| 1 | Vitvi06g01302\_t001 |  | \| \| |  |  |  |  |  |  |  |  |  |  |
| 1 | Vitvi06g04399\_t001 |  | \| \| |  |  |  |  |  |  |  |  |  |  |
| 1 | Vitvi06g01303\_t001 |  | Vitvi08g01002\_t001 |  |  |  |  |  |  |  |  |  |  |
| 1 | Vitvi06g01306\_t001 |  | \| \| |  |  |  |  |  |  |  |  |  |  |
| 1 | Vitvi06g01307\_t001 |  | Vitvi08g01001\_t001 |  |  |  |  |  |  |  |  |  |  |
| 1 | Vitvi06g01309\_t001 |  | \| \| |  |  |  |  |  |  |  |  |  |  |
| 1 | Vitvi06g01310\_t001 |  | Vitvi08g02133\_t001 |  |  |  |  |  |  |  |  |  |  |
| 1 | Vitvi06g01312\_t001 |  | \| \| |  |  |  |  |  |  |  |  |  |  |
| 1 | Vitvi06g01313\_t001 |  | \| \| |  |  |  |  |  |  |  |  |  |  |
| 1 | Vitvi06g04400\_t001 |  | \| \| |  |  |  |  |  |  |  |  |  |  |
| 1 | Vitvi06g01314\_t001 |  | \| \| |  |  |  |  |  |  |  |  |  |  |
| 1 | Vitvi06g04401\_t001 |  | \| \| |  |  |  |  |  |  |  |  |  |  |
| 1 | Vitvi06g01315\_t001 |  | Vitvi08g00992\_t001 |  |  |  |  |  |  |  |  |  |  |
| 0 | Vitvi06g04402\_t001 |  |  |  |  |  |  |  |  |  |  |  |  |
| 1 | Vitvi06g01318\_t001 |  | Vitvi08g04351\_t001 |  |  |  |  |  |  |  |  |  |  |
| 1 | Vitvi06g01320\_t002 |  | \| \| |  |  |  |  |  |  |  |  |  |  |
| 1 | Vitvi06g01321\_t001 |  | Vitvi08g01623\_t001 |  |  |  |  |  |  |  |  |  |  |
| 1 | Vitvi06g04403\_t001 |  | \| \| |  |  |  |  |  |  |  |  |  |  |
| 1 | Vitvi06g01322\_t001 |  | \| \| |  |  |  |  |  |  |  |  |  |  |
| 1 | Vitvi06g01323\_t001 |  | \| \| |  |  |  |  |  |  |  |  |  |  |
| 1 | Vitvi06g01324\_t002 |  | \| \| |  |  |  |  |  |  |  |  |  |  |
| 1 | Vitvi06g04404\_t001 |  | \| \| |  |  |  |  |  |  |  |  |  |  |
| 1 | Vitvi06g01325\_t001 |  | Vitvi08g04349\_t001 |  |  |  |  |  |  |  |  |  |  |
| 1 | Vitvi06g01327\_t001 |  | \| \| |  |  |  |  |  |  |  |  |  |  |
| 1 | Vitvi06g01924\_t001 |  | \| \| |  |  |  |  |  |  |  |  |  |  |
| 1 | Vitvi06g04405\_t001 |  | \| \| |  |  |  |  |  |  |  |  |  |  |
| 1 | Vitvi06g01328\_t002 |  | \| \| |  |  |  |  |  |  |  |  |  |  |
| 1 | Vitvi06g01329\_t001 |  | Vitvi08g01617\_t001 |  |  |  |  |  |  |  |  |  |  |
| 1 | Vitvi06g01330\_t001 |  | \| \| |  |  |  |  |  |  |  |  |  |  |
| 1 | Vitvi06g01331\_t001 |  | \| \| |  |  |  |  |  |  |  |  |  |  |
| 1 | Vitvi06g04406\_t001 |  | \| \| |  |  |  |  |  |  |  |  |  |  |
| 1 | Vitvi06g01334\_t001 |  | Vitvi08g01611\_t001 |  |  |  |  |  |  |  |  |  |  |
| 1 | Vitvi06g01927\_t001 |  | \| \| |  |  |  |  |  |  |  |  |  |  |
| 1 | Vitvi06g01335\_t001 |  | \| \| |  |  |  |  |  |  |  |  |  |  |
| 1 | Vitvi06g01928\_t001 |  | \| \| |  |  |  |  |  |  |  |  |  |  |
| 1 | Vitvi06g01929\_t001 |  | \| \| |  |  |  |  |  |  |  |  |  |  |
| 1 | Vitvi06g01930\_t003 |  | \| \| |  |  |  |  |  |  |  |  |  |  |
| 1 | Vitvi06g01340\_t001 |  | \| \| |  |  |  |  |  |  |  |  |  |  |
| 1 | Vitvi06g01341\_t001 |  | \| \| |  |  |  |  |  |  |  |  |  |  |
| 1 | Vitvi06g01343\_t001 |  | \| \| |  |  |  |  |  |  |  |  |  |  |
| 1 | Vitvi06g04407\_t001 |  | \| \| |  |  |  |  |  |  |  |  |  |  |
| 1 | Vitvi06g01344\_t001 |  | Vitvi08g01603\_t004 |  |  |  |  |  |  |  |  |  |  |
| 1 | Vitvi06g01345\_t001 |  | \| \| |  |  |  |  |  |  |  |  |  |  |
| 1 | Vitvi06g01346\_t001 |  | Vitvi08g01602\_t001 |  |  |  |  |  |  |  |  |  |  |
| 1 | Vitvi06g04408\_t001 |  | \| \| |  |  |  |  |  |  |  |  |  |  |
| 1 | Vitvi06g01348\_t001 |  | \| \| |  |  |  |  |  |  |  |  |  |  |
| 1 | Vitvi06g01349\_t001 |  | \| \| |  |  |  |  |  |  |  |  |  |  |
| 1 | Vitvi06g01350\_t001 |  | Vitvi08g01600\_t001 |  |  |  |  |  |  |  |  |  |  |
| 1 | Vitvi06g04409\_t001 |  | \| \| |  |  |  |  |  |  |  |  |  |  |
| 1 | Vitvi06g01351\_t001 |  | \| \| |  |  |  |  |  |  |  |  |  |  |
| 1 | Vitvi06g01353\_t001 |  | \| \| |  |  |  |  |  |  |  |  |  |  |
| 1 | Vitvi06g01354\_t001 |  | \| \| |  |  |  |  |  |  |  |  |  |  |
| 1 | Vitvi06g04410\_t001 |  | \| \| |  |  |  |  |  |  |  |  |  |  |
| 1 | Vitvi06g04411\_t001 |  | \| \| |  |  |  |  |  |  |  |  |  |  |
| 1 | Vitvi06g01933\_t001 |  | \| \| |  |  |  |  |  |  |  |  |  |  |
| 1 | Vitvi06g04412\_t001 |  | \| \| |  |  |  |  |  |  |  |  |  |  |
| 1 | Vitvi06g01934\_t001 |  | \| \| |  |  |  |  |  |  |  |  |  |  |
| 1 | Vitvi06g01357\_t001 |  | \| \| |  |  |  |  |  |  |  |  |  |  |
| 1 | Vitvi06g04413\_t001 |  | \| \| |  |  |  |  |  |  |  |  |  |  |
| 1 | Vitvi06g01358\_t001 |  | \| \| |  |  |  |  |  |  |  |  |  |  |
| 1 | Vitvi06g01359\_t001 |  | \| \| |  |  |  |  |  |  |  |  |  |  |
| 1 | Vitvi06g04414\_t001 |  | \| \| |  |  |  |  |  |  |  |  |  |  |
| 1 | Vitvi06g01360\_t001 |  | \| \| |  |  |  |  |  |  |  |  |  |  |
| 1 | Vitvi06g04415\_t001 |  | \| \| |  |  |  |  |  |  |  |  |  |  |
| 1 | Vitvi06g01361\_t001 |  | Vitvi08g01579\_t002 |  |  |  |  |  |  |  |  |  |  |
| 1 | Vitvi06g01362\_t001 |  | Vitvi08g01569\_t001 |  |  |  |  |  |  |  |  |  |  |
| 1 | Vitvi06g04416\_t001 |  | \| \| |  |  |  |  |  |  |  |  |  |  |
| 1 | Vitvi06g04417\_t001 |  | \| \| |  |  |  |  |  |  |  |  |  |  |
| 1 | Vitvi06g04418\_t001 |  | \| \| |  |  |  |  |  |  |  |  |  |  |
| 1 | Vitvi06g04419\_t001 |  | \| \| |  |  |  |  |  |  |  |  |  |  |
| 1 | Vitvi06g04420\_t001 |  | \| \| |  |  |  |  |  |  |  |  |  |  |
| 1 | Vitvi06g01364\_t001 |  | \| \| |  |  |  |  |  |  |  |  |  |  |
| 1 | Vitvi06g01365\_t001 |  | \| \| |  |  |  |  |  |  |  |  |  |  |
| 1 | Vitvi06g01367\_t001 |  | \| \| |  |  |  |  |  |  |  |  |  |  |
| 1 | Vitvi06g01935\_t001 |  | \| \| |  |  |  |  |  |  |  |  |  |  |
| 1 | Vitvi06g01936\_t001 |  | \| \| |  |  |  |  |  |  |  |  |  |  |
| 1 | Vitvi06g04421\_t001 |  | \| \| |  |  |  |  |  |  |  |  |  |  |
| 1 | Vitvi06g04422\_t001 |  | \| \| |  |  |  |  |  |  |  |  |  |  |
| 1 | Vitvi06g01374\_t001 |  | \| \| |  |  |  |  |  |  |  |  |  |  |
| 1 | Vitvi06g01937\_t001 |  | \| \| |  |  |  |  |  |  |  |  |  |  |
| 1 | Vitvi06g01375\_t001 |  | \| \| |  |  |  |  |  |  |  |  |  |  |
| 1 | Vitvi06g01376\_t001 |  | \| \| |  |  |  |  |  |  |  |  |  |  |
| 1 | Vitvi06g01377\_t001 |  | Vitvi08g01562\_t001 |  |  |  |  |  |  |  |  |  |  |
| 1 | Vitvi06g01378\_t001 |  | Vitvi08g01559\_t001 |  |  |  |  |  |  |  |  |  |  |
| 1 | Vitvi06g01380\_t001 |  | \| \| |  |  |  |  |  |  |  |  |  |  |
| 1 | Vitvi06g01383\_t003 |  | \| \| |  |  |  |  |  |  |  |  |  |  |
| 1 | Vitvi06g01384\_t001 |  | \| \| |  |  |  |  |  |  |  |  |  |  |
| 1 | Vitvi06g01385\_t001 |  | \| \| |  |  |  |  |  |  |  |  |  |  |
| 1 | Vitvi06g04423\_t001 |  | \| \| |  |  |  |  |  |  |  |  |  |  |
| 1 | Vitvi06g01386\_t001 |  | \| \| |  |  |  |  |  |  |  |  |  |  |
| 1 | Vitvi06g04424\_t001 |  | \| \| |  |  |  |  |  |  |  |  |  |  |
| 1 | Vitvi06g04425\_t001 |  | \| \| |  |  |  |  |  |  |  |  |  |  |
| 1 | Vitvi06g01939\_t001 |  | \| \| |  |  |  |  |  |  |  |  |  |  |
| 1 | Vitvi06g01940\_t001 |  | \| \| |  |  |  |  |  |  |  |  |  |  |
| 1 | Vitvi06g01387\_t001 |  | Vitvi08g01553\_t001 |  |  |  |  |  |  |  |  |  |  |
| 1 | Vitvi06g01388\_t001 |  | \| \| |  |  |  |  |  |  |  |  |  |  |
| 1 | Vitvi06g01389\_t001 |  | \| \| |  |  |  |  |  |  |  |  |  |  |
| 1 | Vitvi06g01942\_t001 |  | \| \| |  |  |  |  |  |  |  |  |  |  |
| 1 | Vitvi06g01943\_t001 |  | \| \| |  |  |  |  |  |  |  |  |  |  |
| 1 | Vitvi06g01391\_t001 |  | \| \| |  |  |  |  |  |  |  |  |  |  |
| 1 | Vitvi06g01392\_t001 |  | \| \| |  |  |  |  |  |  |  |  |  |  |
| 1 | Vitvi06g01394\_t003 |  | \| \| |  |  |  |  |  |  |  |  |  |  |
| 1 | Vitvi06g01395\_t001 |  | \| \| |  |  |  |  |  |  |  |  |  |  |
| 2 | Vitvi06g01944\_t001 |  | \| \| |  | Vitvi08g01525\_t001 |  |  |  |  |  |  |  |  |
| 3 | Vitvi06g01396\_t001 |  | \| \| |  | \| \| |  | Vitvi13g02101\_t001 |  |  |  |  |  |  |
| 3 | Vitvi06g01397\_t001 |  | \| \| |  | \| \| |  | \| \| |  |  |  |  |  |  |
| 3 | Vitvi06g04426\_t001 |  | \| \| |  | \| \| |  | \| \| |  |  |  |  |  |  |
| 3 | Vitvi06g04427\_t001 |  | \| \| |  | \| \| |  | \| \| |  |  |  |  |  |  |
| 3 | Vitvi06g01398\_t001 |  | \| \| |  | Vitvi08g01523\_t002 |  | \| \| |  |  |  |  |  |  |
| 3 | Vitvi06g01399\_t001 |  | \| \| |  | Vitvi08g01518\_t002 |  | \| \| |  |  |  |  |  |  |
| 3 | Vitvi06g01400\_t001 |  | \| \| |  | \| \| |  | \| \| |  |  |  |  |  |  |
| 3 | Vitvi06g01401\_t001 |  | \| \| |  | \| \| |  | \| \| |  |  |  |  |  |  |
| 3 | Vitvi06g01402\_t001 |  | \| \| |  | Vitvi08g01515\_t001 |  | \| \| |  |  |  |  |  |  |
| 3 | Vitvi06g01403\_t001 |  | \| \| |  | \| \| |  | \| \| |  |  |  |  |  |  |
| 3 | Vitvi06g01945\_t001 |  | \| \| |  | Vitvi08g01510\_t001 |  | \| \| |  |  |  |  |  |  |
| 3 | Vitvi06g01946\_t001 |  | \| \| |  | \| \| |  | \| \| |  |  |  |  |  |  |
| 3 | Vitvi06g01404\_t001 |  | \| \| |  | \| \| |  | \| \| |  |  |  |  |  |  |
| 3 | Vitvi06g01947\_t001 |  | \| \| |  | \| \| |  | \| \| |  |  |  |  |  |  |
| 3 | Vitvi06g01406\_t001 |  | Vitvi08g01544\_t001 |  | Vitvi08g01508\_t002 |  | \| \| |  |  |  |  |  |  |
| 2 | Vitvi06g01407\_t001 |  |  |  | \| \| |  | \| \| |  |  |  |  |  |  |
| 2 | Vitvi06g01948\_t001 |  |  |  | \| \| |  | \| \| |  |  |  |  |  |  |
| 2 | Vitvi06g01408\_t001 |  |  |  | Vitvi08g01507\_t001 |  | \| \| |  |  |  |  |  |  |
| 2 | Vitvi06g01409\_t001 |  |  |  | \| \| |  | \| \| |  |  |  |  |  |  |
| 2 | Vitvi06g01410\_t001 |  |  |  | \| \| |  | \| \| |  |  |  |  |  |  |
| 2 | Vitvi06g01411\_t001 |  |  |  | Vitvi08g01501\_t001 |  | \| \| |  |  |  |  |  |  |
| 2 | Vitvi06g01414\_t001 |  |  |  | \| \| |  | \| \| |  |  |  |  |  |  |
| 2 | Vitvi06g01415\_t001 |  |  |  | \| \| |  | \| \| |  |  |  |  |  |  |
| 2 | Vitvi06g01416\_t001 |  |  |  | \| \| |  | Vitvi13g00768\_t001 |  |  |  |  |  |  |
| 2 | Vitvi06g01417\_t001 |  |  |  | \| \| |  | Vitvi13g00772\_t001 |  |  |  |  |  |  |
| 2 | Vitvi06g01418\_t001 |  |  |  | Vitvi08g01499\_t001 |  | Vitvi13g00777\_t001 |  |  |  |  |  |  |
| 2 | Vitvi06g01419\_t001 |  |  |  | \| \| |  | Vitvi13g00778\_t001 |  |  |  |  |  |  |
| 2 | Vitvi06g01420\_t001 |  |  |  | \| \| |  | \| \| |  |  |  |  |  |  |
| 2 | Vitvi06g04428\_t001 |  |  |  | \| \| |  | \| \| |  |  |  |  |  |  |
| 2 | Vitvi06g04429\_t001 |  |  |  | \| \| |  | \| \| |  |  |  |  |  |  |
| 2 | Vitvi06g04430\_t001 |  |  |  | \| \| |  | \| \| |  |  |  |  |  |  |
| 2 | Vitvi06g01949\_t001 |  |  |  | \| \| |  | Vitvi13g00779\_t001 |  |  |  |  |  |  |
| 2 | Vitvi06g01950\_t001 |  |  |  | \| \| |  | \| \| |  |  |  |  |  |  |
| 2 | Vitvi06g04431\_t001 |  |  |  | \| \| |  | \| \| |  |  |  |  |  |  |
| 2 | Vitvi06g04432\_t001 |  |  |  | \| \| |  | \| \| |  |  |  |  |  |  |
| 2 | Vitvi06g01427\_t001 |  |  |  | \| \| |  | Vitvi13g00792\_t001 |  |  |  |  |  |  |
| 2 | Vitvi06g01428\_t001 |  |  |  | \| \| |  | \| \| |  |  |  |  |  |  |
| 2 | Vitvi06g01429\_t001 |  |  |  | \| \| |  | \| \| |  |  |  |  |  |  |
| 2 | Vitvi06g04433\_t001 |  |  |  | \| \| |  | \| \| |  |  |  |  |  |  |
| 2 | Vitvi06g01430\_t001 |  |  |  | \| \| |  | Vitvi13g00793\_t001 |  |  |  |  |  |  |
| 2 | Vitvi06g04434\_t001 |  |  |  | \| \| |  | \| \| |  |  |  |  |  |  |
| 2 | Vitvi06g01433\_t001 |  |  |  | \| \| |  | Vitvi13g00797\_t001 |  |  |  |  |  |  |
| 2 | Vitvi06g01435\_t001 |  |  |  | \| \| |  | \| \| |  |  |  |  |  |  |
| 2 | Vitvi06g01442\_t002 |  |  |  | \| \| |  | \| \| |  |  |  |  |  |  |
| 2 | Vitvi06g01443\_t001 |  |  |  | \| \| |  | \| \| |  |  |  |  |  |  |
| 2 | Vitvi06g01444\_t001 |  |  |  | \| \| |  | \| \| |  |  |  |  |  |  |
| 2 | Vitvi06g01952\_t001 |  |  |  | \| \| |  | \| \| |  |  |  |  |  |  |
| 2 | Vitvi06g01446\_t001 |  |  |  | \| \| |  | \| \| |  |  |  |  |  |  |
| 2 | Vitvi06g01447\_t001 |  |  |  | Vitvi08g01493\_t001 |  | \| \| |  |  |  |  |  |  |
| 2 | Vitvi06g01448\_t001 |  |  |  | \| \| |  | \| \| |  |  |  |  |  |  |
| 2 | Vitvi06g01449\_t001 |  |  |  | \| \| |  | \| \| |  |  |  |  |  |  |
| 2 | Vitvi06g04435\_t001 |  |  |  | \| \| |  | \| \| |  |  |  |  |  |  |
| 2 | Vitvi06g04436\_t001 |  |  |  | \| \| |  | \| \| |  |  |  |  |  |  |
| 2 | Vitvi06g01953\_t001 |  |  |  | \| \| |  | \| \| |  |  |  |  |  |  |
| 2 | Vitvi06g01954\_t001 |  |  |  | \| \| |  | \| \| |  |  |  |  |  |  |
| 2 | Vitvi06g01451\_t001 |  |  |  | \| \| |  | Vitvi13g00802\_t001 |  |  |  |  |  |  |
| 1 | Vitvi06g01452\_t001 |  |  |  | Vitvi08g01485\_t001 |  |  |  |  |  |  |  |  |
| 1 | Vitvi06g01453\_t001 |  |  |  | \| \| |  |  |  |  |  |  |  |  |
| 1 | Vitvi06g01454\_t001 |  |  |  | \| \| |  |  |  |  |  |  |  |  |
| 1 | Vitvi06g01455\_t001 |  |  |  | \| \| |  |  |  |  |  |  |  |  |
| 1 | Vitvi06g01456\_t001 |  |  |  | \| \| |  |  |  |  |  |  |  |  |
| 1 | Vitvi06g01457\_t002 |  |  |  | \| \| |  |  |  |  |  |  |  |  |
| 1 | Vitvi06g01458\_t001 |  |  |  | \| \| |  |  |  |  |  |  |  |  |
| 1 | Vitvi06g01459\_t001 |  |  |  | \| \| |  |  |  |  |  |  |  |  |
| 1 | Vitvi06g01460\_t001 |  |  |  | \| \| |  |  |  |  |  |  |  |  |
| 1 | Vitvi06g01462\_t001 |  |  |  | \| \| |  |  |  |  |  |  |  |  |
| 1 | Vitvi06g04437\_t001 |  |  |  | \| \| |  |  |  |  |  |  |  |  |
| 1 | Vitvi06g01464\_t001 |  |  |  | \| \| |  |  |  |  |  |  |  |  |
| 1 | Vitvi06g01465\_t001 |  |  |  | \| \| |  |  |  |  |  |  |  |  |
| 1 | Vitvi06g01958\_t001 |  |  |  | \| \| |  |  |  |  |  |  |  |  |
| 1 | Vitvi06g01467\_t001 |  |  |  | \| \| |  |  |  |  |  |  |  |  |
| 1 | Vitvi06g01468\_t001 |  |  |  | \| \| |  |  |  |  |  |  |  |  |
| 1 | Vitvi06g01469\_t001 |  |  |  | \| \| |  |  |  |  |  |  |  |  |
| 1 | Vitvi06g01470\_t001 |  |  |  | \| \| |  |  |  |  |  |  |  |  |
| 1 | Vitvi06g01471\_t001 |  |  |  | \| \| |  |  |  |  |  |  |  |  |
| 1 | Vitvi06g01472\_t001 |  |  |  | \| \| |  |  |  |  |  |  |  |  |
| 1 | Vitvi06g01473\_t001 |  |  |  | Vitvi08g01473\_t001 |  |  |  |  |  |  |  |  |
| 1 | Vitvi06g04438\_t001 |  |  |  | Vitvi08g01472\_t001 |  |  |  |  |  |  |  |  |
| 1 | Vitvi06g01476\_t001 |  |  |  | \| \| |  |  |  |  |  |  |  |  |
| 1 | Vitvi06g01477\_t001 |  |  |  | \| \| |  |  |  |  |  |  |  |  |
| 1 | Vitvi06g01478\_t001 |  |  |  | Vitvi08g01470\_t001 |  |  |  |  |  |  |  |  |
| 1 | Vitvi06g04439\_t001 |  |  |  | \| \| |  |  |  |  |  |  |  |  |
| 1 | Vitvi06g01479\_t001 |  |  |  | \| \| |  |  |  |  |  |  |  |  |
| 1 | Vitvi06g01480\_t001 |  |  |  | \| \| |  |  |  |  |  |  |  |  |
| 1 | Vitvi06g01481\_t001 |  |  |  | Vitvi08g01469\_t001 |  |  |  |  |  |  |  |  |
| 1 | Vitvi06g04440\_t001 |  |  |  | \| \| |  |  |  |  |  |  |  |  |
| 1 | Vitvi06g04441\_t001 |  |  |  | \| \| |  |  |  |  |  |  |  |  |
| 1 | Vitvi06g01485\_t001 |  |  |  | \| \| |  |  |  |  |  |  |  |  |
| 1 | Vitvi06g01959\_t001 |  |  |  | Vitvi08g01465\_t001 |  |  |  |  |  |  |  |  |
| 1 | Vitvi06g01486\_t002 |  |  |  | Vitvi08g01462\_t001 |  |  |  |  |  |  |  |  |
| 1 | Vitvi06g01487\_t002 |  |  |  | \| \| |  |  |  |  |  |  |  |  |
| 1 | Vitvi06g01489\_t001 |  |  |  | \| \| |  |  |  |  |  |  |  |  |
| 1 | Vitvi06g01491\_t001 |  |  |  | \| \| |  |  |  |  |  |  |  |  |
| 1 | Vitvi06g01961\_t001 |  |  |  | \| \| |  |  |  |  |  |  |  |  |
| 1 | Vitvi06g01492\_t001 |  |  |  | \| \| |  |  |  |  |  |  |  |  |
| 1 | Vitvi06g01493\_t003 |  |  |  | Vitvi08g01450\_t002 |  |  |  |  |  |  |  |  |
| 1 | Vitvi06g01498\_t001 |  |  |  | \| \| |  |  |  |  |  |  |  |  |
| 1 | Vitvi06g01499\_t001 |  |  |  | \| \| |  |  |  |  |  |  |  |  |
| 1 | Vitvi06g04442\_t001 |  |  |  | \| \| |  |  |  |  |  |  |  |  |
| 1 | Vitvi06g04443\_t001 |  |  |  | \| \| |  |  |  |  |  |  |  |  |
| 1 | Vitvi06g01962\_t003 |  |  |  | \| \| |  |  |  |  |  |  |  |  |
| 1 | Vitvi06g01501\_t001 |  |  |  | \| \| |  |  |  |  |  |  |  |  |
| 1 | Vitvi06g01502\_t001 |  |  |  | \| \| |  |  |  |  |  |  |  |  |
| 1 | Vitvi06g01503\_t001 |  |  |  | \| \| |  |  |  |  |  |  |  |  |
| 1 | Vitvi06g01504\_t001 |  |  |  | Vitvi08g01442\_t001 |  |  |  |  |  |  |  |  |
| 1 | Vitvi06g01505\_t001 |  |  |  | \| \| |  |  |  |  |  |  |  |  |
| 1 | Vitvi06g01963\_t001 |  |  |  | \| \| |  |  |  |  |  |  |  |  |
| 1 | Vitvi06g01506\_t001 |  |  |  | \| \| |  |  |  |  |  |  |  |  |
| 1 | Vitvi06g04444\_t001 |  |  |  | \| \| |  |  |  |  |  |  |  |  |
| 1 | Vitvi06g01965\_t001 |  |  |  | \| \| |  |  |  |  |  |  |  |  |
| 1 | Vitvi06g01966\_t001 |  |  |  | \| \| |  |  |  |  |  |  |  |  |
| 1 | Vitvi06g01507\_t001 |  |  |  | \| \| |  |  |  |  |  |  |  |  |
| 1 | Vitvi06g01508\_t001 |  |  |  | \| \| |  |  |  |  |  |  |  |  |
| 1 | Vitvi06g01509\_t001 |  |  |  | \| \| |  |  |  |  |  |  |  |  |
| 1 | Vitvi06g01968\_t001 |  |  |  | \| \| |  |  |  |  |  |  |  |  |
| 1 | Vitvi06g01510\_t001 |  |  |  | \| \| |  |  |  |  |  |  |  |  |
| 1 | Vitvi06g01969\_t001 |  |  |  | \| \| |  |  |  |  |  |  |  |  |
| 1 | Vitvi06g01970\_t001 |  |  |  | \| \| |  |  |  |  |  |  |  |  |
| 1 | Vitvi06g01971\_t001 |  |  |  | \| \| |  |  |  |  |  |  |  |  |
| 1 | Vitvi06g01972\_t001 |  |  |  | \| \| |  |  |  |  |  |  |  |  |
| 1 | Vitvi06g04445\_t001 |  |  |  | Vitvi08g04305\_t001 |  |  |  |  |  |  |  |  |
| 1 | Vitvi06g01512\_t001 |  |  |  | Vitvi08g01427\_t001 |  |  |  |  |  |  |  |  |
| 1 | Vitvi06g01513\_t001 |  |  |  | \| \| |  |  |  |  |  |  |  |  |
| 1 | Vitvi16g02097\_t001 |  |  |  | \| \| |  |  |  |  |  |  |  |  |
| 1 | Vitvi16g02096\_t001 |  |  |  | \| \| |  |  |  |  |  |  |  |  |
| 1 | Vitvi16g02094\_t001 |  |  |  | \| \| |  |  |  |  |  |  |  |  |
| 1 | Vitvi16g02093\_t001 |  |  |  | \| \| |  |  |  |  |  |  |  |  |
| 1 | Vitvi06g01515\_t001 |  |  |  | Vitvi08g01426\_t001 |  |  |  |  |  |  |  |  |
| 1 | Vitvi06g01516\_t001 |  |  |  | \| \| |  |  |  |  |  |  |  |  |
| 1 | Vitvi06g01517\_t001 |  |  |  | \| \| |  |  |  |  |  |  |  |  |
| 1 | Vitvi06g01518\_t001 |  |  |  | \| \| |  |  |  |  |  |  |  |  |
| 1 | Vitvi06g01520\_t001 |  |  |  | \| \| |  |  |  |  |  |  |  |  |
| 1 | Vitvi06g01521\_t001 |  |  |  | Vitvi08g01419\_t001 |  |  |  |  |  |  |  |  |
| 1 | Vitvi06g01522\_t001 |  |  |  | Vitvi08g01418\_t001 |  |  |  |  |  |  |  |  |
| 1 | Vitvi06g01523\_t001 |  |  |  | \| \| |  |  |  |  |  |  |  |  |
| 1 | Vitvi06g01524\_t002 |  |  |  | Vitvi08g01417\_t001 |  |  |  |  |  |  |  |  |
| 1 | Vitvi06g01974\_t001 |  |  |  | Vitvi08g02257\_t001 |  |  |  |  |  |  |  |  |
| 1 | Vitvi06g01526\_t001 |  |  |  | \| \| |  |  |  |  |  |  |  |  |
| 1 | Vitvi06g01527\_t001 |  |  |  | \| \| |  |  |  |  |  |  |  |  |
| 1 | Vitvi06g01529\_t002 |  |  |  | \| \| |  |  |  |  |  |  |  |  |
| 1 | Vitvi06g01530\_t001 |  |  |  | Vitvi08g01416\_t001 |  |  |  |  |  |  |  |  |
| 1 | Vitvi06g01531\_t001 |  |  |  | \| \| |  |  |  |  |  |  |  |  |
| 1 | Vitvi06g01532\_t001 |  |  |  | Vitvi08g01410\_t002 |  |  |  |  |  |  |  |  |
| 1 | Vitvi06g01533\_t001 |  |  |  | \| \| |  |  |  |  |  |  |  |  |
| 1 | Vitvi06g01534\_t001 |  |  |  | \| \| |  |  |  |  |  |  |  |  |
| 1 | Vitvi06g01975\_t001 |  |  |  | \| \| |  |  |  |  |  |  |  |  |
| 1 | Vitvi06g01536\_t001 |  |  |  | \| \| |  |  |  |  |  |  |  |  |
| 1 | Vitvi06g01537\_t001 |  |  |  | Vitvi08g02255\_t001 |  |  |  |  |  |  |  |  |
| 1 | Vitvi06g01538\_t001 |  |  |  | \| \| |  |  |  |  |  |  |  |  |
| 1 | Vitvi06g01539\_t001 |  |  |  | \| \| |  |  |  |  |  |  |  |  |
| 1 | Vitvi06g04446\_t001 |  |  |  | \| \| |  |  |  |  |  |  |  |  |
| 1 | Vitvi06g01542\_t001 |  |  |  | \| \| |  |  |  |  |  |  |  |  |
| 1 | Vitvi06g01543\_t001 |  |  |  | Vitvi08g01395\_t001 |  |  |  |  |  |  |  |  |
| 1 | Vitvi06g01545\_t001 |  |  |  | Vitvi08g01394\_t001 |  |  |  |  |  |  |  |  |
| 1 | Vitvi06g01546\_t001 |  |  |  | Vitvi08g01393\_t001 |  |  |  |  |  |  |  |  |
| 1 | Vitvi06g01548\_t001 |  |  |  | Vitvi08g01392\_t001 |  |  |  |  |  |  |  |  |
| 1 | Vitvi06g01549\_t001 |  |  |  | Vitvi08g01391\_t001 |  |  |  |  |  |  |  |  |
| 1 | Vitvi06g01551\_t001 |  |  |  | \| \| |  |  |  |  |  |  |  |  |
| 1 | Vitvi06g01552\_t001 |  |  |  | Vitvi08g01388\_t001 |  |  |  |  |  |  |  |  |
| 1 | Vitvi06g01553\_t001 |  |  |  | \| \| |  |  |  |  |  |  |  |  |
| 1 | Vitvi06g01555\_t001 |  |  |  | \| \| |  |  |  |  |  |  |  |  |
| 1 | Vitvi06g01556\_t001 |  |  |  | Vitvi08g04284\_t001 |  |  |  |  |  |  |  |  |
| 1 | Vitvi06g04447\_t001 |  |  |  | \| \| |  |  |  |  |  |  |  |  |
| 1 | Vitvi06g01557\_t001 |  |  |  | \| \| |  |  |  |  |  |  |  |  |
| 1 | Vitvi06g01559\_t001 |  |  |  | Vitvi08g01376\_t001 |  |  |  |  |  |  |  |  |
| 0 | Vitvi06g01560\_t001 |  |  |  |  |  |  |  |  |  |  |  |  |
| 0 | Vitvi06g01561\_t001 |  |  |  |  |  |  |  |  |  |  |  |  |
| 0 | Vitvi06g01562\_t001 |  |  |  |  |  |  |  |  |  |  |  |  |
| 0 | Vitvi06g01563\_t001 |  |  |  |  |  |  |  |  |  |  |  |  |
| 0 | Vitvi06g01564\_t001 |  |  |  |  |  |  |  |  |  |  |  |  |
| 0 | Vitvi06g01982\_t001 |  |  |  |  |  |  |  |  |  |  |  |  |
